# Penetrance estimation of Alzheimer disease in *SORL1* loss-of-function variant carriers using a family-based strategy and stratification by *APOE* genotypes

**DOI:** 10.1186/s13073-022-01070-6

**Published:** 2022-06-28

**Authors:** Catherine Schramm, Camille Charbonnier, Aline Zaréa, Morgane Lacour, David Wallon, CNRMAJ collaborators, Anne Boland, Jean-François Deleuze, Robert Olaso, ADES consortium, Flora Alarcon, Dominique Campion, Grégory Nuel, Gaël Nicolas

**Affiliations:** 1 Normandie Université, UNIROUEN, Inserm U1245, CHU Rouen, Department of Genetics and CNRMAJ, FHU-G4 Génomique, 22 boulevard Gambetta – CS 76183, Rouen, F-76000 France; 2 Normandie Université, UNIROUEN, Inserm U1245, CHU Rouen, Department of Neurology and CNRMAJ, FHU-G4 Génomique, Rouen, F-76000 France; 3 Université Paris-Saclay, CEA, Centre National de Recherche en Génomique Humaine, 91057 Evry, France; 4 MAP5, UMR-CNRS 8145, Paris University, 75270 Paris, France; 5 Department of Research, Rouvray Psychiatric Hospital, 76681 Sotteville-Lès-Rouen, France; 6 LPSM, CNRS 8001, Sorbonne University, 75005 Paris, France

**Keywords:** Alzheimer, *SORL1*, *APOE*, Penetrance, Lifetime risk, Expectation-maximization algorithm, Pedigree, Missing genotypes

## Abstract

**Background:**

Alzheimer disease (AD) is a common complex disorder with a high genetic component. Loss-of-function (LoF) *SORL1* variants are one of the strongest AD genetic risk factors. Estimating their age-related penetrance is essential before putative use for genetic counseling or preventive trials. However, relative rarity and co-occurrence with the main AD risk factor, *APOE*-ε4, make such estimations difficult.

**Methods:**

We proposed to estimate the age-related penetrance of *SORL1*-LoF variants through a survival framework by estimating the conditional instantaneous risk combining (i) a baseline for non-carriers of *SORL1-*LoF variants, stratified by *APOE-ε4*, derived from the Rotterdam study (*N* = 12,255), and (ii) an age-dependent proportional hazard effect for *SORL1-*LoF variants estimated from 27 extended pedigrees (including 307 relatives ≥ 40 years old, 45 of them having genotyping information) recruited from the French reference center for young Alzheimer patients. We embedded this model into an expectation-maximization algorithm to accommodate for missing genotypes. To correct for ascertainment bias, proband phenotypes were omitted. Then, we assessed if our penetrance curves were concordant with age distributions of *APOE*-ε4-stratified *SORL1-*LoF variant carriers detected among sequencing data of 13,007 cases and 10,182 controls from European and American case-control study consortia.

**Results:**

*SORL1-*LoF variants penetrance curves reached 100% (95% confidence interval [99–100%]) by age 70 among *APOE*-ε4ε4 carriers only, compared with 56% [40–72%] and 37% [26–51%] in ε4 heterozygous carriers and ε4 non-carriers, respectively. These estimates were fully consistent with observed age distributions of *SORL1-*LoF variant carriers in case-control study data.

**Conclusions:**

We conclude that *SORL1-*LoF variants should be interpreted in light of *APOE* genotypes for future clinical applications.

**Supplementary Information:**

The online version contains supplementary material available at 10.1186/s13073-022-01070-6.

## Background

The etiology of Alzheimer disease (AD) is multifactorial in the vast majority cases, including a high genetic component [1, 2]. The ε4 allele of the *APOE* gene (*APOE*-ε4) is currently considered as the main AD genetic risk factor, both in terms of frequency and effect size [3]. In addition, about 70 additional common single nucleotide polymorphisms (SNPs) have been associated with AD risk, each with a modest level of risk [4]. In addition, some families exhibit autosomal dominant AD due to a single pathogenic variant in either one of the *APP*, *PSEN1*, and *PSEN2* genes, most of them being associated with early-onset AD (EOAD, onset before 65 years) [5]. However, a large part of EOAD patients do not exhibit a clear autosomal dominant pattern of inheritance and/or a pathogenic *APP*, *PSEN1*, or *PSEN2* variant [6, 7]. In unrelated EOAD patients negatively screened for these genes despite a positive family history of EOAD, *SORL1* rare protein-truncating variants (PTVs) or missense predicted damaging variants were identified [8]. We showed that such *SORL1* rare variants are enriched in EOAD cases with a positive family history in a case-control study, with genome-wide significance at the gene level [9], and such results were subsequently extended to all AD cases with a clear effect on age at onset (AAO) [10–12]. However, due to the lack of segregation data in pedigrees, their mode of inheritance has yet to be determined. It thus remains unclear whether *SORL1* is the 4th autosomal dominant AD gene—hence with a single pathogenic variant being sufficient to cause AD in a given individual—or a strong risk factor.

Given the extreme rarity of such variants in controls and high odds-ratios (OR) [10], the questions of (i) their penetrance, i.e., the probability that a carrier actually develops AD at a given age, and (ii) a putative use for genetic counseling have been raised. Besides, in line with *APP*, *PSEN1*, and *PSEN2*, *SORL1* is involved in the production of Aβ peptides [13, 14], the aggregation of which is a critical triggering event in AD pathophysiology. This suggests that current preventive intervention trials applied to autosomal dominant AD [15] might also apply to *SORL1* presymptomatic carriers, provided that penetrance estimations are available.

Several approaches have been published to estimate the penetrance of genetic variants [3, 16–20]. While survival analyses relying on large prospective cohorts with sequencing data available may appear as the gold standard, they find themselves challenged, and thereby impractical, when assessing the penetrance associated with rare variants. Indeed, they require a large amount of sequencing data and a follow-up period long enough to allow the diagnosis of a sufficient number of cases, which is not yet available. To overcome this limit, methods have been developed to combine data from large prospective cohorts with large case/control studies and evaluate with more accuracy the lifetime risks or penetrance associated with genetic risk variants [3, 17]. Another option is to resort to family-based study designs. Indeed, focusing on families where the proband carries a specific variant of interest enriches the dataset in rare variant carriers. However, the downside is the risk of bias resulting from this ascertainment scheme, which could even be compounded by a selection on AAO. To overcome this issue, methods based on retrospective [18] or prospective [19, 20] likelihood were developed such as to condition the phenotype observation on the ascertainment process. A more “naive” but effective approach consists in computing the likelihood after exclusion of proband phenotypes [21]. Since DNA of all affected and unaffected relatives may not be available, all these approaches may be combined with the Elston-Stewart algorithm [22] in order to deal with the occurrence of missing genotypes in families. However, these methods remain scarcely used, probably because of their lack of flexibility and their complexity. More recently, Alarcon et al. [23] proposed a more flexible approach embedded in an expectation-maximization (EM) framework [24] that alternates between penetrance estimation (M-step) and a belief propagation step to impute missing genotypes (E-step). It was first used in the context of a monogenic disease but may be applied to complex diseases and used with parametric or non-parametric survival models combined with proband’s phenotype exclusion. Here, we further extended this method to (i) a digenic *scenario* combining a rare and a common risk factor and (ii) the integration of previously published data to robustly stratify for the common risk factor. We applied this strategy to assess the penetrance of AD associated with *SORL1* rare loss-of-function (LoF) variants in pedigrees where the AD-affected proband carries such a variant, with a baseline model stratified for common *APOE*-ε4 alleles derived from the Rotterdam Study [25]. We thus provide here the first penetrance estimates for *SORL1* rare variants stratified on the number of *APOE*-ε4 alleles.

## Methods

### Initial whole-exome sequencing dataset of unrelated probands

We considered all unrelated individuals (probands) carrying a *SORL1* rare (allele frequency < 1%) non-synonymous variant identified in a whole-exome sequencing (WES) dataset, generated from patients with a diagnosis of probable AD [26, 27] among patients referred to the National Reference Center for Young Alzheimer Patients (*Centre National de Référence Malades Alzheimer Jeunes*, CNRMAJ-Rouen, Rouen, France) in the context of a nation-wide recruitment.

In France, genetic screening of EOAD patients is centralized in a single expert genetics center (CNRMAJ-Rouen). Before the genetic screening, expert neurologists from the CNRMAJ of Rouen review all medical charts accompanying blood samples sent from all over the French territory, following recruitment by local physicians working in memory clinics, i.e., clinical departments, from public hospitals with a specialized activity in diagnostic assessment and care of patients with cognitive impairment.

Diagnoses are based on clinical examination by a physician and include personal medical and family history assessments, neurological examination, neuropsychological assessment, and neuroimaging arguments. In addition, cerebrospinal fluid (CSF) biomarkers (Aβ42, Tau, and P-Tau levels) are taken into account, when available, using previously described criteria [6].

Probands are either screened by Sanger sequencing and quantitative multiplex PCR of short fluorescent fragments (QMPSF) for pathogenic variants in *APP*, *PSEN1*, or *PSEN2* prior to WES or by the interpretation of WES data or both. Carriers of pathogenic variants are not included for WES or are secondarily excluded following WES analysis so that none of the patients included in this work is a carrier of a pathogenic variant in *APP*, *PSEN1*, and *PSEN2* as well as in a list of Mendelian dementia causative genes [6]. Overall, probands with WES data available considered in this work prior to *SORL1* variant selection have a neuropathological diagnosis (1%), a clinical diagnosis with CSF AD biomarkers indicative of AD (80%) [both allowing typical and atypical clinical presentations] or a clinical diagnosis of typical, amnestic form of AD (19%). Probands with a CSF not indicative of AD are not selected for WES.

Proband recruitment for WES was performed over a 25-year period, using the criteria defined by the National Institute of Neurological Disorders and Stroke and the Alzheimer Disease and Related Disorders Association (NINCDS-ADRA) criteria [26] and update [27] (National Institute of Aging and Alzheimer’s Association, NIAA), including CSF AD biomarkers, when available. The upper AAO criterion was originally established at 65 years (for clinical care, PHRC-GMAJ [*Programme Hospitalier de Recherche Clinique Génétique Maladie d’Alzheimer Jeune*], RBM-0259 [Recherche BioMédicale-0259], and ECASCAD [Exome – Clinical Application of SequenCing in Alzheimer Disease] studies), and this age criterion was set at 75 years during the 2018–2021 period (subset of ECASCAD study). During the 25-year period, patients or legal guardians provided informed written consent for genetic analyses and for providing medical information in a clinical setting and/or in a research setting (RBM-0259 study, authorized by the institutional review board [IRB] CPP [*Comité de Protection des Personnes*] Paris - Ile de France II, PHRC-GMAJ study, authorized by the IRB CPP Paris - Ile de France II, and the ECASCAD study, authorized by the IRB CPP Ouest 3). The retrospective analysis of already generated WES data for the current study was approved by our IRB CERDE (*Comité d’Ethique pour la Recherche sur Données Existantes et hors loi Jardé*, notification 2019-55).

### Genetic data processing and *SORL1* variants selection

All variants were detected in the context of WES performed in the National Center for Research in Human Genomics sequencing center (CNRGH, Evry, France) using Agilent Sureselect all exons human kits and Illumina sequencing technology, as described before [6]. Sequencing data were processed as previously described in Nicolas et al. [6], based on BWA, GATK, and SNPeff tools. We considered exome data from 1295 unrelated AD probands remaining after quality control as previously performed in our case-control association studies [9] and after exclusion of patients carrying a likely pathogenic or a pathogenic variant in a Mendelian dementia gene [6]. Minor allele frequencies (MAF) were annotated from the gnomAD database, non-Finnish European population [28]. We extracted germline (allelic ratio [0.25–1], all were eventually heterozygous) rare (MAF < 1%), non-synonymous, and splice region *SORL1* variants (NM_003105.6). We considered as PTV all nonsense, frameshift, and canonical splice site variants, in addition to splice region variants with a demonstrated effect on splicing (one described in Le Guennec et al. [29] and one following unpublished patient blood mRNA analysis). We also considered missense variants that were predicted damaging by all three software tools among Polyphen-2 [30], Mutation Taster [31], and SIFT [32], referred to as Mis3 variants, as candidates for the analysis. To lessen the heterogeneity of the set of variants under analysis, we restricted our analysis to the selection of *SORL1* loss-of-function variants, namely (i) high confidence PTVs (i.e., not affecting the last coding exon or 50 bp of the penultimate exon) and (ii) candidate *SORL1-*Mis3 variants that demonstrated an in vitro loss-of-function effect (Mis3-LoF). More precisely, Mis3-LoF were defined as rare Mis3 variants with at least two in vitro assays supporting such a loss-of-function effect and including at least one Aβ peptide measurement. We retained the following three Mis3 variants as non-ambiguously Mis3-LoF in this conservative approach: c.994C>T,p.(R332W), c.1960C>T,p.(R654W) [33], and c.1531G>C,p.(G511R) [34]. Hereafter and for penetrance estimations, we use the term “LoF” when considering PTV plus Mis3-LoF as a group.

### Family history and genetic investigations in relatives

Overall, we included 27 probands carrying either a *SORL1-*LoF in this study, and we contacted all families in order to extend pedigree information, through the participation of the patient or legal guardian, after providing written informed consent to the RBM-0259 study. Clinical status and AAO for affected relatives or current age for unaffected relatives were obtained at least for siblings and parents of probands and, when possible, for aunts, uncles, and grandparents. Both parental sides were systematically investigated even when the presence of an affected parent suggested a unilateral transmission. When the clinical examination was not possible, a phone interview was performed. Disease status was set as possible or probable AD according to the NINCDS-ADRDA criteria [26], missing, or unaffected based on available clinical information from medical charts for affected relatives and from available clinical information or phone interviews for unaffected relatives. Every family was contacted personally by one of the clinicians from CNRMAJ-Rouen (AZ, ML, GN, DC, DW), to check and complete the information on pedigrees. AAO were defined as the age of a given affected relative when cognitive impairment was first noticed by a close relative, similarly to probands and as performed in case-control studies. We have put an important effort into family interviews, based on those used in observational studies and clinical trials in autosomal dominant AD [35] to help families remember the last time a given relative was seen apparently unaffected, and the first time they were noticed as affected, by using references and specific events (e.g., specific family events, birthdays). We interviewed at least one informant from the same generation as the proband (spouses, unaffected sibs, spouses of affected sibs) and, when possible, contacted multiple informants from the same family to double-check the information on the previous generations. Direct informants from previous generations were sought (e.g., unaffected parent, unaffected uncle/aunts, or spouses of affected uncles/aunts). When possible, access to clinical charts was requested, following the agreement of the next of kin.

In addition, we collected blood samples from affected and unaffected relatives with informed written consent and performed Sanger sequencing to search for the *SORL1* variant segregating in the family and to determine the *APOE* genotype. Affected and unaffected relatives or legal guardians provided informed written consent for this study including genetic analyses (RBM-0259). In the end, we obtained clinical information for 307 relatives with age ≥ 40 years and genotyping information for 45 relatives including 20 carriers of a *SORL1-*LoF variant.

### Statistical analyses

#### Modeling

**Fig. 1:**
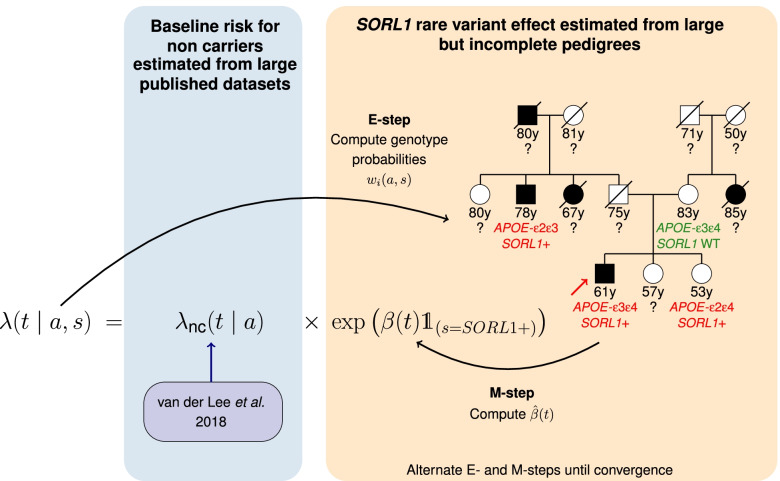
Overview of the method for estimating the piecewise constant hazard model. *λ*(*t* | *a*, *s*) refers to the instantaneous risk to develop the disease depending on age *t* and genotype (*a*,*s*) ∊ *APOE* × *SORL1*. *λ*_nc_(*t* | *a*) refers to the specific instantaneous risk associated with non-carriers of the *SORL1* variant of interest stratified on *APOE* genotype and derived from the Rotterdam Study [25]. *β(t)* refers to the additional effect of the *SORL1* variant. *λ*_nc_(*t* | *a*) and *β(t)* are both piecewise constant over time. E/M-steps refer to expectation/maximization steps. *w*_*i*_*(a, s)*, individual weight updating at each E-step iteration referring to the posterior probability distribution of individual *i* for combined genotype *(a,s)*; y, years; *SORL1*+, carrier of the variant of interest in *SORL1* gene; *SORL1* WT, wild type for *SORL1* (non-carriers of the variant of interest); ?, unknown genotype. The red arrow indicates the proband

As usually done in survival analysis, we modeled the age-related penetrance of AD associated with *SORL1-*LoF variants through the hazard function *λ(t | a, s)*, i.e., the instantaneous risk of developing AD at time *t* conditional on not having developed it before for individuals of *APOE* status *a* and *SORL1* status *s.* We proposed a piecewise constant hazard model taking the following parametric form:

1 $$\lambda \left(t\ |\ a,s\right)={\lambda}_{\mathrm{nc}}\left(t\ |\ a\right)\ \exp \left(\beta (t){\mathbf{1}}_{s= SORL1+}\right)$$

where *λ*_nc_*(t | a)* corresponds to the baseline risk for non-carriers of the *SORL1*-LoF variant, depending only on *APOE* and *β(t)* corresponds to the additional effect of *SORL1*-LoF variants (Fig. 1). Since the effect of *APOE* alleles has already been deeply studied through large prospective cohort studies, we relied on published results to define the *APOE*-stratified baseline of our model [25]. Given the extreme rarity of *SORL1-*LoF variants, we made the assumption that this population-based baseline models AD risk for non-carriers. See Additional file 1: Supplementary methods for detailed computation of *λ*_nc_*(t | a)* from literature. Then, our model combines this literature-based baseline with an age-dependent proportional effect for *SORL1-*LoF variant carriers (*β(t)*), assumed piecewise constant over time, independent from *APOE,* and estimated from our pedigrees. Since the pedigrees include relatives with missing genotypes, we applied an EM algorithm to take full advantage of the available information on the whole pedigree. Indeed, this iterative algorithm, alternating E- and M-steps to maximize the likelihood of the model, is efficient when the information is partially missing (here the *APOE* and *SORL1* genotypes). This algorithm alternates until convergence between replacing unknown genotypes with individual weights *w*_*i*_(*a*, *s*) corresponding to the posterior probability that subject *i* should carry genotype {*a*, *s*} (genotype posterior distributions) based on current age-related penetrances (E-step) and estimating the *β*(*t*) coefficients to update the age-related penetrances for each possible genotype based on observations and previously computed individual genotype distributions *w*_*i*_(*a*,*s*) (M-step). We denote respectively by ${w}_i^m\left(a,s\right)$ and ${\hat{\beta}}^m(t)$ the values of *w*_*i*_(*a*, *s*) and $\hat{\beta}(t)$ at the *m*^th^ iteration of the EM algorithm.

##### E-step

Denote by ***G*** the ensemble of the 36 possible *APOE*=*a*×*SORL1*=*s* genotype combinations. This set differentiates paternal from maternal alleles for the purpose of genotype propagation through pedigrees. It accounts for every *APOE* alleles (*ε*2, *ε*3, *ε*4) and denotes by 1 (respectively 0) the presence (resp. absence) of a *SORL1*-LoF variant (*a* ∈ {^“^*ε*2*ε*2^”^, ^“^*ε*3*ε*2^”^, ^“^*ε*4*ε*2^”^, ^“^*ε*2*ε*3^”^, ^“^*ε*3*ε*3^”^, ^“^*ε*4*ε*3^”^, ^“^*ε*2*ε*4^”^, ^“^*ε*3*ε*4^”^, ^“^*ε*4*ε*4^"^} and *s* ∈ {^“^00^”^, ^“^10^”^, ^“^01^”^, ^“^11^”^}). The E-step consists in updating for each subject *i* and each genotype combination {*a*, *s*}∈ ***G***, the posterior probability *w*_*i*_(*a*, *s*) that subject *i* should carry genotype combination {*a*, *s*} conditional on **ev** the evidence defined as the probability of observing the data according to the genotype and $\hat{\beta}{(t)}^m$, the last estimation of *β*(*t*).

$${w}_i^m\left(a,s\right)=\mathbb{P}\left(\mathrm{Genotype}\ \mathrm{combination}\ \mathrm{of}\ i\ \mathrm{is}\ a,s\ |\ {\mathbf{ev}}_i^m\left(a,s\right)\right)$$

These probabilities are computed using an ad hoc C++ implementation of belief propagation in pedigrees (**bped**) [36] extended from Alarcon et al. [23] to the specific case of *APOE*×*SORL1* genotypes.

Indeed, the **bped** program was initially designed for a single bi-allelic locus. The program takes as input a pedigree structure and an evidence file and returns both the log-likelihood and the marginal posterior genotypic distribution of all individuals. The evidence file can incorporate any kind of data (binary outcome, time-to-event outcome, etc.) as long as the conditional probabilities $\mathbb{P}$(outcome | genotype) are provided as real numbers. The marginal posterior probabilities are all we need to implement an EM algorithm. For the present application, **bped** had to be extended to two loci (one with 3 alleles, the second with 2 alleles, **bped3alleles2alleles**) which is a straightforward extension of the initial program [36].

In our case, the evidence file contains a *n*×#***G*** matrix of evidence with:

$$e{v}_i^m\left(a,s\right)=\mathbb{P}\left({T}_i\ |\ a,s;{\hat{\beta}}^m(t)\right)=\left\{\begin{array}{c}{\hat{S}}^m\left({T}_i\ |a,s\right)\kern0.75em \mathrm{if}\ {\delta}_i=0\\ {}\begin{array}{cc}{\hat{S}}^m\left({T}_i |a,s\right){\hat{\lambda}}^m\left({T}_i|a,s\right)& \mathrm{if}\ {\delta}_i=1\end{array}\end{array}\right.$$

where *T*_*i*_ is the observed time associated with the status *δ*_*i*_ equaling 1 if the subject *i* has developed AD at age *T*_*i*_ and 0 otherwise, and $\hat{S}\left(t\ |a,s\right)$ is the estimation of the survival function

$$S\left(t\ |a,s\right)=\exp \left(-{\int}_0^t\lambda \left(u\ |a,s\right) du\right).$$

Of note, the evidence and thus posterior probabilities are set to 0 for all genotype combinations {*a*, *s*} discordant with the available information.

##### M-step

We assume that *β*(*t*) is piecewise constant over *J* intervals defined by *J* − 1 cutoffs *τ*_1_, …, *τ*_*J* − 1_ such that ∀*j* ∈ {1, …., *J*}, *β*(*t*) = *β*_*j*_ if *t* ∈ [*τ*_*j* − 1_, *τ*_*j*_[, *τ*_0_ = 0 and *τ*_*J*_ =  + ∞. The M-step consists in estimating *β*_*j*, *j* ∈ {1, …, *J*}_ that maximizes the likelihood of our data. Because of unobservable genotypes, we instead maximized $Q\left(\ \beta (t)\ |\kern0.50em {\hat{\beta}}^{m-1}(t)\ \right)$ the expected value of the log-likelihood function with respect to the current conditional distribution of unobserved genotype combinations {*a*, *s*} given evidence and the current estimates of the parameters ${\hat{\beta}}^{m-1}(t)$:

$${\hat{\beta}}^m(t)=\arg \underset{\beta (t)}{\max }\ Q\left(\beta (t)|\ {\hat{\beta}}^{m-1}(t)\right)=\arg \underset{\beta (t)}{\max }\ \sum_{i=1}^n\ \sum_{\left\{a,s\right\}\in \boldsymbol{G}}{w}_i^{m-1}\left(a,s\right)\ \left(-\Lambda \left({T}_i\ |a,s\right)+{\delta}_i\ \log \lambda \left({T}_i|\ a,s\right)\right)$$

where Λ(*t* |*a*, *s*) denotes the cumulative risk until time *t* for individuals of *APOE* status *a* and *SORL1* status *s*. Besides, note that because the additive *SORL1* effect only appears in the survival and hazard function of *SORL1* carriers, the sum on ***G*** simplifies into a sum on ***G***_**1**_, the subset of genotype combinations that include a *SORL1*-LoF variant. In the end, the parameters ${\hat{\beta}}_{j,j\in \left\{1,\dots .,J\right\}}^m$ that maximize the *Q* function are straightforwardly obtained by solving for each *j* ∈ {1, …., *J*},$\frac{\partial Q\left(\beta (t)|{\hat{\beta}}^{m-1}(t)\ \right)}{\partial {\beta}_j}=0$ such that ∀*j* ∈ {1, …., *J*}:

$$\widehat\beta_{j}^{m}=\log\left(\frac{{\sum_{\begin{array}{c}i=1\\T_{i}\in\left[\tau_{j-1};\tau_{j}\right]\end{array}}^n}{\sum_{\left\{a,s\right\}\in{\boldsymbol G}_{\mathbf 1}}}w_{i}^{m-1}\left(a,s\right)\delta_{i}}{{\sum_{\begin{array}{c}i=1\\T_{i}\geq\tau_{j-1}\end{array}}^n}{\sum_{\left\{a,s\right\}\in{\boldsymbol {G}}_{\mathbf {1}}}}w_{i}^{m-1}(a,s)\left[\Lambda_{nc}^{m-1}\left(min(T_{i},\tau_{j})\vert a,s\right)-\Lambda_{nc}^{m-1}\left(\tau_{j-1}\vert a,s\right)\right]}\right)$$

In other words, the hazard ratio $\exp({\hat{\beta}}_j)$ associated with *SORL1*-LoF variants on the *j*^th^ interval may be interpreted as the ratio of the subjects developing the disease within the time-interval *j* over the time subjects at risk during this time interval taking into account the *APOE* effect. The choice of cutoffs was determined by the Bayesian Information Criterion (BIC), and confidence intervals were computed using bootstrap taking into account variability associated with the estimation of both *λ*_nc_(*t*| *a*) and *β*(*t*). See Additional file 1: Supplementary methods for more details.

It is important to notice that (i) to compensate for the rarity of *APOE-ε*2, *APOE* genotypes are modeled in Eq. (1) through the number of *APOE-ε*4 alleles and therefore averaged into three categories: no allele *ε*4, heterozygous *ε*4, and *ε*4*ε*4 individuals, and (ii) *SORL1* status “01,” “10,” and “11” are all considered as *SORL1+* (*SORL1*-LoF carriers) and contribute equally to the estimation of *β*(*t*). Thus, E-step allows for 36 possible genotypes, and M-step relying on Eq. (1) considers only six differential effect genotypes {*ε*4 non-carriers, *ε*4 heterozygous carriers, *ε*4*ε*4 carriers}×{*SORL1*-LoF non-carriers, *SORL1*-LoF carriers} resulting into six age-related penetrance curves. Of note, relatives with missing phenotype and probands do not contribute to the M-step, but they may inform on the pedigree structure during the E-step.

We performed a simulation study in order to assess the robustness of our methodology. Detailed methods and description of the simulation study are available in Additional file 1: Supplementary methods. Finally, we proposed a sensitivity analysis excluding the missense variants.

### Assessing ages of *SORL1*-LoF variant carriers per *APOE* genotype in a case-control study

To verify that our penetrance estimates per *APOE* genotype were consistent with large datasets of unrelated individuals, we interrogated the discovery dataset of the largest exome/genome case-control series, combining data from the Alzheimer Disease European Sequencing (ADES) and American Alzheimer Disease Sequencing Project (ADSP) consortia [11, 37], to which we added more recent cases from the ECASCAD study from France (*N* = 545), reaching a total of 13,007 cases and 10,182 controls. We then extracted carriers of *SORL1-*LoF variants as defined above and sorted them based on their case/control status and their *APOE* genotypes. Ages at last examination of controls and AAO for cases were then compared to the expected penetrance identified in our family-based study. We assessed the likelihood of the observations of *SORL1*-LoF carriers in the case-control dataset given our penetrance model and compared it to the likelihood obtained under a model stratified on *APOE*-e4 but without the *SORL1*-LoF effect (*β*(*t*) = 0), using Akaike and Bayesian Information Criteria (AIC/BIC).

## Results

### Simulation study

**Fig. 2:**
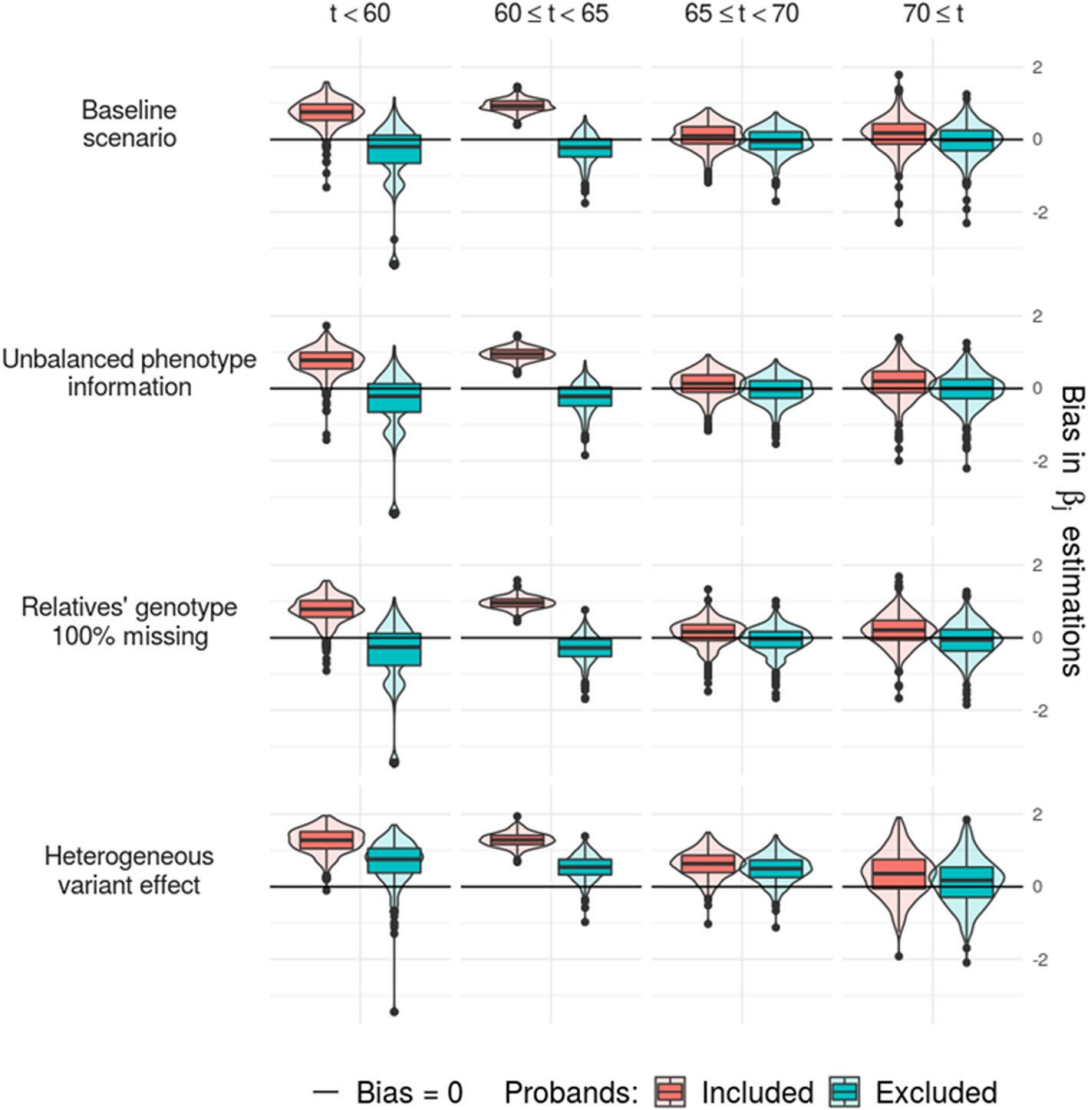
Estimation of bias in our simulation study. Bias was estimated for each of the four constant parameters (in columns) of the piecewise constant *β(t)* referring to the additional effect of the *SORL1* variant of interest through 4 *scenarios* of simulation (in rows). The results are provided for probands included and excluded from the analysis during the maximization step of the EM algorithm. For the baseline *scenario*, we generated 27 families mimicking what we observed in our dataset in terms of *SORL1-*LoF variant effect, ascertainment, and available genotypes. Then, the model was challenged through three additional *scenarios*: (i) Unbalanced phenotype information: if one of the parents was affected, we removed all the phenotypic information of the parental branch with the unaffected parent. (ii) Relatives’ genotypes 100% missing: we removed all information about relatives’ genotypes. (iii) Heterogeneous variant effect: instead of generating age at onset based on a constant variant effect, we generated age at onset based on a normal distribution of the variant effect with a variance equaling to 1. A bias greater than 0 indicates an overestimation and a bias lower than 0 indicates an underestimation of the risk associated with *SORL1*-LoF variants

Our simulation study confirmed that our methodology was robust to various patterns of genotype and phenotype missingness, in particular, even in the presence of unbalanced pedigree ascertainment (Fig. 2 and Additional file 1: Figs. S3-S9). Exclusion of the proband phenotype at the M step (*SORL1* hazard ratio computation) efficiently reduced the bias resulting from AAO-based ascertainment. The Bayesian Information Criterion (BIC) was able to discriminate models and identify the best time cutoffs for the piecewise constant function *β(t)* (Additional file 1: Fig. S10). Based on our simulations, we concluded that the model was very robust to any putative tested bias, except when variant effects were not homogeneous across families, which might lead to an overestimation of the average variant effect. This limitation justifies our strict focus on families with non-ambiguous *SORL1-*LoF variants.

### Description of the pedigrees

A total of 27 families carried at least one non-ambiguous LoF variant (21 families with one PTV, one family with 2 PTVs, and 5 families with a Mis3-LoF). Although most of the LoF variants were each private to one family, the p.W804* (c.2412G>A; PTV) and p.R654W (c.1960C>T; Mis3-LoF) variants were respectively observed in two and three apparently unrelated families, even though they were both not observed in the gnomAD database. Among the LoF variants, only 3 were observed at least once in gnomAD: p.R332W (c.994C>T, MAF = 1.1 × 10^−5^), p.R866* (c.2596C>T; MAF = 1.9 × 10^−5^), and p.R1207* (c.3619C>T; MAF = 2.2 × 10^−5^). See Additional file 1: Table S1 for a detailed list of variants. In LoF families, proband AAO varied from 48 to 70 years (mean 58 years). Two probands (7%) were *APOE*-ε4ε4 carriers (AAO 58, 60), 18 (67%) were ε4 heterozygous carriers (AAO from 50 to 66, mean 58), and 7 (26%) were ε4 non-carriers (AAO from 48 to 70, mean 56). The diagnosis of AD was supported by clinical and imaging arguments in all probands, to which 19 also had a cerebrospinal fluid AD biomarkers profile consistent with the AD diagnosis.

In addition, informative phenotypes (disease status and age ≥ 40 years) and genotypes (*APOE* and *SORL1*) were obtained for 307 and 45 relatives, respectively (Table 1). They were collected in both paternal and maternal branches, whatever the apparent disease transmissions (Additional file 1: Fig. S1). LoF families included a median of 11 informative phenotypes (Q1–Q3 8.5–17; min–max 4–24) over 3 generations (Q1–Q3 3–3; min–max 2–5) and 2 genotypes (Q1–Q3 1–4; min–max 1–6) encompassing data of the proband. Genotypes of relatives were known (genotyped individual or obligate carrier) for 12 affected carriers (AAO from 55 to 78, median 68, Q1–Q3 64.5–74), 12 unaffected carriers (age from 42 to 95, median 66, Q1–Q3 52–68.25), 4 affected non-carriers (AAO 64, 68, 70, and 75), and 21 unaffected non-carriers (age from 37 to 86, median 68, Q1–Q3 57–70). See pedigrees in Additional file 1: Supplementary results.

**Table 1:** Description of informative phenotypes and genotypes available in the 27 families with a LoF variant

	Probands	Affected relatives		Unaffected relatives	
**Informative phenotypes,*N***	**27**	**61**		**246**	
Males/females, *N* (%)^a^	11 (41%)/16 (59%)	16 (26%)/45 (74%)		121 (50%)/120 (50%)	
Age, y, median (Q1–Q3)	58 (52.5–61)	67 (63–74)		68 (55.25–78)	
**Available genotypes,*N***	**27**	**13**		**32**	
*SORL1* carriers, *N* (%)	27 (100%)	9 (69%)		11 (34%)	
*APOE* × *SORL1* (+ vs WT)	+	+	WT	+	WT
ε4 non-carriers, *N* (%)	7 (26%)	6 (46%)	3 (23%)	7 (22%)	14 (44%)
ε4 heterozygous carriers, *N* (%)	18 (67%)	3 (23%)	1 (8%)	2 (6%)	6 (19%)
ε4ε4 carriers, *N* (%)	2 (7%)	0 (0%)	0 (0%)	2 (6%)	1 (3%)

We considered as informative individuals for the phenotype, all those having well-established disease status as well as AAO or censoring above 40 years. Genotypes were available for 71 (21%) of individuals with informative phenotype as well as for one unaffected relative (censoring age 37 years, genotype *APOE*-ε4 heterozygous, *SORL1* WT)

*N* number, *y* years, *Q1* first quartile, *Q3* third quartile, *WT* wild type

^a^Percentages are given over known gender

### Genotype probabilities and penetrance estimates

**Fig. 3:**
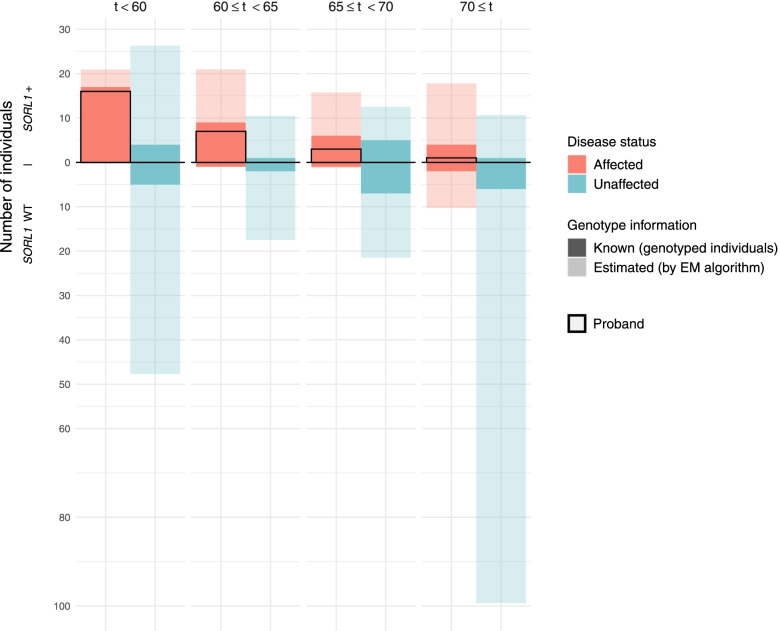
Number of affected and unaffected individuals according to their age and their *SORL1* status after genotyping imputation. This graph was obtained for all individuals with AAO or censoring above 40 years. It represents the number of carriers (upper part) and non-carriers (lower part) of the *SORL1-*LoF variant according to their disease status and age intervals (AAO for probands and affected relatives and censoring for unaffected relatives). Transparency differentiates available genotypes (already known, including those of probands) from those estimated at the end of the algorithm

During the EM procedure, genotype probabilities were attributed to each participant. Final genotype posterior distributions combined with known genotype information led to an expected total number of relatives aged ≥ 40 years carrying a *SORL1-*LoF variant of 108.4 (35% of relatives aged ≥ 40 years; Fig. 3). Additional file 1: Table S3 provides detailed estimated genotypes.

**Fig. 4:**
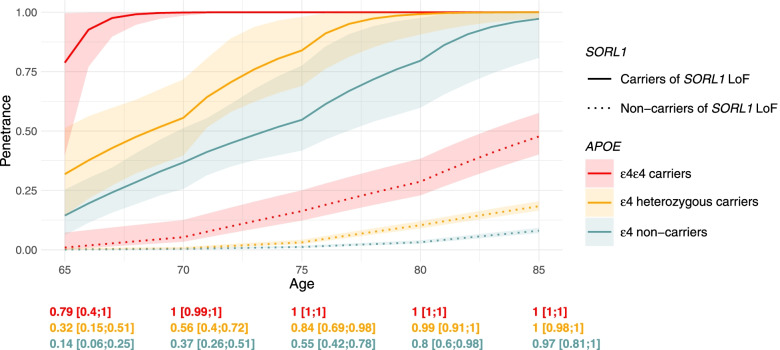
Age-dependent penetrance for carriers and non-carriers of a SORL1 LoF variant. The penetrance is displayed with its 95% confidence interval according to the number of *APOE*-ε4 allele from 65 to 85 years of age. Curves for non-carriers and their confidence intervals were obtained from our estimation of *λ*_nc_(*t* | *a*) based on the Rotterdam Study. Data from pedigrees were censored at 85 years. Confidence intervals for *SORL1-*LoF variant carriers were obtained from the 2.5th and 97.5th quantiles of 500 bootstrap iterations. Penetrance values at 65, 70, 75, 80, and 85 years of age for carriers of the *SORL1-*LoF variant are displayed below the figure

Based on BIC (Additional file 1: Table S2), we retained a piecewise constant *β(t)* with cutoffs at respectively 60, 65, and 70 years (*β(t)* = 3.5, 95%CI [1.5; 5.0] for *t* < 60 years; *β(t)* = 6.7, 95%CI [5.1; 8.0] for 60 ≤ *t* < 65; *β(t)* = 4.7, 95%CI [3.9; 5.4] for 65 ≤ *t* < 70 and *β(t)* = 3.7, 95%CI [2.6; 4.7] for 70 ≤ *t*) leading to an increased age-dependent penetrance for carriers versus non-carriers of *SORL1-*LoF variants. From our piecewise constant hazard model, we derived the penetrance function stratified on *APOE*-ε4 status and we observed a full penetrance by the age of 70 years for ε4-ε4 carriers only, whereas ε4 heterozygous carriers and ε4 non-carriers reached respectively 56% (95%CI [40%; 72%]) and 37% (95%CI [26%; 51%]) penetrance (Fig. 4). Of note, full penetrance was reached 10 years later for ε4 heterozygous carriers (95%CI [91%; 100%] by age 80) and after 85 years old for ε4 non-carriers (95%CI [81%; 100%] by age 85). The sensitivity analysis excluding the missense variants showed similar results (Additional file 1: Fig. S13).

### Assessing ages of *SORL1*-LoF variant carriers per *APOE* genotype in a case-control study

Among the 13,007 cases and 10,182 controls available, 77 carried a *SORL1-*LoF variant following the same definition as the one used in our pedigree study. Seventy-four of these were cases, and only three controls carried such a variant, suggesting very high penetrance. Controls, aged 75, 89, and ≥ 90 at the last visit, were all *APOE*-ε3 homozygous. In addition, AAO in cases carrying a *SORL1-*LoF variant were very informative, as all *APOE*-ε4 homozygous cases had an AAO before 65 (*N* = 7, age range 50–65, median = 60), all *APOE*-ε4 heterozygous cases had an AAO before 76 (*N* = 41, age range 47–76, median = 60), and the AAO of *APOE*-ε4 non-carriers ranged from 51 to above 90 (*N* = 26, median = 65), with 4 developing symptoms after 80 years of age. The distribution of AAO by *APOE* genotype for the 74 cases carrying a *SORL1*-LoF variant is provided in Additional file 1: Fig. S14. In the context of an enriched population of *SORL1*-LoF carriers in early-onset patients, we estimated a mean anticipation of AAO of 7.28 years for heterozygous ε4 carriers compared to ε4 non-carriers (95%CI = [1.85; 12.71], Welch-test *p*-value = 0.01) and of 10.31 for ε4ε4 carriers compared to ε4 non-carriers (95%CI = [3.59; 17.02], Welch-test *p*-value = 0.004). The case-control dataset did not include any *SORL1-*LoF and *APOE*-ε4 homozygous patients (respectively control) with AAO (respectively age at last examination) > 70 years, nor *APOE*-ε4 heterozygous patients (resp. control) with AAO (resp. age at last examination) > 80 years, which would be inconsistent with our penetrance curves. Finally, based on likelihood, our penetrance model computed from pedigree better fits the observations of all *SORL1*-LoF carriers in the case-control study (Log-likelihood = -327, AIC = 662, BIC = 671) than the null model considering no effect of *SORL1*-LoF variant (log-likelihood = -606, AIC/BIC = 1213).

Thus, all these observations are fully compatible with the estimates from our family-based study.

## Discussion

We investigated the age-related penetrance of AD associated with *SORL1-*LoF variants, using a family-based approach and stratifying for the common risk factor *APOE*-ε4. Consistent with previous results of case-control studies, our model indicates that *SORL1-*LoF variants confer a high risk before 65 years and thus are strongly associated with EOAD [10, 12]. The risk remained high until 70 years, which is compatible with an association of such variants with late-onset AD among younger patients. However, our results suggest that the penetrance associated with *SORL1-*LoF variants should be interpreted in light of the carrier’s *APOE* genotype. Indeed, in our model, the penetrance for *SORL1-*LoF carriers was complete by age 70 only among ε4ε4 carriers, whereas the curve for *SORL1-*LoF and ε4 heterozygous carriers reached complete penetrance 10 years later and even later for ε4 non-carriers.

*APOE* has already been investigated as a modifier of AD AAO regarding the *PSEN1* p.E280A and *APP* V717I pathogenic variants [38, 39]. However, these variants, as most pathogenic *APP*, *PSEN1*, or *PSEN2* variants, retained full penetrance before 65 by themselves, such that the effect of *APOE*-ε4 on AAO was modest. Hence, *APOE* genotyping is not considered as clinically meaningful upon genetic counseling in such families. In contrast, *SORL1-*LoF variants are less penetrant by themselves and thus *APOE*-ε4 substantially modifies AAO for carriers, suggesting that presymptomatic testing of *SORL1-*LoF variants should not be proposed regardless of *APOE* genotyping even though such digenic presymptomatic testing is not usual in clinical genetics.

Here, we used stringent criteria to restrict our analysis to a set of variants with homogeneous effect (PTV + Mis3-LoF), in accordance with our simulation study. Indeed, since Mis3 variants were defined solely through bioinformatics prediction tools in case-control studies, we can expect that there is some diversity of the variant effects on the encoded SorLA protein function towards Aβ secretion [33], so that our estimates may not be directly translated to all Mis3 *SORL1* variants. Moreover, the penetrance curves reflect well the penetrance associated with one *SORL1*-LoF variant and may not predict what is expected for a carrier of bi-allelic *SORL1*-LoF variants. Although the EM algorithm allows for bi-allelic carriers in the pedigrees, the probability to observe such a carrier is too rare to be expected among relatives (posterior probability ranges from 0 to 2.18 × 10^−4^ leading to an expected total number of such carriers < 1). Of note, one single individual was compound heterozygous for two *SORL1*-LoF variants in our family cohort (previously reported in [29]), but, as he is a proband, he did not contribute to the estimation of *β*(*t*) and, as he has no sibpair, no relative was likely to be a bi-allelic LoF variant carrier.

In our study, we generated familial data based on a nation-wide recruitment that is probably amongst the largest datasets on *SORL1-*LoF families, as this gene is not systematically screened in a clinical setting. Despite this, the number of families remained limited, especially after restriction to families with a non-ambiguous LoF variant. However, the EM approach allowed us to reach sufficient power to provide meaningful estimates. A limitation of our study may be the absence of possible replication in a large number of pedigrees as there is no comparable series of extended pedigrees, to our knowledge. We thus assessed the compatibility of our curves in the largest dataset of exomes/genomes of AD patients and controls available. AAO for cases and age at last visit for controls were all consistent with our penetrance curves.

In this study, we relied on the EM framework for penetrance calculation in incomplete pedigrees, first used by Alarcon et al. [23] and extended to our digenic problematic (E-step extension) and parametric modeling (M-step changes). We finally incorporated it into a 2-step methodology also including a baseline estimation from published data. The major interest for this methodology is the combination of several modules that all may be independently modified and improved for further family-based analyses and should open the door to the analyses of (often incomplete) pedigrees, whatever the gene and the disease. Moreover, our model might be further developed to better take into account the following points and thus help refining estimations and apply to other diseases: controlling for ascertainment biases in more suitable way than removing probands phenotypes information, applying other strategies for cutoff piecewise constant functions, adjusting for family clusters or other genetic factors and taking death as a competing risk (see Additional file 1: Supplementary methods and discussion about the model). In addition, our study deeply relied on AAO of affected relatives following systematic family interview. Although we put a lot of effort in verifying such information, it relied on the memories of relatives and this may thus introduce a bias, especially for relatives from previous generations and when the informant was not from the same generation. However, the compatibility of our results with the observation of AAO for *SORL1*-LoF carriers in the case-control studies does not suggest a systematic memory bias towards older or towards younger ages.

In our model, it was not possible to draw penetrance curves before the age of 65, due to the lack of large cohort data regarding *APOE* effects before 65. The curve crossed the age of 65 at the 79% [40–100%], 32% [15–51%], and 14% [6–25%] levels of penetrance respectively for the ε4 homozygous, heterozygous, and ε4 non-carriers, suggesting that a number of *SORL1*-LoF carriers develop first symptoms before 65. Consistent with this observation, the minimal observed AAO among probands was 48 years. Thus, although our study sheds light on the right side of the curve, i.e., complete or incomplete penetrance depending on *APOE* genotypes, further work is needed to better predict AAO overall, more specifically before 65.

In our model, we stratified on the number of *APOE*-ε4 alleles given the frequency of this allele and its moderate-to-high impact. In addition to *APOE* and *SORL1*, other types of variants can influence AD risk and their co-occurrence with a *SORL1-*LoF variant might also influence AAO and penetrance curves. Common variants identified in GWAS and rare variants identified in exome sequencing studies, mainly in the *ABCA7* and *TREM2* genes, can thus modulate the individual risk. Among common variants associated with AD, several map to the *SORL1* locus itself [40]; differential allele expression levels having been proposed as a putative mechanism [41]. Thus, the haplotype context of the rare LoF variant could theoretically influence AAO. However, ORs remain rather modest (OR = 0.84, [0.81–0.87] in the latest GWAS for rs11218343 [4]), suggesting a putatively small modifying effect. Recently, genetic risk scores (GRS) have been developed, gathering the individual small effect of multiple GWAS SNPs, albeit with some diversity in the methods and selection of SNPs. The effect of GRS as modulators of the *APOE* genotype has been measured [25]. Such an effect remains modest as compared to rare variants, so that GRS-*APOE* lifetime risk shall not be used at the individual level without including rare variants. Adjusting the effect of rare *SORL1* variants on that GRS-stratified *APOE*-ε4 is however an interesting perspective, conditional to the feasible extension of the E-step to such a multigenic model, i.e., allowing for the imputation of multiples SNPs for GRS estimation in relatives without drastically increasing computational cost. However, given the large risk load already conferred by *APOE* and *SORL1*, we do not expect that GRS may play a clinically relevant role in *SORL1*+*APOE*-ε4 carriers. This might be different in case of co-occurrence of an *ABCA7* or a *TREM2* risk variant. ORs of most deleterious variants of either gene are in the order of magnitude of 1.7–4 [42]. Thus, an oligogenic model taking into account these genes would be welcome. However, the rarity of carriers of multiple unambiguously deleterious/associated *TREM2*, *ABCA7*, and *SORL1* variants make such an oligogenic model challenging to assess with sufficient power.

## Conclusions

We propose here digenic penetrance estimations for *SORL1-*LoF variants together with *APOE*-ε4 alleles. Our estimation is based on recruitment likely closer to that of genetic counseling requests in families than to the general population, and our estimates may thus be taken into account when an asymptomatic relative may request information following the identification of a *SORL1-*LoF variant found in a proband. We consider that *SORL1* presymptomatic testing should not be performed regardless of *APOE* genotyping, given the large modifying effect in these families.

## Supplementary Information

**Additional file 1: Supplementary methods.** Statistical modeling, simulation study. **Supplementary results.** Comparison of bootstrap and jackknife procedures, results from the simulation study, pedigrees information. Supplementary discussion about the model. **Fig. S1.** Number of informative phenotypes available in maternal versus paternal branch over the 27 LoF families. **Fig. S2.** Age distribution in LoF families. **Fig. S3.** Results of simulations in scenario A when missingness does not depend on age. **Fig. S4.** Results of simulation in scenario 1 when missingness depends on age. **Fig. S5.** Results of simulation in scenario B. **Fig. S6.** Results of simulation in scenario B when missingness is related to one family branch only (the one with the lower number of cases). **Fig. S7.** Results of simulation in scenario B when missingness is related to one family branch only (the one with parent not being a case). **Fig. S8.** Results of simulation in scenario C with normal random effect. **Fig. S9.** Results of simulation in scenario C with gamma random effect. **Fig. S10.** Results of simulations in scenario D. **Fig. S11.** Posterior probability of carrying a variant in the baseline scenario over 500 simulated datasets. **Fig. S12.** Distribution of parameters obtained over 500 iterations of bootstrap and jackknife methods. **Fig. S13.** Age dependent penetrance for carriers and non-carriers of a *SORL1* PTV. **Fig. S14.** Distribution of AAO for *SORL1*-LoF carriers being AD cases in the case-control dataset according to their *APOE* genotype. **Table S1.***SORL1* variants included in our family cohort. **Table S2.** Comparison of models based on the Bayesian Information Criterion (BIC). **Table S3.** Expected total number of individuals by genotype. List of CNRMAJ collaborators. List of ADES collaborators.

## Data Availability

The code is available at https://github.com/U1245/Penetrance-estimation-from-family-cohort-in-AD-for-SORL1-APOE [36]. All pedigrees that were used for this study are provided in Additional file 1: supplementary results (§ pedigrees information). They are displayed in tree format including available *SORL1*-*APOE* genotypes, disease status, and age (onset or censoring [last visit or death] respectively for affected and unaffected individuals). Exome sequencing data are personal, potentially identifiable data, which we are not allowed to share publicly based on patients’ consents and IRB approvals.

## Abbreviations

LoF: Loss-of-function
AAO: Age at onset
Aβ: Amyloid β
AD: Alzheimer disease
ADES: Alzheimer Disease European Sequencing
ADSP: American Alzheimer Disease Sequencing Project
BIC: Bayesian Information Criterion
CSF: Cerebrospinal fluid
EM: Expectation-maximization
EOAD: Early-onset Alzheimer disease
GRS: Genetic risk score
MAF: Minor allele frequency
Mis3: Missense variant predicted damaging by three bioinformatical tools
OR: Odds ratio
PTV: Protein-truncating variant
QMPSF: Quantitative multiplex PCR of short fluorescent fragments
SNP: Single nucleotide polymorphisms
WES: Whole-exome sequencing
