# Publisher Correction: Penetrance estimation of Alzheimer disease in SORL1 loss-of-function variant carriers using a family-based strategy and stratification by APOE genotypes

**DOI:** 10.1186/s13073-022-01091-1

**Published:** 2022-08-03

**Authors:** Catherine Schramm, Camille Charbonnier, Aline Zaréa, Morgane Lacour, David Wallon, CNRMAJ collaborators, Anne Boland, Jean-François Deleuze, Robert Olaso, ADES consortium, Flora Alarcon, Dominique Campion, Grégory Nuel, Gaël Nicolas

**Affiliations:** 1 Normandie Université, UNIROUEN, Inserm U1245, CHU Rouen, Department of Genetics and CNRMAJ, FHU-G4 Génomique, 22 boulevard Gambetta – CS 76183, F-76000 Rouen, France; 2 Normandie Université, UNIROUEN, Inserm U1245, CHU Rouen, Department of Neurology and CNRMAJ, FHU-G4 Génomique, F-76000 Rouen, France; 3 Université Paris-Saclay, CEA, Centre National de Recherche en Génomique Humaine, 91057 Evry, France; 4 MAP5, UMR-CNRS 8145, Paris University, 75270 Paris, France; 5 Department of Research, Rouvray Psychiatric Hospital, 76681 Sotteville-Lès-Rouen, France; 6 LPSM, CNRS 8001, Sorbonne University, 75005 Paris, France

**Publisher Correction: Genome Med 14, 69 (2022)**

**https://doi.org/10.1186/s13073-022-01070-6**

Following publication of the original article [1], an error occurred during production process. It was identified in the lists of CNRMAJ collaborators and ADES consortium.

Collaborators are currently provided in Additional file 1.

They should be listed in the Acknowledgements section as below:

List of CNRMAJ (National Reference Center for Young Alzheimer Patients, Centre National de Référence Malades Alzheimer Jeunes) collaborators: 

firstname (midle initials), lastname, institution, city, country

Daniela, Andriuta, Amiens university Hospital, Amiens, France; Pierre, Anthony, Colmar Hospital, Colmar, France; Sophie, Auriacombe, Bordeaux university Hospital, Bordeaux, France; Anna-Chloé, Balageas, Tours university Hospital, Tours, France; Guillaume, Ballan, Bayonne Hospital, Bayonne, France; Mélanie, Barbay, Amiens university Hospital, Amiens, France; Emilie, Beaufils, Tours university Hospital, Tours, France; Yannick, Béjot, Dijon university Hospital, Dijon, France; Serge, Belliard, Rennes university Hospital, Rennes, France; Marie, Benaiteau, Toulouse university Hospital, Toulouse, France; Karim, Bennys, Montpellier university Hospital, Montpellier, France; Frédéric, Blanc, Strasbourg university Hospital, Strasbourg, France; Stéphanie, Bombois, Pitié, Salpêtrière university Hospital, Paris, France; Claire, Boutoleau Bretonnière, Nantes university Hospital, Nantes, France; Pierre, Branger, Caen university Hospital, Caen, France; Jasmine, Carlier, Toulouse university Hospital, Toulouse, France; Leslie, Cartz-Piver, Limoges university Hospital, Limoges, France; Pascaline, Cassagnaud, Lille university Hospital, Lille, France; Giovanni, Castelnovo, Nimes university Hospital, Nimes, France; Christine, Champion, Villeurbanne university Hospital, Villeurbanne, France; Annabelle, Chaussenot, Nice university Hospital, Nice, France; Mathieu, Ceccaldi, Marseille university Hospital, Marseille, France; Valérie, Chauviré, Angers university Hospital, Angers, France; Yaohua, Chen, Lille university Hospital, Lille, France; Julien, Cogez, Caen university Hospital, Caen, France; Emmanuel, Cognat, Lariboisière university Hospital, Paris, France; Fabienne, Contegal-Callier, Beaune Hospital, Beaune, France; Lea, Corneille, Marseille university Hospital, Marseille, France; Philippe, Couratier, Limoges university Hospital, Limoges, France; Hélène, Courtemanche, Nantes university Hospital, Nantes, France; Benjamin, Cretin, Strasbourg university Hospital, Strasbourg, France; Charlotte, Crinquette, Lille university Hospital, Lille, France; Bernard, Croisille, Lyon university Hospital, Lyon, France; Benjamin, Dauriat, Limoges university Hospital, Limoges, France; Sophie, Dautricourt, Caen university Hospital, Caen, France; Vincent, de la Sayette, Caen university Hospital, Caen, France; Astrid, De liège, Pitié Salpêtrière university Hospital, Paris, France; Marie, De Verdal, Arles Hospital, Arles, France; Didier, Deffond, Clermont-Ferrand university Hospital, Clermont-Ferrand, France; Benoit, Delpont, Dijon university Hospital, Dijon, France; Florence, Demurger, Rennes university Hospital, Rennes, France; Vincent, Deramecourt, Lille university Hospital, Lille, France; Céline, Derollez, Lille university Hospital, Lille, France; Mira, Didic, Marseille university Hospital, Marseille, France; Giulia, Diemert, Montpellier university Hospital, Montpellier, France; Elsa, Dionet, Clermont-Ferrand university Hospital, Clermont-Ferrand, France; Philippe, Diraison, Quimper Hospital, Quimper, France; Aude, Doan, Rouen university Hospital, Rouen, France; Martine, Doco Fenzy, Reims university Hospital, Reims, France; Boris, Dufournet, Marseille university Hospital, Marseille, France; Julien, Dumurgier, Lariboisière university Hospital, Paris, France; Hélène, Durand, Strasbourg university Hospital, Strasbourg, France; Anaïs, Dutray, Perpignan Hospital, Perpignan, France; Frédérique, Etcharry-Bouyx, Angers university Hospital, Angers, France; Maïté, Formaglio, Lyon university Hospital, Lyon, France; Audrey, Gabelle, Montpellier university Hospital, Montpellier, France; Anne, Gainche-Salmon, Rennes university Hospital, Rennes, France; Jean-Claude, Getenet, Saint-Etienne university Hospital, Saint-Etienne, France; Emmanuelle, Ginglinger, Mulhouse university Hospital, Mulhouse, France; Olivier, Godefroy, Amiens university Hospital, Amiens, France; Mathilde, Graber, Dijon university Hospital, Dijon, France; Chloé, Gregoire, Bordeaux university Hospital, Bordeaux, France; Stephan, Grimaldi, Marseille university Hospital, Marseille, France; Julien, Gueniat, Dijon university Hospital, Dijon, France; Claude, Gueriot, Marseille university Hospital, Marseille, France; Sophie, Haffen, Besançon university Hospital, Besançon, France; Lorraine, Hamelin, Sainte-Anne university Hospital, Paris, France; Didier, Hannequin, Rouen university Hospital, Rouen, France; Cezara, Hanta, Rennes university Hospital, Rennes, France; Clémence, Hardy, Rouen university Hospital, Rouen, France; Geoffroy, Hautecloque, Colmar Hospital, Colmar, France; Camille, Heitz, Nimes university Hospital, Nimes, France; Claire, Hourregue, Lariboisière university Hospital, Paris, France; Thérèse, Jonveaux, Nancy university Hospital, Nancy, France; Snejana, Jurici, Perpignan Hospital, Perpignan, France; Catia, Khoumri, Dijon university Hospital, Dijon, France; Lejla, Koric, Marseille university Hospital, Marseille, France; Pierre, Krolak-Salmon, Villeurbanne Hospital, Villeurbanne, France; Pierre, Labauge, Montpellier university Hospital, Montpellier, France; Morgane, Lacour, Rouen university Hospital, Rouen, France; Julien, Lagarde, Sainte-Anne university Hospital, Paris, France; Hélène-Marie, Lanoiselée, Orléans Hospital, Orléans, France; Brice, Laurens, Bordeaux university Hospital, Bordeaux, France; Isabelle, Le Ber, Pitié Salpêtrière university Hospital, Paris, France; Gwenaël, Le Guyader, Poitiers university Hospital, Poitiers, France; Amélie, Leblanc, Brest university Hospital, Brest, France; Thibaud, Lebouvier, Lille university Hospital, Lille, France; Anaïs, Lippi, Montpellier university Hospital, Montpellier, France; Marie-Anne, Mackowiak, Lille university Hospital, Lille, France; Eloi, Magnin, Besançon university Hospital, Besançon, France; Cecilia, Marelli, Montpellier university Hospital, Montpellier, France; Olivier, Martinaud, Caen university Hospital, Caen, France; Aurélien, Maureille, Lille university Hospital, Lille, France; Emilie, Milongo-Rigal, Toulouse university Hospital, Toulouse, France; Sophie, Mohr, Dijon university Hospital, Dijon, France; Hélène, Mollion, Lyon university Hospital, Lyon, France; Olivier, Moreaud, Grenoble university Hospital, Grenoble, France; Alexandre, Morin, Rouen university Hospital, Rouen, France; Gaël, Nicolas, Rouen university Hospital, Rouen, France; Julia, Nivelle, Ales Hospital, Ales, France; Camille, Noiray, Sainte-Anne university Hospital, Paris, France; Elisabeth, Ollagnon-Roman, Lyon university Hospital, Lyon, France; Claire, Paquet, Lariboisière university Hospital, Paris, France; Jérémie, Pariente, Toulouse university Hospital, Toulouse, France; Florence, Pasquier, Lille university Hospital, Lille, France; Alexandre, Perron, Amiens university Hospital, Amiens, France; Nathalie, Philippi, Strasbourg university Hospital, Strasbourg, France; Virginie, Pichon, Angers university Hospital, Angers, France; Vincent, Planche, Bordeaux university Hospital, Bordeaux, France; Céline, Poirsier, Reims university Hospital, Reims, France; Marie, Rafiq, Toulouse university Hospital, Toulouse, France; Pauline, Rod-Olivieri, Sainte-Anne university Hospital, Paris, France; Adeline, Rollin-Sillaire, Lille university Hospital, Lille, France; Carole, Roué-Jagot, Sainte-Anne university Hospital, Paris, France; Dario, Saracino, Pitié-Salpêtrière university Hospital, Paris, France; Marie, Sarazin, Sainte-Anne university Hospital, Paris, France; Mathilde, Sauvée, Grenoble university Hospital, Grenoble, France; François, Sellal, Colmar Hospital, Colmar, France; Lila, Sirven Villaros, Nimes university Hospital, Nimes, France; Christel, Thauvin, Dijon university Hospital, Dijon, France; Camille, Tisserand, Toulouse university Hospital, Toulouse, France; Christophe, Tomasino, Orléans Hospital, Orléans, France; Cédric, Turpinat, Montpellier university Hospital, Montpellier, France; Laurène, Van Damme, Perpignan university Hospital, Perpignan, France; Olivier, Vercruysse, Saint-Brieuc Hospital, Saint-Brieuc, France; Alice, Voilly, Besançon university Hospital, Besançon, France; Nathalie, Wagemann, Nantes university Hospital, Nantes, France; David, Wallon, Rouen university Hospital, Rouen, France; Aline, Zarea, Rouen university Hospital, Rouen, France.

List of ADES (Alzheimer Disease European Sequencing project) collaborators:

firstname (midle initials), lastname, institution, city, country

Shahzad, Ahmad, Erasmus Medical Centre, Rotterdam, The Netherlands; Najaf, Amin, Erasmus Medical Centre, Rotterdam, The Netherlands; Philippe, Amouyel, Lille university Hospital, Lille, France; Céline, Bellenguez, Pasteur Institute, Lille, France; Claudine, Berr, Montpellier university, Montpellier, France; Anne, Boland, Centre National de Recherche en Génomique Humaine, Evry, France; Paola, Bossù, IRCCS Santa Lucia Foundation, Rome, Italy; Femke, Bouwman, Vrije University, Amsterdam, The Netherlands; Jose, Bras, Van Andel Institute, Grand Rapids, MI USA; Dominique, Campion, Rouen university Hospital, Rouen, France; Camille, Charbonnier, Rouen university Hospital, Rouen, France; Jordi, Clarimon, Universitat Autònoma de Barcelona, Barcelona, Spain; Antonio, Daniele, Catholic university of Sacred Heart, Rome, Italy; Jean-François, Dartigues, Bordeaux Population Health Research Center, Bordeaux, France; Stéphanie, Debette, Bordeaux Population Health Research Center, Bordeaux, France; Jean-François, Deleuze, Centre National de Recherche en Génomique Humaine, Evry, France; Nicola, Denning, Cardiff university, Cardiff, UK; Oriol, Dols-Icardo, Universitat Autònoma de Barcelona, Barcelona, Spain; Nick C., Fox, UCL Queen Square Institute of Neurology, London, UK; Daniela, Galimberti, University of Milan, Milan, Italy; Emmanuelle, Génin, Brest University, Brest, France; Hans, Gille, Vrije University, Amsterdam, The Netherlands; Benjamin, Grenier-Boley, Pasteur Institute, Lille, France; Detelina, Grozeva, Cardiff University, Cardiff, UK; Rita, Guerreiro, Van Andel Institute, Grand Rapids, MI USA; John J., Hardy, UCL Institute of Neurology, London, UK; Clive, Holmes, University of Southampton, Southampton, UK; Henne, Holstege, Amsterdam university Hospital, Amsterdam, The Netherlands; Marc, Hulsman, Amsterdam university Hospital, Amsterdam, The Netherlands; Holger, Hummerich, UCL Institute of Prion Diseases, London, UK; M. Arfan, Ikram, Erasmus Medical Centre, Rotterdam, The Netherlands; M. Kamran, Ikram, Erasmus Medical Centre, Rotterdam, The Netherlands; Iris, Jansen, Vrije University, Amsterdam, The Netherlands; Amit, Kawalia, University of Cologne, Cologne, Germany; Robert, Kraaij, Erasmus Medical Centre, Rotterdam, The Netherlands; Jean-Charles, Lambert, Pasteur Institute, Lille, France; Marc, Lathrop, McGill University, Montreal, QC Canada; Afina W., Lemstra, Vrije University, Amsterdam, The Netherlands; Alberto, Lleó, Universitat Autònoma de Barcelona, Barcelona, Spain; Lauren, Luckcuck, Cardiff University, Cardiff, UK; Marcel M.A.M., Mannens, University of Amsterdam, The Netherlands; Rachel, Marshall, Cardiff University, Cardiff, UK; Carlo, Masullo, Catholic University of the Sacred Heart, Rome, Italy; Simon, Mead, UCL Institute of Prion Diseases, London, UK; Patrizia, Mecocci, University of Perugia, Perugia, Italy; Alun, Meggy, Cardiff university, Cardiff, UK; Merel O., Mol, Erasmus Medical Centre, Rotterdam, The Netherlands; Kevin, Morgan, University of Nottingham, Nottingham, UK; Benedetta, Nacmias, University of Florence, Florence, Italy; Gaël, Nicolas, Rouen university Hospital, Rouen, France; Penny J., Norsworthy, UCL Institute of Prion Diseases, London, UK; Florence, Pasquier, Lille university Hospital, Lille, France; Pau, Pastor, Barcelona university Hospital, Barcelona, Spain; Olivier, Quenez, Rouen university Hospital, Rouen, France; Alfredo, Ramirez, University of Cologne, Cologne, Germany; Rachel, Raybould, Cardiff university, Cardiff, UK; Richard, Redon, Nantes university Hospital, Nantes, France; Marcel, J.T., Reinders, Delft university of Technology, Delft, The Netherlands; Anne-Claire, Richard, Rouen university Hospital, Rouen, France; Steffi G., Riedel-Heller, University of Leipzig, Leipzig, Germany; Fernando, Rivadeneira, Erasmus Medical Centre, Rotterdam, The Netherlands; Stéphane, Rousseau, Rouen university Hospital, Rouen, France; Natalie S., Ryan, UCL Queen Square Institute of Neurology, London, UK; Salha, Saad, Cardiff University, Cardiff, UK; Pascual, Sanchez-Juan, National Institute of Health Carlos III, Madrid, Spain; Philip, Scheltens, Vrije University, Amsterdam, The Netherlands; Jonathan M., Schott, UCL Queen Square Institute of Neurology, London, UK; Davide, Seripa, Laboratory for Advanced Hematological Diagnostics, Lecce, Italy; Daoud, Sie, Vrije University, Amsterdam, The Netherlands; Rebecca, Sims, Cardiff University, Cardiff, UK; Erik, Sistermans, Vrije University, Amsterdam, The Netherlands; Sandro, Sorbi, University of Florence, Florence, Italy; Resie, van Spaendonk, Vrije University, Amsterdam, The Netherlands; Gianfranco, Spalleta, IRCCS Santa Lucia Foundation, Rome, Italy; Niccòlo, Tesi, Delft University of Technology, Delft, The Netherlands; Betty, Tijms, Vrije University, Amsterdam, The Netherlands; André G., Uitterlinden, Erasmus Medical Centre, Rotterdam, The Netherlands; Wiesje M., van der Flier, Vrije University, Amsterdam, The Netherlands; Sven J., van der Lee, Delft, University of Technology, Delft, The Netherlands; Cornelia M., van Duijn, Erasmus Medical Centre, Rotterdam, The Netherlands; Jeroen G.J., van Rooij, Erasmus Medical Centre, Rotterdam, The Netherlands; John C., van Swieten, Erasmus Medical Centre, Rotterdam, The Netherlands; Pieter J., de Visser, Vrije University, Amsterdam, The Netherlands; Michael, Wagner, University of Bonn, Bonn, Germany; David, Wallon, Rouen university Hospital, Rouen, France; Julie, Williams, Cardiff University, Cardiff, UK; Aline, Zarea, Rouen university Hospital, Rouen, France; Alzheimer’s Disease Neuroimaging Initiative (ADNI) database; Alzheimer Disease Sequencing Project (ADSP).

The publisher apologies for any inconvenience this error may have caused.

The original article [1] has been corrected.
