# Supplementary material for: Penetrance estimation of Alzheimer disease in SORL1 loss-of-function variant carriers using a family-based strategy and stratification by APOE genotypes
Source: Genome Med. 2022 Jun 28;14:69. doi: 10.1186/s13073-022-01070-6 (PMC9238165; doi:10.1186/s13073-022-01070-6)
Supplement: Supplementary file 1 — Additional file 1: Supplementary methods. Statistical modeling, simulation study. Supplementary results. Comparison of bootstrap and jackknife procedures, results from the simulation study, pedigrees information. Supplementary discussion about the model. Fig. S1. Number of informative phenotypes available in maternal versus paternal branch over the 27 LoF families. Fig. S2. Age distribution in LoF families. Fig. S3. Results of simulations in scenario A when missingness does not depend on age. Fig. S4. Results of simulation in scenario 1 when missingness depends on age. Fig. S5. Results of simulation in scenario B. Fig. S6. Results of simulation in scenario B when missingness is related to one family branch only (the one with the lower number of cases). Fig. S7. Results of simulation in scenario B when missingness is related to one family branch only (the one with parent not being a case). Fig. S8. Results of simulation in scenario C with normal random effect. Fig. S9. Results of simulation in scenario C with gamma random effect. Fig. S10. Results of simulations in scenario D. Fig. S11. Posterior probability of carrying a variant in the baseline scenario over 500 simulated datasets. Fig. S12. Distribution of parameters obtained over 500 iterations of bootstrap and jackknife methods. Fig. S13. Age dependent penetrance for carriers and non-carriers of a SORL1 PTV. Fig. S14. Distribution of AAO for SORL1-LoF carriers being AD cases in the case-control dataset according to their APOE genotype. Table S1.SORL1 variants included in our family cohort. Table S2. Comparison of models based on the Bayesian Information Criterion (BIC). Table S3. Expected total number of individuals by genotype. List of CNRMAJ collaborators. List of ADES collaborators. [file 13073_2022_1070_MOESM1_ESM.pdf]

Supplementary methods

Statistical modeling

Notations

Let n be the total number of subjects. Let Y_i be the age at disease onset for subject i and C_i the censoring time such that $T_i = \min(Y_i, C_i)$ is the observed time associated with the status δ_i equaling 1 if $T_i = Y_i$ and 0 otherwise. In other words, δ_i indicates if the subject i has developed AD at age T_i.

We want to estimate the penetrance function $F(t \mid a, s)$, which gives the probability that an individual develops the disease before age t given their *APOE* status a and *SORL1* status s:

$$F(t \mid a, s) = \mathbb{P}(Y_i \leq t | APOE = a, SORL1 = s)$$

As usually done in survival analysis, the penetrance function is modeled through the survival function $S(t \mid a, s)$ which models the time to disease onset:

$$S(t \mid a, s) = 1 - F(t \mid a, s) = \exp\left(-\Lambda(t \mid a, s)\right) = \exp\left(-\int_0^t \lambda(u \mid a, s)du\right)$$

where $\lambda(t \mid a, s)$ denotes the hazard function corresponding to the instantaneous risk of developing AD at time t conditional on not having developed it before for individuals of *APOE* status a and *SORL1* status s; and similarly, $\Lambda(t \mid a, s)$ denotes the cumulative risk until time t for these individuals.
This hazard function takes the following parametric form:

$$\lambda(t \mid a, s) = \begin{cases} \lambda_{\text{nc}}(t \mid a) & \text{for non carriers of } \textit{SORL1} \text{ LoF variants} \\ \lambda_{\text{c}}(t \mid a) = \lambda_{\text{nc}}(t \mid a)\exp(\beta(t)) & \text{for carriers of } \textit{SORL1} \text{ LoF variants} \end{cases}$$

Estimating $\lambda_{\text{nc}}(t \mid a)$, the piecewise constant hazard model for the baseline age-related penetrance adjusted on *APOE* genotype

The non carrier hazard rate $\lambda_{\text{nc}}(t \mid a)$ is a piecewise constant function designed to match exactly the *APOE*-specific penetrance estimates derived from age 65 to age 100 in the general population by van der Lee et al.[1] (see supplementary Table 3 of van der Lee et al.[1]). We estimated parameters for $\lambda_{\text{nc}}(t \mid a)$ such that $F_{\text{nc}}(t \mid a)$ matches with $P(t \mid a)$, the penetrance estimated every 5 years from 65 years-old up to 95 years-old by the original paper.
Published data did not include case with disease onset before 60 years old, suggesting that $\forall t \leq 60,\ P(t \mid a) = 0$. To account for the accumulation of risk before 65 years-old, in carriers of *SORL1* LoF variant, we propose to cut

time into the following intervals $\mathcal{I} = \Big\{]40;65],\ ]65;70],\ ]70;75],\ ]75;80],\ ]80;85],\ ]85;90],\ ]90;95],\ > 95 \Big\}$ with:

$$\lambda_{\text{nc}}(t \mid a) = \begin{cases} 0 & \text{if } t \in [0;40] \\ \exp(\alpha_{a,k}) \text{ with } \alpha_{a,k} \sim \mathcal{N}(\mu_{a,k}, \sigma^2_{a,k}) & \text{if } t \text{ is in the } k^{th} \text{ interval of } \mathcal{I}\text{: }]t_{k-1};t_k] \end{cases}$$

such that $F_{\text{nc}}(t \mid a)$ is not null on $]40, 65]$. Instead of estimating $\alpha_{a,k}$ from published mean penetrance estimation, we propose to estimate $\mu_{a,k}$ and $\sigma_{a,k}$ from published confidence intervals in order to integrate uncertainty linked to *APOE* into our estimations.

For each *APOE* genotype a and $\forall k$, $\mu_{a,k}$ and $\sigma_{a,k}$ are estimated by:

$$\mu_{a,k} = \frac{\alpha^{(L)}_{a,k} + \alpha^{(U)}_{a,k}}{2} \qquad \text{and} \qquad \sigma_{a,k} = \frac{\alpha^{(U)}_{a,k} - \alpha^{(L)}_{a,k}}{2 \times 1.96}$$

where $\alpha^{(L)}_{a,k}$ (respectively $\alpha^{(U)}_{a,k}$) was deduced from $P^{(L)}(t,a)$ (respectively $P^{(U)}(t,a)$), the lower (respectively upper) bound of the 95% confidence interval of published penetrance curve as follow:

$$\exp(\alpha^{(L)}_{a,k}) = \frac{-1}{t_k - t_{k-1}} \times \log\left(\frac{1 - P^{(L)}(t_k \mid a)}{1 - P^{(L)}(t_{k-1} \mid a)}\right) \qquad \exp(\alpha^{(U)}_{a,k}) = \frac{-1}{t_k - t_{k-1}} \times \log\left(\frac{1 - P^{(U)}(t_k \mid, a)}{1 - P^{(U)}(t_{k-1} \mid a)}\right)$$

Of notes, estimates displayed in supplementary Table 3 of van der Lee et al.[1] are rounded to 0 for $P^{(L)}(t = 65 \mid a = \varepsilon3\varepsilon3)$ and for $P^{(L)}(t = 65 \mid a = \text{heterozygous } \varepsilon4)$. To overcome this problem, we proposed to approximate $P^{(L)}(t = 65 \mid a)$ by:

$$1 - \exp\left(-(65-40) \times \exp\left(2 \times \log\left(-\frac{\log(1 - P(t = 65 \mid a))}{65 - 40}\right) - \log\left(-\frac{\log(1 - P^{(U)}(t = 65 \mid a))}{65 - 40}\right)\right)\right)$$

for $a = \varepsilon3\varepsilon3$ and $a =$ heterozygous $\varepsilon4$ respectively.

Finally, to compensate for the rarity of *APOE* ε2, *APOE* genotypes are modeled through the number of *APOE* ε4 alleles and therefore averaged into three categories: no allele ε4, heterozygous ε4 and ε4ε4 individuals. Of notes, published estimates for non-carriers of ε4 allele are split between ε3ε3 on the one hand and individuals carrying genotypes ε2ε2 or ε2ε3 on the other hand. We recomputed $P(t, \text{no allele } \varepsilon4)$ using the following formula:

$$\forall t \in \mathbb{R}^+,\ P(t \mid \text{no allele } \varepsilon4) = \frac{p_{33} \times P(t \mid \varepsilon3\varepsilon3) + p_{22+23} \times P(t \mid \varepsilon2\varepsilon2 \text{ or } \varepsilon2\varepsilon3)}{p_{33} + p_{22+23}}$$

where p_{33} and p_{22+23} are respectively the proportion of *APOE*-ε3ε3 and *APOE*-(ε2ε2 + ε2ε3) genotypes in the data set.

Correction for ascertainment bias

To tackle the important issue of ascertainment bias resulting from the recruitment of early onset carriers of *SORL1* LoF variants as probands, we took their genotype information into account at each E-step but discarded proband phenotype information (observed time, disease status) at each M-step as in Alarcon et al.[2]. Moreover, since only few individuals' observation time were greater that 85 years of age, we censored data at 85 years-old.

Model choice

To validate the choice of $\{\tau_0, ..., \tau_{J-1}\}$ for the piecewise modeling of the effect of *SORL1* LoF variants $\beta(t)$, several cut-offs were envisaged and the different models were compared based on a Bayesian Information Criterion (BIC) measure defined as:

$$\text{BIC} = -2 \times \log(\mathcal{L}) + k \times \log(d)$$

where $\log(\mathcal{L})$ is the log-likelihood of the model returned by `bped` computed using the sum-product algorithm from the evidence probability over all possible genotypes, k is the number of parameters and d the number of subjects who experienced the event of disease onset. The model with the best fit is the one with the lowest BIC.

Computation of confidence intervals

Confidence intervals (CI) were computed using a bootstrap strategy of 500 iterations. At each iteration, families were randomly selected with replacement and model parameters were re-estimated on these observations (same family number) to obtain the associated age-related penetrance curves. Then, for each age, we took the 2.5^{th} and 97.5^{th} quantiles as the boundaries of the 95%CI. Uncertainty surrounding *APOE*-adjusted baseline survival curves as reflected by their confidence intervals was taken into account in the bootstrap procedure.

Uncertainty surrounding *APOE* effect. Confidence intervals for non carriers of *SORL1* variant are re-computed from parameters $\mu_{a,k}$ and $\sigma_{a,k}$ defined in section of supplemental information using the following procedure:

- Repeat 500 times the steps 1 to 4:

  1. Generate $u \sim \mathcal{N}(0, 1)$
  2. $\forall k, \forall a$, compute $\alpha_{a,k} = \mu_{a,k} + \sigma_{a,k} \times u$
  3. Define the function $\lambda_{\text{nc}}(t \mid a)$ from $\alpha_{a,k}$ as in section of supplemental information
  4. Compute the penetrance $F_{\text{nc}}(t \mid a) = 1 - \exp\left(-\int_0^t \lambda_{\text{nc}}(u \mid a) du\right)$

- We determined the pointwise 95%CI as the 2.5^{th} and 97.5^{th} quantiles at each age t over the 500 estimations.

Uncertainty surrounding both *SORL1* and *APOE* effects. Confidence intervals for carriers of *SORL1* variant are computed combing a bootstrap strategy of 500 iterations and the strategy described above, by using the following procedure:

• Repeat 500 times the steps 1 to 6:

1. Randomly select families with replacement (same number of families as in the whole dataset)
2. Generate $u \sim \mathcal{N}(0,1)$
3. $\forall k, \forall a$, compute $\alpha_{a,k} = \mu_{a,k} + \sigma_{a,k} \times u$
4. Define the function $\lambda_{\text{nc}}(t \mid a)$ from $\alpha_{a,k}$ as in section of supplemental information
5. Compute $\beta(t)$ using EM algorithm incorporating the current dataset and the current $\lambda_{\text{nc}}(t \mid a)$ function
6. Compute the penetrance $F_{\text{c}}(t \mid a) = 1 - \exp\left(-\int_0^t \lambda_{\text{nc}}(u \mid a) \exp(\beta(t))\, du\right)$

• We determined the pointwise 95%CI as the 2.5^{th} and 97.5^{th} quantiles at each age t over the 500 estimations.

We also performed 500 iterations of a Jackknife analysis removing randomly 10% of known genotype among relatives to assess the reliability of our estimations. Similarly to bootstrap procedure, the Jackknife analysis takes into account variability linked to *APOE*.

Simulation study

We propose to challenge our model in a simulation study. For each *scenario*, we generated 500 independent datasets and apply the model described in the main manuscript on each of them. Data were generated using the following steps:

- Initialisation step:

  Let $N = 27$ the number of families to include in the cohort.

  $n \leftarrow 0$.

- While $n < N$, do:
  - Generate a pedigree structure ($\mathcal{P}^\star$)
  - Generate genotypes by randomly attributing founders *APOE* genotype based on allele frequencies computed from the 11,375 individuals included in van der Lee et al.[1] (allele frequency for ε2: 0.085; ε3: 0.764; ε4: 0.151). The *SORL1* variant has been randomly attributed to one of the founder. Then, genotype of non-founders were iteratively deduced from their parents with an allele transmission probability of 1/2.

- Generate phenotypes

  Generate age at AD onset Y according to the hazard function $\lambda^{\star}(t)$

  Generate censoring time $C \sim \mathcal{N}(65, 15^2)$

  Let $T = \min(Y, C)$, the observed time and $\delta = \mathbb{1}_{Y \leq C}$ the disease status

- Select proband and its family

  If the third generation of the family includes an affected individual with AD onset before 65 years and carrier of a *SORL1* variant, then this individual is defined as the proband, the family is included in the cohort and $n \leftarrow n + 1$.

- Determine available data in terms of phenotype ($\varphi^{\star}$) and genotype ($\Theta^{\star}$). Of note, proband's phenotype and genotype are always known.

We detailed below the choice of $\mathcal{P}^{\star}$, $\lambda^{\star}(t)$, $\phi^{\star}$ and $\Theta^{\star}$ for the baseline *scenario* and how they were adapted for *scenarii* A, B, and C assessing respectively the impact of the proportion of missing genotypes, the proportion of missing phenotypes and the addition of a familial effect on the estimation, as well as for *scenario* D modifying the number of cut points for the piecewise constant function $\beta(t)$ to assess the performance of the BIC in the choice of the model.

The baseline scenario

The pedigree structure ($\mathcal{P}^{\star}$)

To be congruent with number of available data in our cohort of families with LoF variants, we simulated pedigree of 12 individuals over three generations: four grand-parents (founders, generation 1), maternal (respectively paternal) branch including the mother (respectively father) and a uncle/aunt (generation 2) and the proband with three siblings (generation 3). Considering the censoring distribution, this lead to a mean of 11 informative individuals (age $\geq$ 40) by family (see Table 1 and Additional file 1: Fig. S2).

The model ($\lambda^{\star}(t)$)

Age at AD onset T was generated using the inverse transformation method, according to the following model:

$$\lambda^{\star}(t \mid x, z) = \lambda_{\text{nc}}(t \mid x) \exp(\beta(t) \mathbb{1}_{z=1})$$

where x is the number of ε4 allele and z the indicator of the presence of a *SORL1* variant of interest. We supposed three hypotheses for variants effect $\beta(t)$:

- Hypothesis 0: $\beta(t) = 0 \quad \forall t \in \mathbb{R}^{*+}$

- Hypothesis 1: $$\beta(t) = \begin{cases} 3.46 & \text{if } 0 < t \leq 60 \\ 6.67 & \text{if } 60 < t \leq 65 \\ 4.68 & \text{if } 65 < t \leq 70 \\ 3.25 & \text{if } 70 < t \end{cases}$$
- Hypothesis 2: $$\beta(t) = \begin{cases} 1.78 & \text{if } 0 < t \leq 60 \\ 3.34 & \text{if } 60 < t \leq 65 \\ 2.34 & \text{if } 65 < t \leq 70 \\ 1.63 & \text{if } 70 < t \end{cases}$$

Under hypothesis 0, we suppose that the *SORL1* variant has no effect on AD whereas under hypothesis 1, we suppose that the effect is similar to the one we computed through the real data. Finally, we evaluateD the model under a more moderate effect of *SORL1* variants (hypothesis 2).

Available data for the analysis ($\phi^\star$ and $\Theta^\star$)

We suppose that the phenotype is known for all individuals in the cohort.
We suppose that the genotype is known for the proband and for 10% and 20% of its relatives of generation 2 and 3 with $Y \geq 70y$ and $Y < 70y$ respectively, which reflect the reality of our cohort. We suppose that genotype is not available for grand-parents (generation 1).

Scenario A: assessing the impact of missing genotypes on $\beta(t)$ estimation

Elements $\mathcal{P}^\star$, $\lambda^\star(t)$ and $\phi^\star$ are unchanged compared with the baseline *scenario*. The proportion of available genotypes varies according to the following possibilities:

- 100% available
- 100% missing (except the proband)
- available for the proband and for 10% of its relatives of generations 2 and 3
- available for the proband and for 50% of its relatives of generations 2 and 3
- available for the proband and for 0% and 10% of its relatives of generations 2 and 3 with respectively $Y \geq 70y$ and $Y < 70y$
- 0% and 30% of its relatives of generations 2 and 3 with respectively $Y \geq 70y$ and $Y < 70y$

Scenario B: assessing the impact of missing phenotypes on $\beta(t)$ estimation

Elements $\lambda^\star(t)$ and $\Theta^\star$ are unchanged compared with the baseline *scenario*. The proportion of available phenotypes for proband's relatives varies according to the following possibilities:

- 10% of missingness
- 50% of missingness
- 100% of missingness in one of the paternal or maternal branch (randomly selected)
- 50% of missingness in the parental branch with the less affected cases (both branches were kept if ex aequo)/parent without disease
- 100% of missingness in the parental branch with the less affected cases (both branches were kept if ex aequo)/parent without disease

Since small families may be less impacted by unbalanced missingness between maternal and paternal branches, this *scenario* was assessed for a larger pedigree structure adding three uncles/aunts in both parental branches for a total of 18 individuals.

Scenario C: assessing the impact of an additional family effect

Elements $\mathcal{P}^\star$, $\phi^\star$ and $\Theta^\star$ are unchanged compared with the baseline *scenario*. Here we propose to modify the model for generating T according to a new definitions of $\lambda^\star(t \mid x, z)$ including an additional family effect :

$$\lambda^\star(t \mid x, z) = \lambda_{\text{nc}}(t \mid x) \exp(\beta(t)\mathbb{1}_{z=1}) \times \nu$$

where ν is defined according to the following possibilities:

- $\nu = \exp(u\mathbb{1}_{z=1})$ with $u \sim \mathcal{N}(0, \sigma^2)$, $\sigma = 0.3$
- $\nu = \exp(u\mathbb{1}_{z=1})$ with $u \sim \mathcal{N}(0, \sigma^2)$, $\sigma = 0.5$
- $\nu = \exp(u\mathbb{1}_{z=1})$ with $u \sim \mathcal{N}(0, \sigma^2)$, $\sigma = 1$

} additional effect only affects carriers of the *SORL1* variant of interest

- $\nu \sim \Gamma(1, \theta)$, $\theta = 10$
- $\nu \sim \Gamma(1, \theta)$, $\theta = 1$

} additional effect affects similarly the whole family

Of notes, the baseline *scenario* corresponds to the cases $\sigma = 0$ or $\frac{1}{\theta} \to 0$.

For all *scenarii* above, data were generated under hypotheses 0, 1 and 2 and then analysed using the EM algorithm presented in the main article, for 3 cut-offs (60, 65, 70), when excluding and when including probands' phenotypes.

Scenario D: assessing the pertinence of BIC for the choice of the cut-points

Elements for scenario D are unchanged compared to the baseline scenario. Besides hypothesis 1, data were generated under two additional hypotheses including a lower number of cut-points:

- Hypothesis 3: $\beta(t) = 4.08 \quad \forall t \in \mathbb{R}^{*+}$

- Hypothesis 4: $$\beta(t) = \begin{cases} 4.74 & \text{if } 0 < t \leq 70 \\ 3.22 & \text{if } 70 < t \end{cases}$$

Then, data were analysed using the EM algorithm presented in this article when excluding probands. Several cut-points were tested and BIC was computed for each of them.

Supplementary results

Comparison of bootstrap and Jackknife procedures

Additional file 1: Fig. S12 displays the distribution of $\beta(t)$ components for both bootstrap and Jackknife procedures. The distributions are similar for younger age but for the older age, especially for $t \geq 70$, bootstrap distribution is larger. It may be explained be the fact that the bootstrap method selects families with replacement leading to a mean of unique families equaling 18 by iteration and ranging from 13 to 23 (among a total of 27 families). To be conservative, we displayed confidence intervals obtained by bootstrap method that can be considered as reliable.

Results from the simulation study

Scenario A:

In all *scenarii*, the inclusion of the proband in the analyses lead to a positive bias in $\beta(t)$ estimation before 65 years old. In constrast, the exclusion of the proband corrected for this bias. Results suggested that proportion of available genotype did not impact the bias, but a higher proportion of already known genotype help to reduce the uncertainty of our estimation (see Additional file 1: Fig. S3 and S4).

Scenario B:

In all *scenarii*, missing phenotype does not impact the results, even in unbalanced family design (see Additional file 1: Fig. S5, S6 and S7).

Scenario C:

When adding a random term associated with an heterogeneous variant effect across families or a family effect, the model may lead to an overestimation of $\beta(t)$ even if proband was excluded from the analysis. Besides the non consideration of a random term in the model, the ascertainment based on the age of the proband lead to the inclusion of families whose associated random terms are biased towards higher effect (see Additional file 1: Fig. S8 and S9).

Scenario D:

Whatever the number of cut-offs we considered, over the 500 simulated datasets, the median of BIC was always lower for the model including the right cut-offs (Additional file 1: Fig. S10). In the datasets generated from

model without cut-off ($\beta(t)$ contant over time), the BIC increased with number of cut-offs but the low pairwise difference in BIC suggests that an higher number of cut-offs is not deleterious for the estimation. In contrast, as expected,in the datasets generated from model including cut-offs, simulation results suggested that do not include the original cut-off(s) in the estimation lead to a higher BIC. Thus, BIC is an acceptable criterion for the choice of our model.

Genotyping probabilities

From the baseline scenario (under hypothesis 1), we showed that the sum of posterior genotyping probabilities may correctly estimate the proportion of carriers of the variant of interest (Additional file 1: Fig. S11).

Pedigrees information

This subsection displays the pedigree information of all families included in our study. Affected are in black, unaffected are in white. Age in years (y) corresponds to age of onset for affected individuals and age of censoring for unaffected individuals. The red arrow indicates the proband.

ALZ−0167

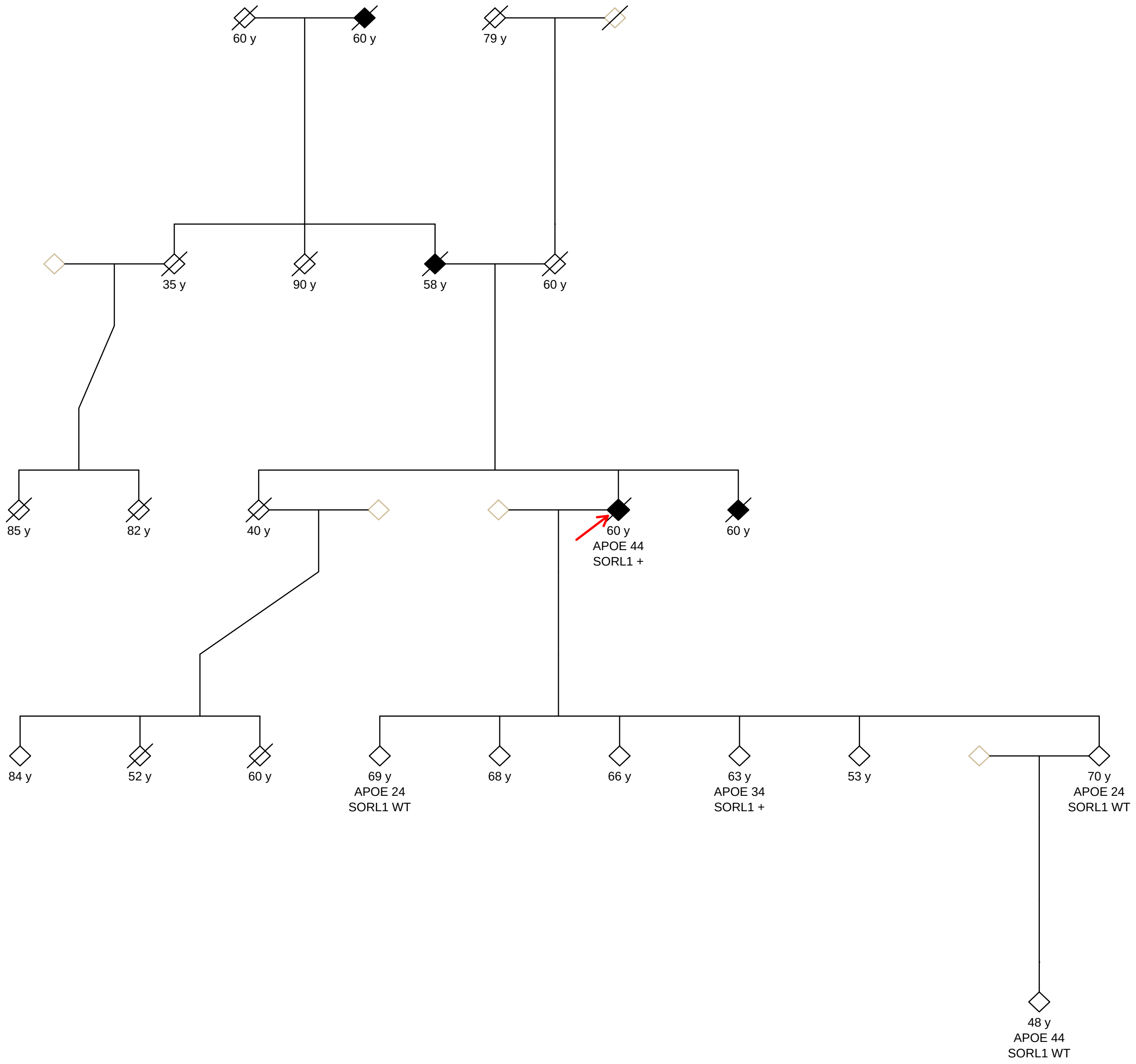
60 y
60 y
79 y
35 y
90 y
58 y
60 y
85 y
82 y
40 y
60 y
APOE 44
SORL1 +
60 y
84 y
52 y
60 y
69 y
APOE 24
SORL1 WT
68 y
66 y
63 y
APOE 34
SORL1 +
53 y
70 y
APOE 24
SORL1 WT
48 y
APOE 44
SORL1 WT

EFA−0198

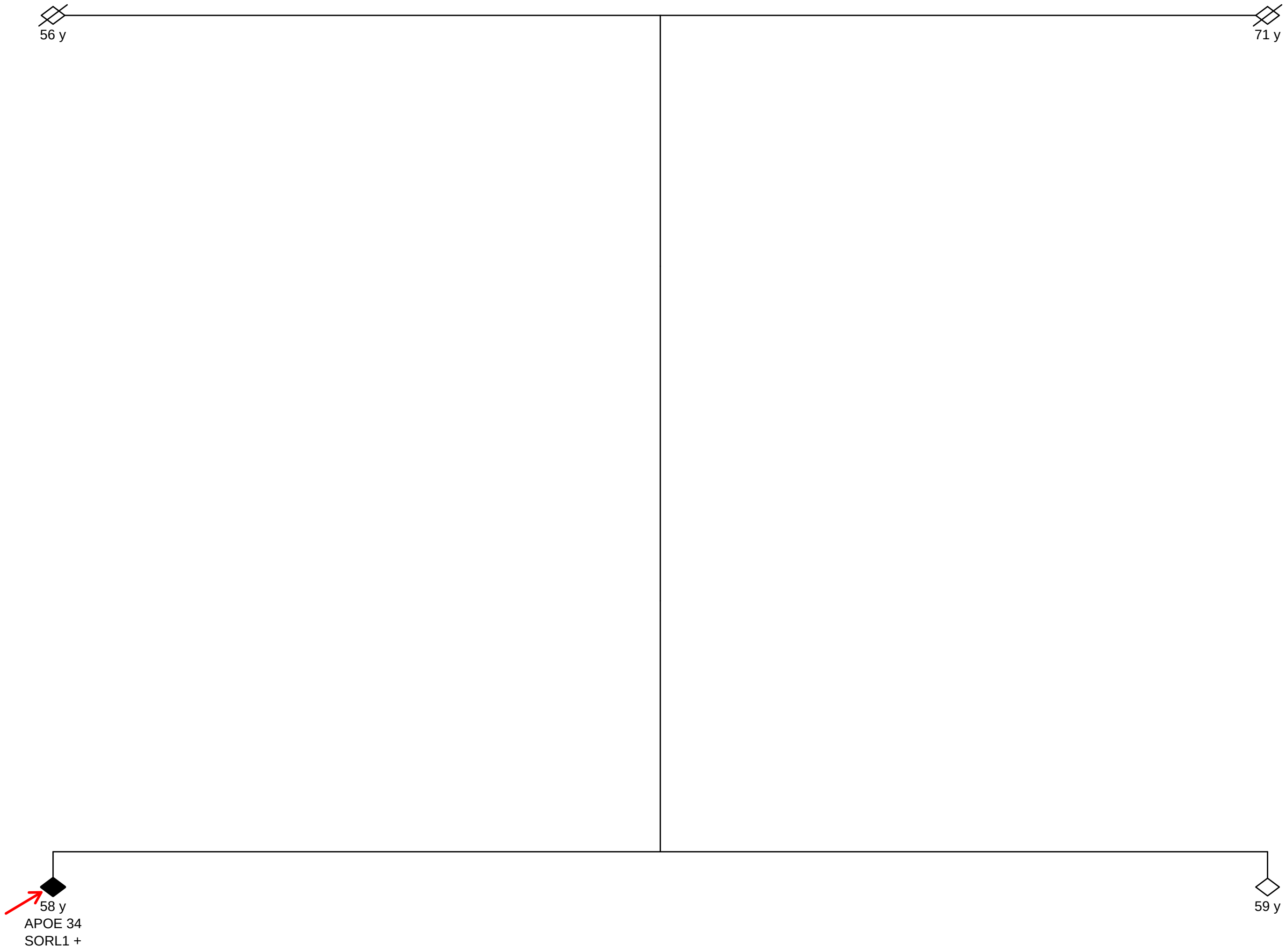

EFA−0085

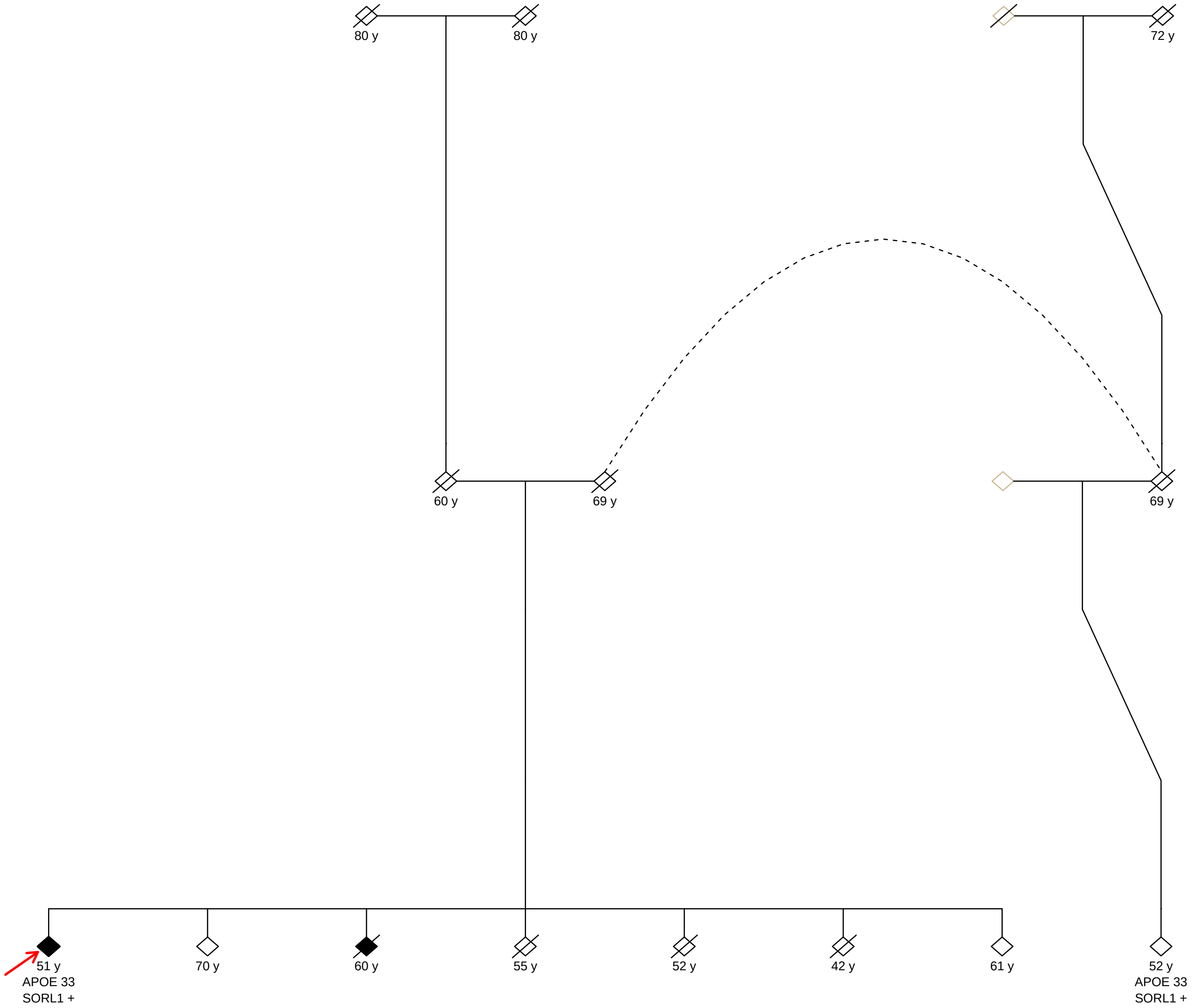
80 y
80 y
72 y
60 y
69 y
69 y
51 y
APOE 33
SORL1 +
70 y
60 y
55 y
52 y
42 y
61 y
52 y
APOE 33
SORL1 +

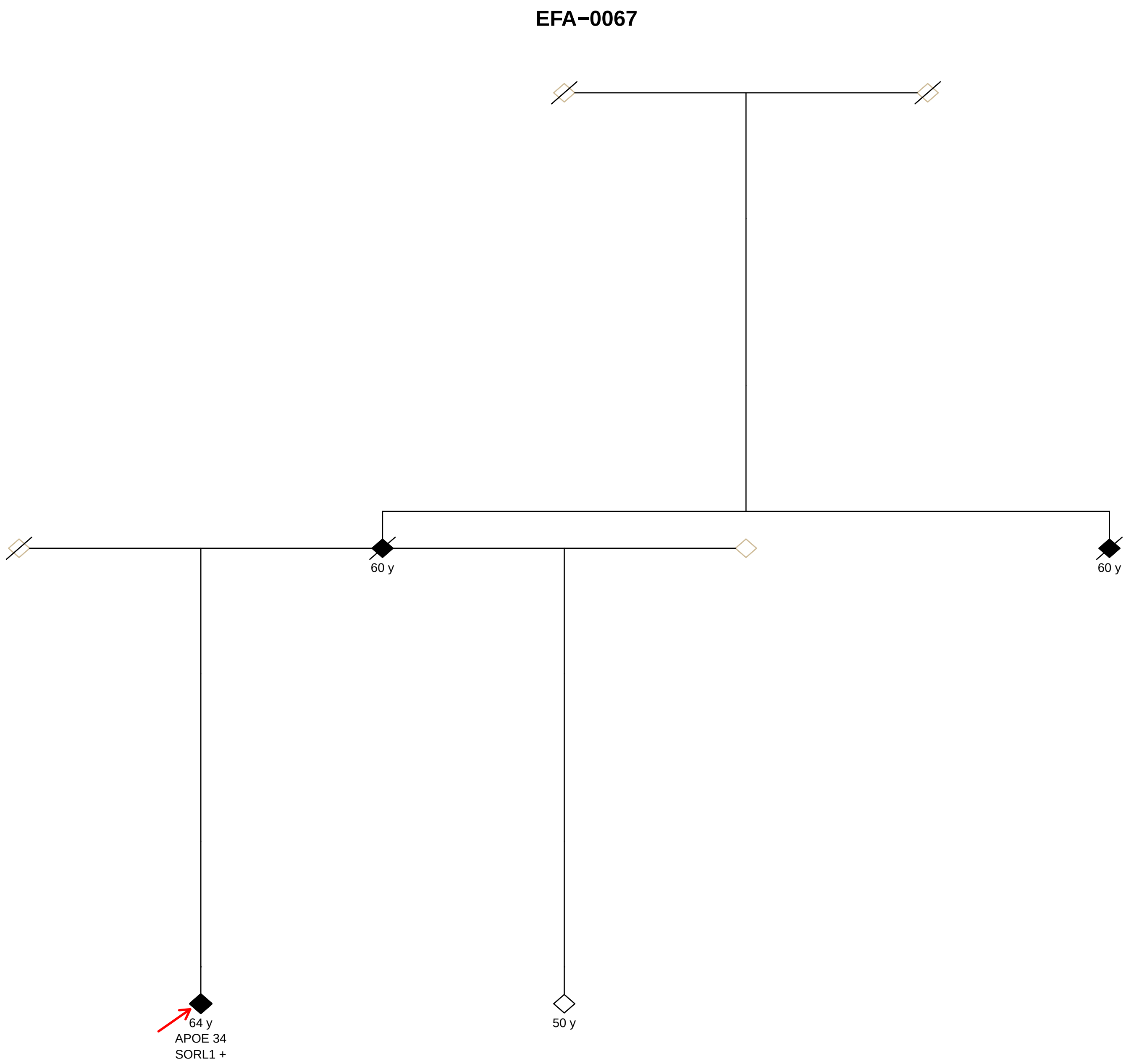
EFA−0067
60 y
60 y
64 y
APOE 34
SORL1 +
50 y

EFA−0280

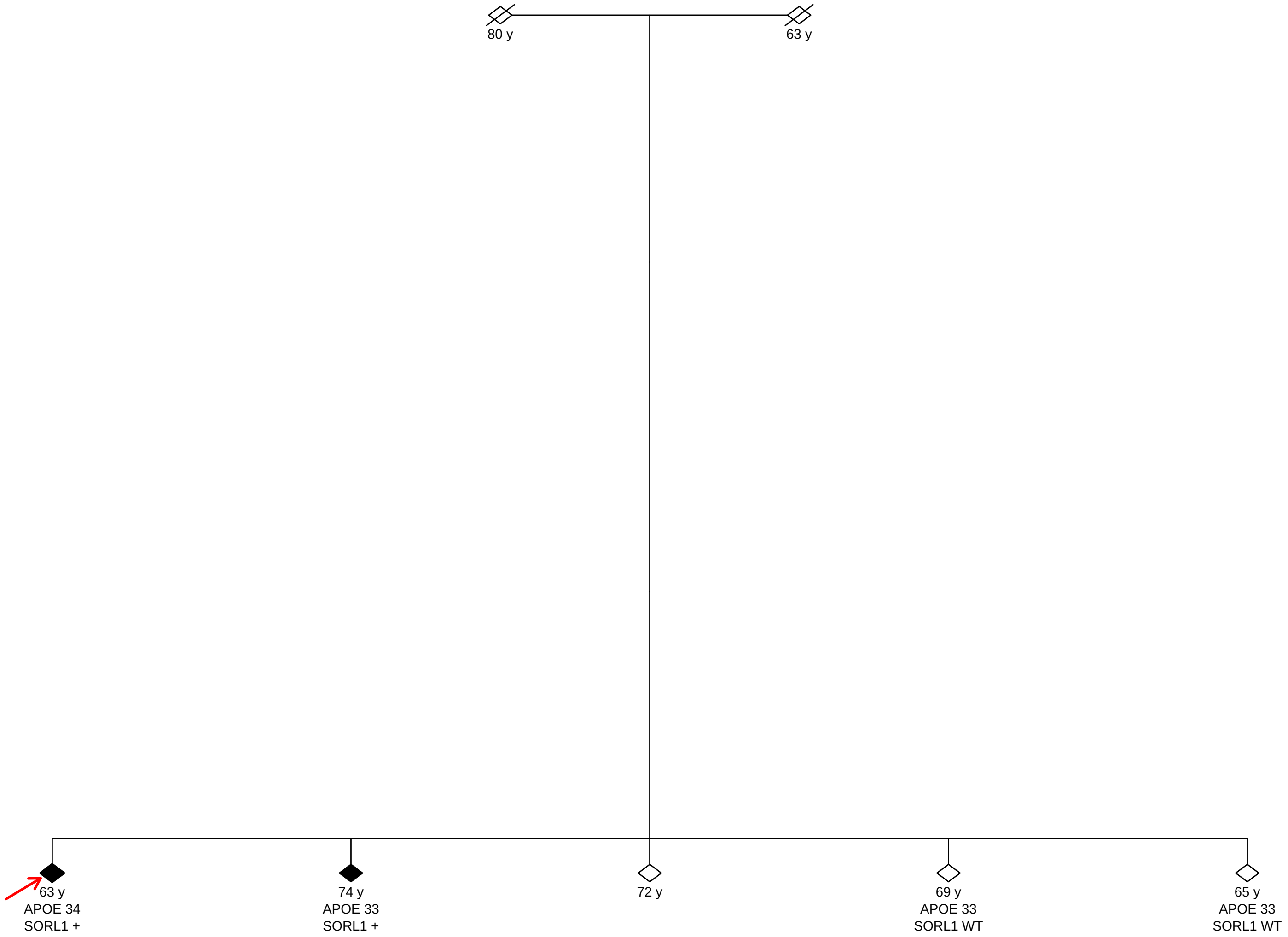
80 y
63 y
63 y
APOE 34
SORL1 +
74 y
APOE 33
SORL1 +
72 y
69 y
APOE 33
SORL1 WT
65 y
APOE 33
SORL1 WT

EXT−0049

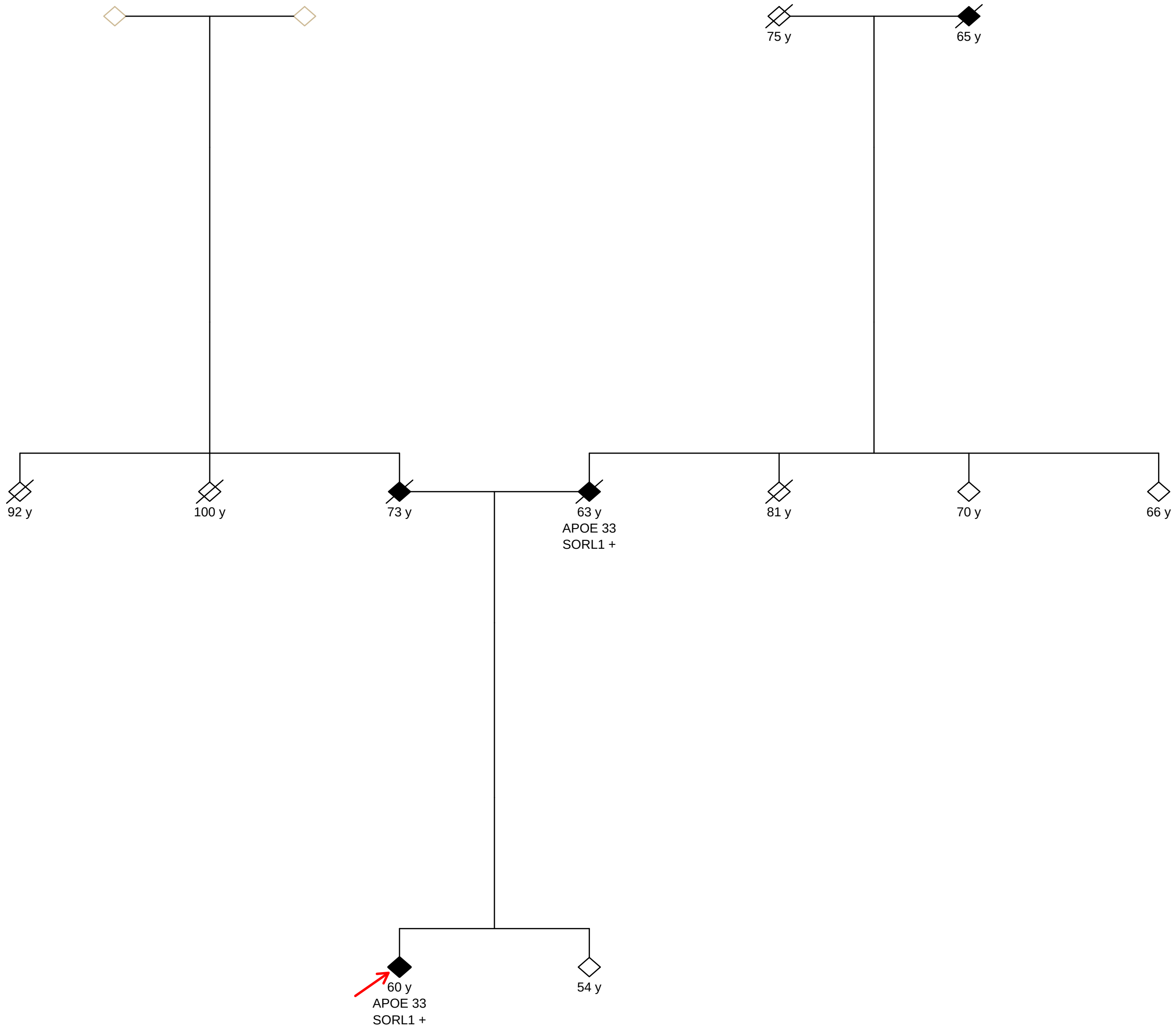
75 y
65 y
92 y
100 y
73 y
63 y
APOE 33
SORL1 +
81 y
70 y
66 y
60 y
APOE 33
SORL1 +
54 y

EXT−1231

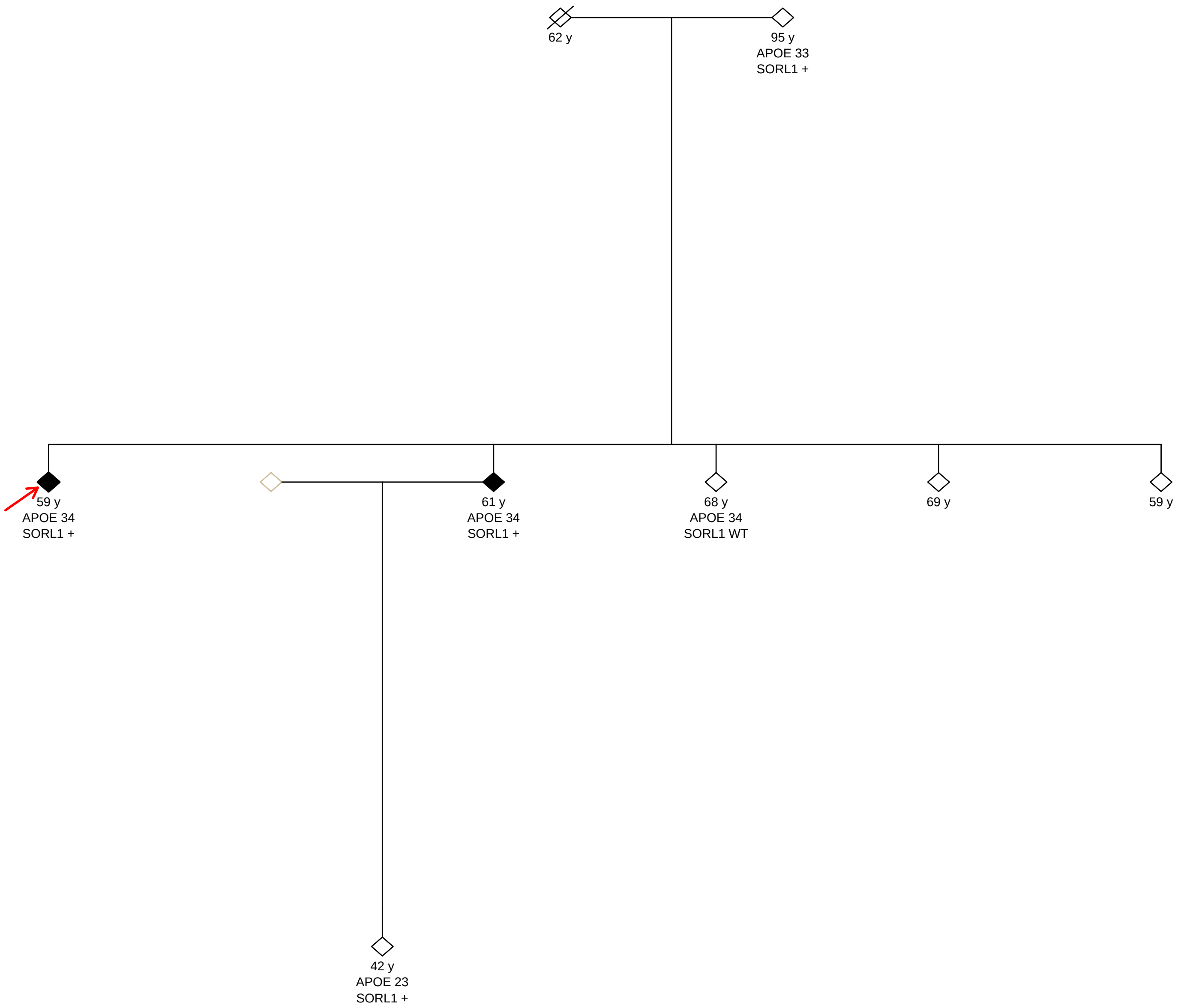

62 y
95 y
APOE 33
SORL1 +
59 y
APOE 34
SORL1 +
61 y
APOE 34
SORL1 +
68 y
APOE 34
SORL1 WT
69 y
59 y
42 y
APOE 23
SORL1 +

EXT−1906

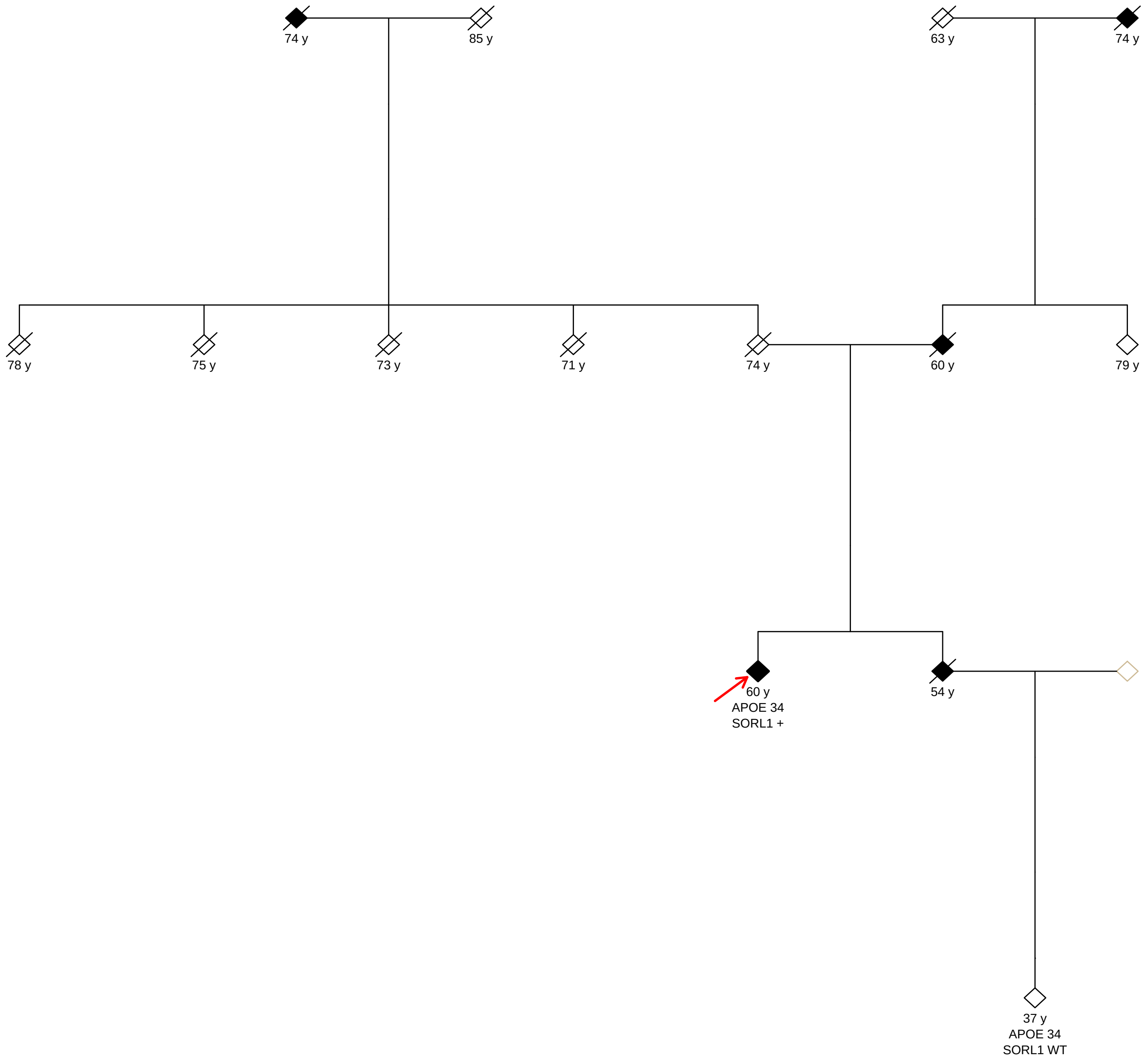

74 y
85 y
63 y
74 y
78 y
75 y
73 y
71 y
74 y
60 y
79 y
60 y
APOE 34
SORL1 +
54 y
37 y
APOE 34
SORL1 WT

EXT−0400

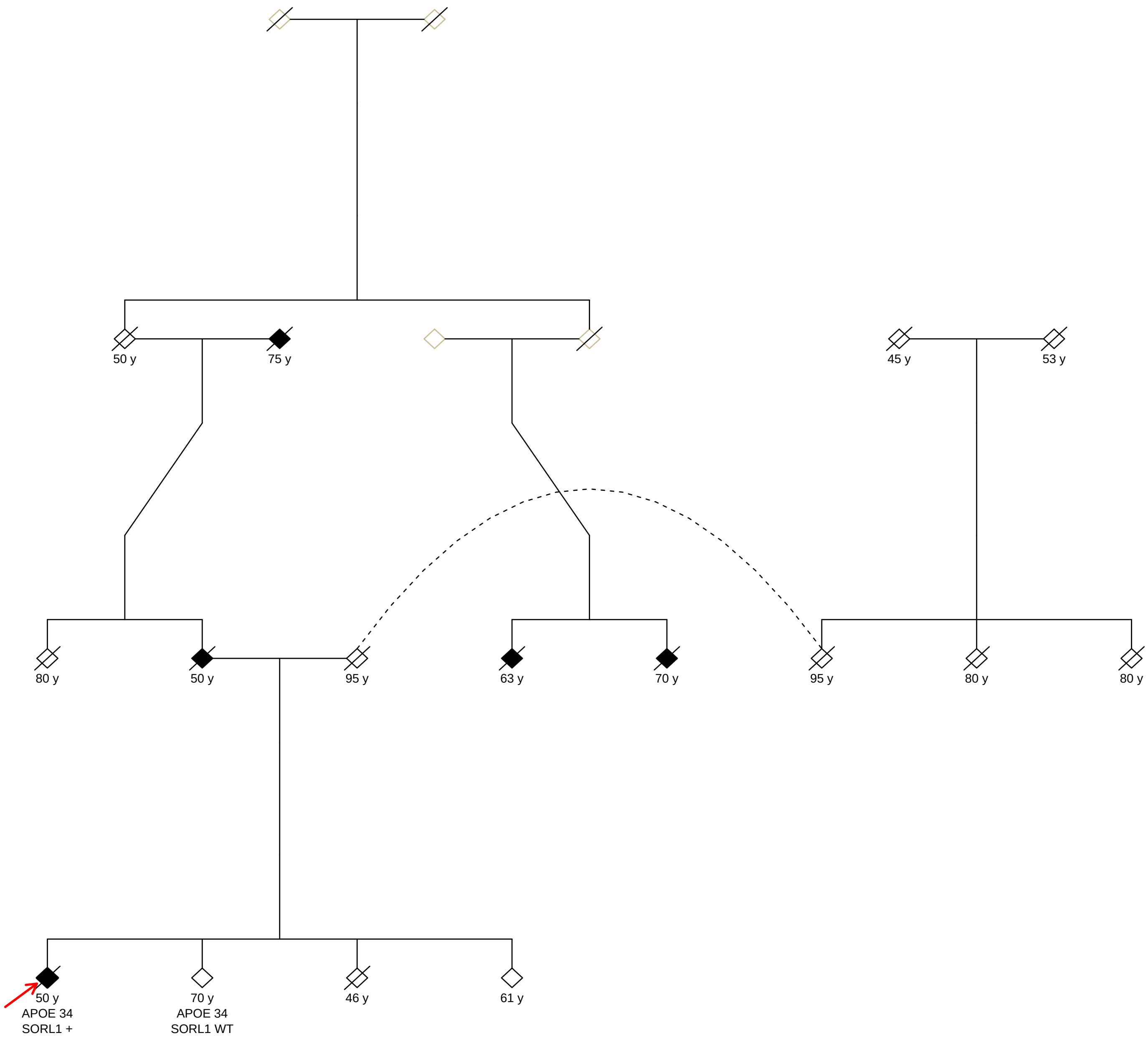

50 y
75 y
45 y
53 y
80 y
50 y
95 y
63 y
70 y
95 y
80 y
80 y
50 y
APOE 34
SORL1 +
70 y
APOE 34
SORL1 WT
46 y
61 y

EXT−0087

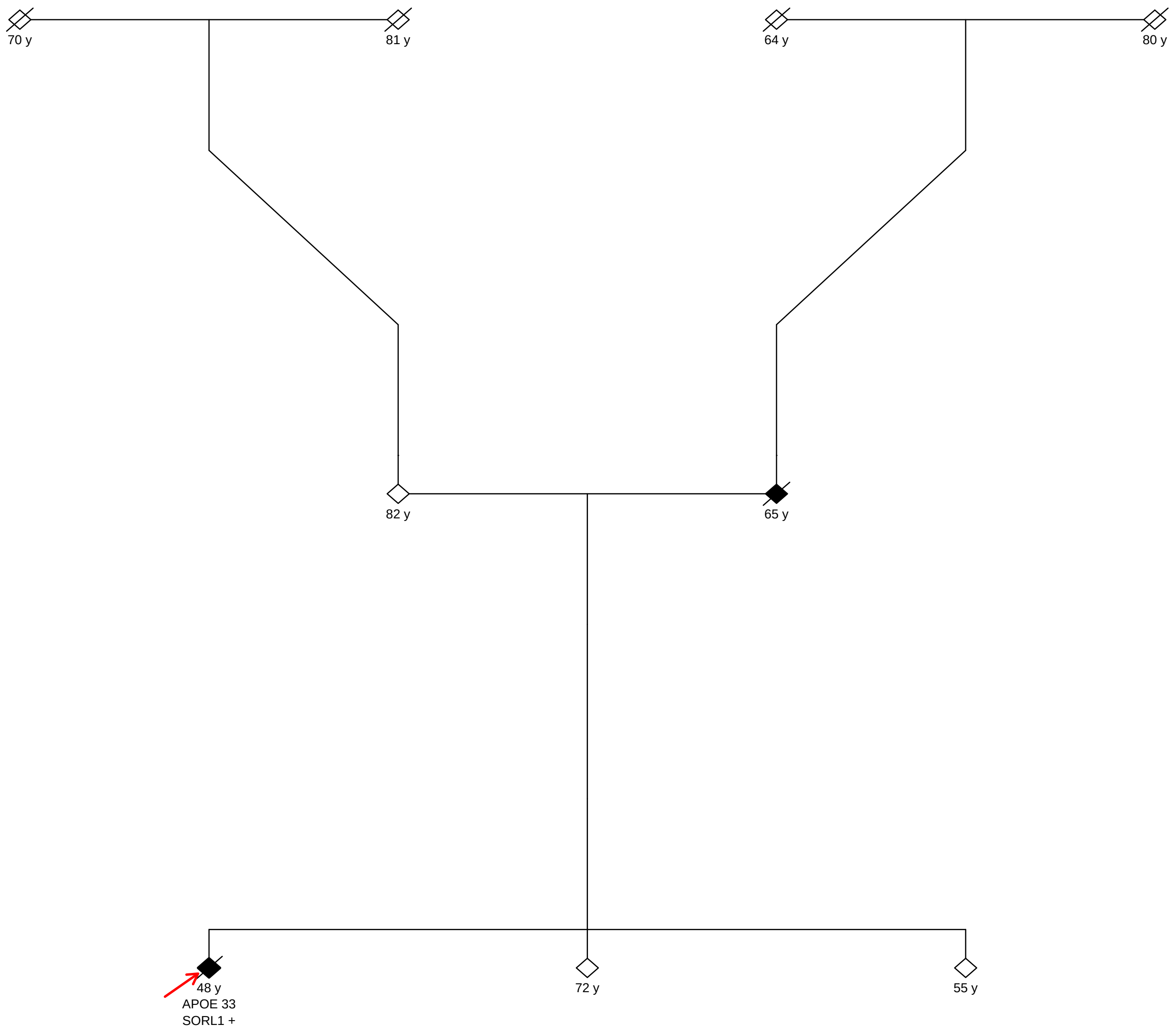

EXT−0438

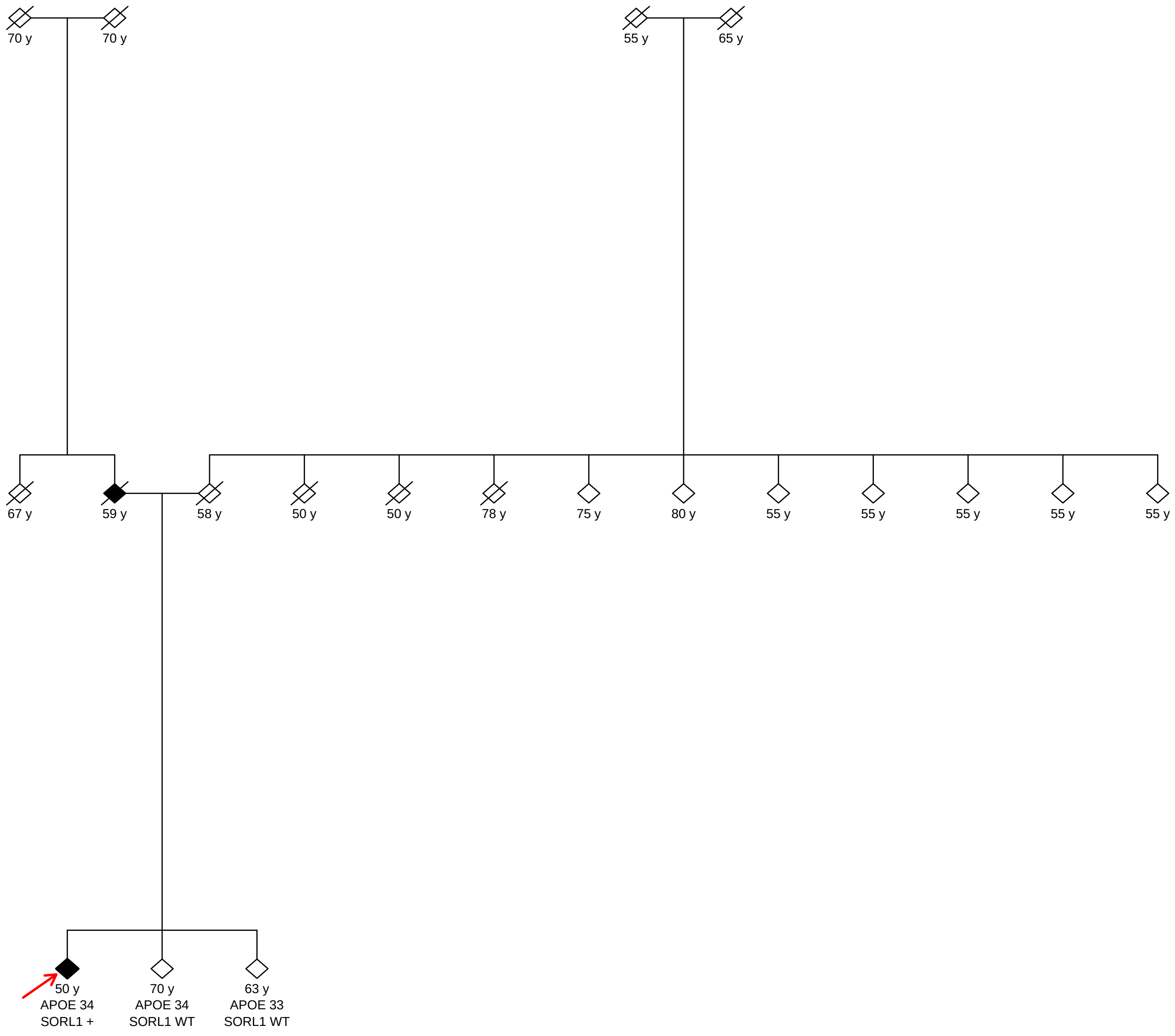

70 y
70 y
55 y
65 y
67 y
59 y
58 y
50 y
50 y
78 y
75 y
80 y
55 y
55 y
55 y
55 y
55 y
50 y
APOE 34
SORL1 +
70 y
APOE 34
SORL1 WT
63 y
APOE 33
SORL1 WT

EXT−1893

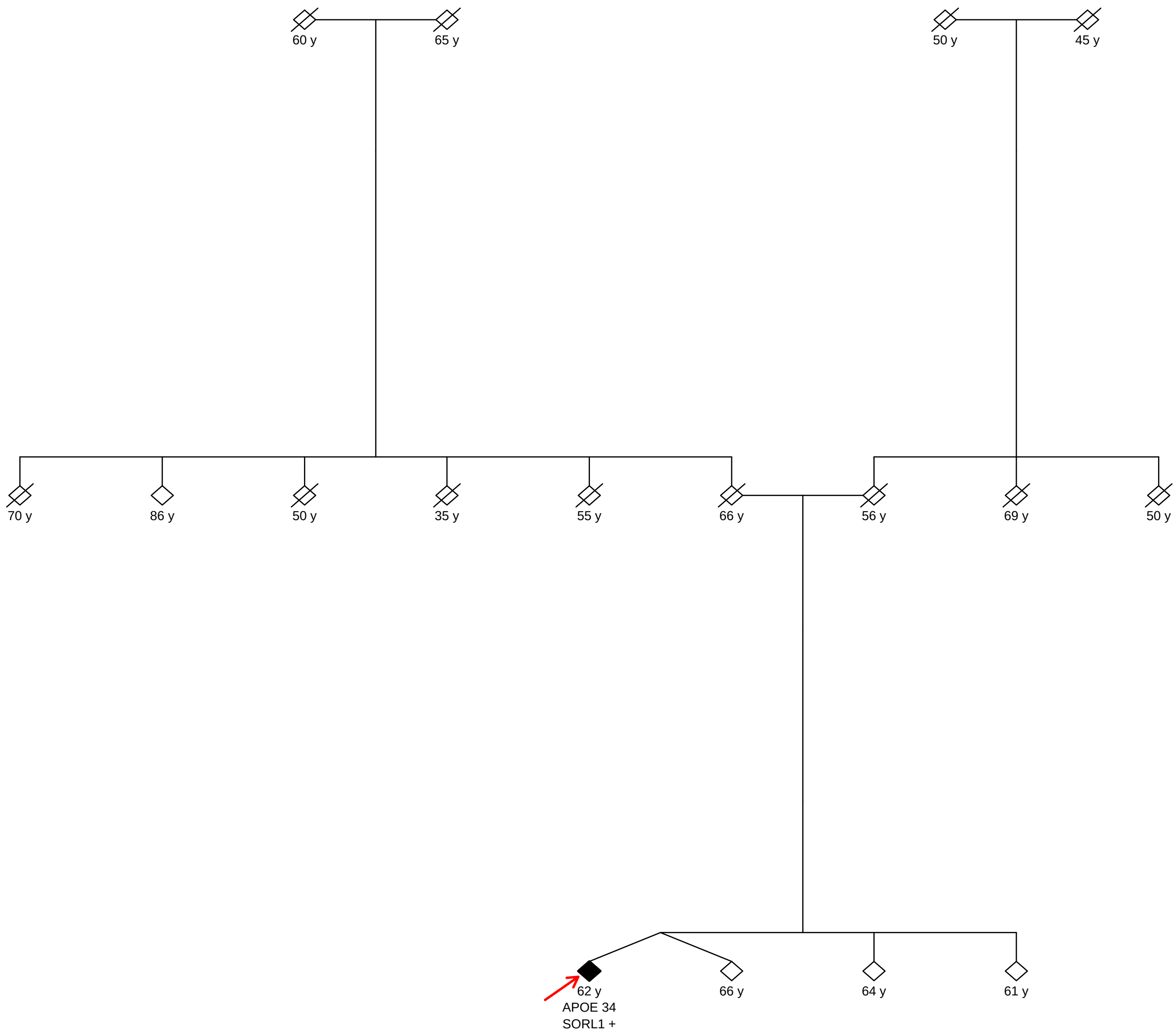
60 y
65 y
50 y
45 y
70 y
86 y
50 y
35 y
55 y
66 y
56 y
69 y
50 y
62 y
APOE 34
SORL1 +
66 y
64 y
61 y

EXT−0017

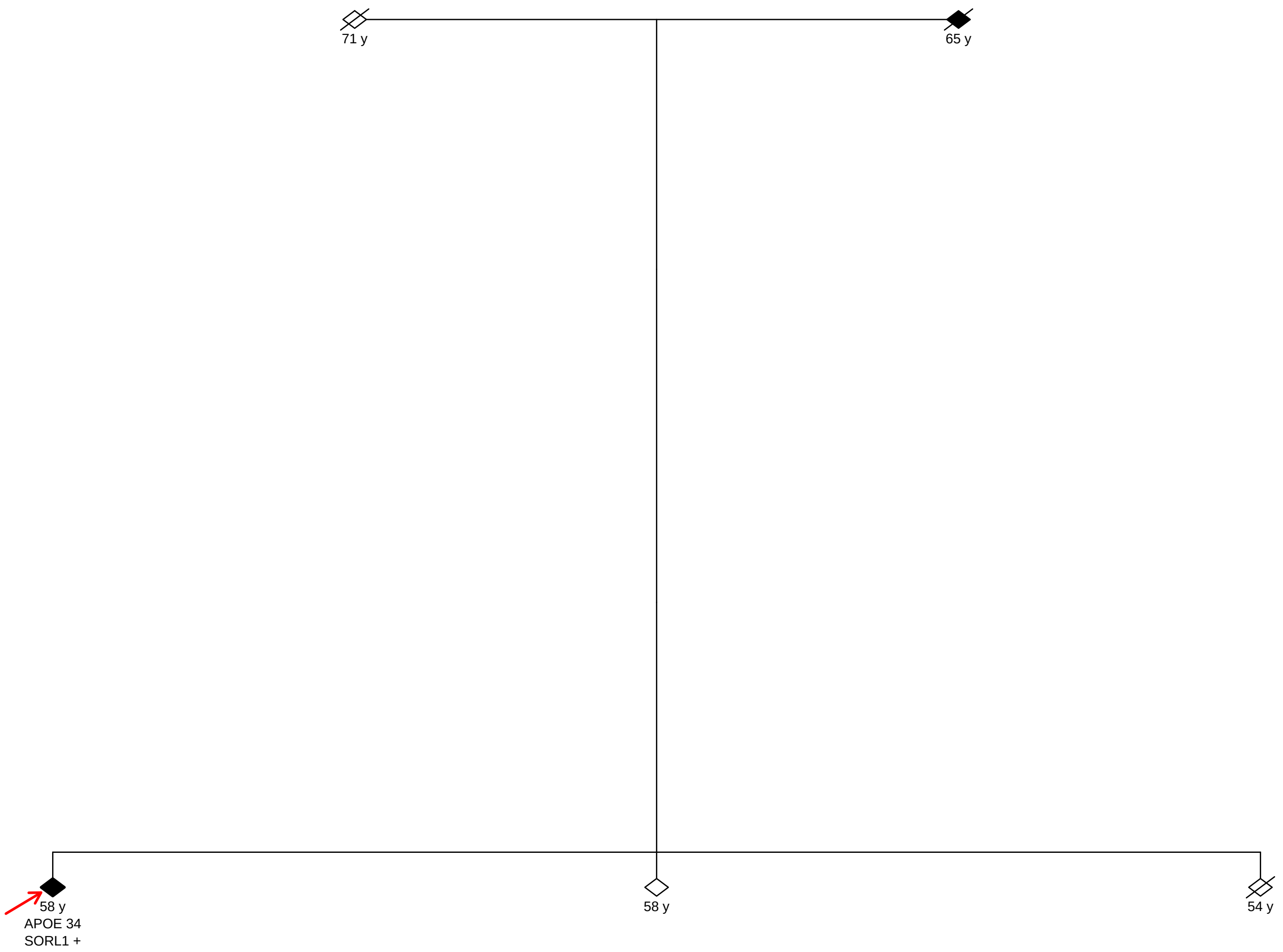

EXT−0290

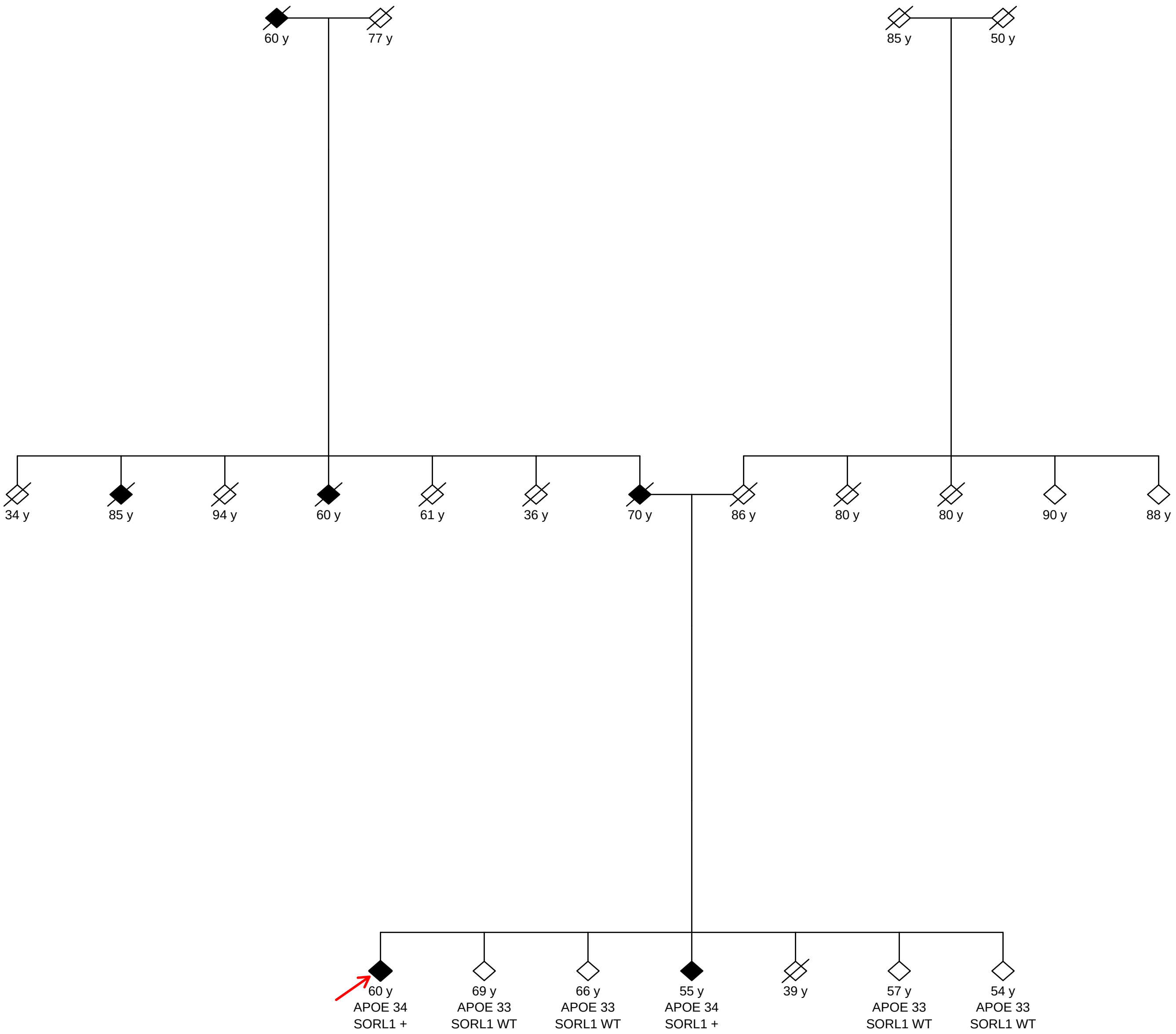

60 y
77 y
85 y
50 y
34 y
85 y
94 y
60 y
61 y
36 y
70 y
86 y
80 y
80 y
90 y
88 y
60 y APOE 34 SORL1 +
69 y APOE 33 SORL1 WT
66 y APOE 33 SORL1 WT
55 y APOE 34 SORL1 +
39 y
57 y APOE 33 SORL1 WT
54 y APOE 33 SORL1 WT

EXT−1106

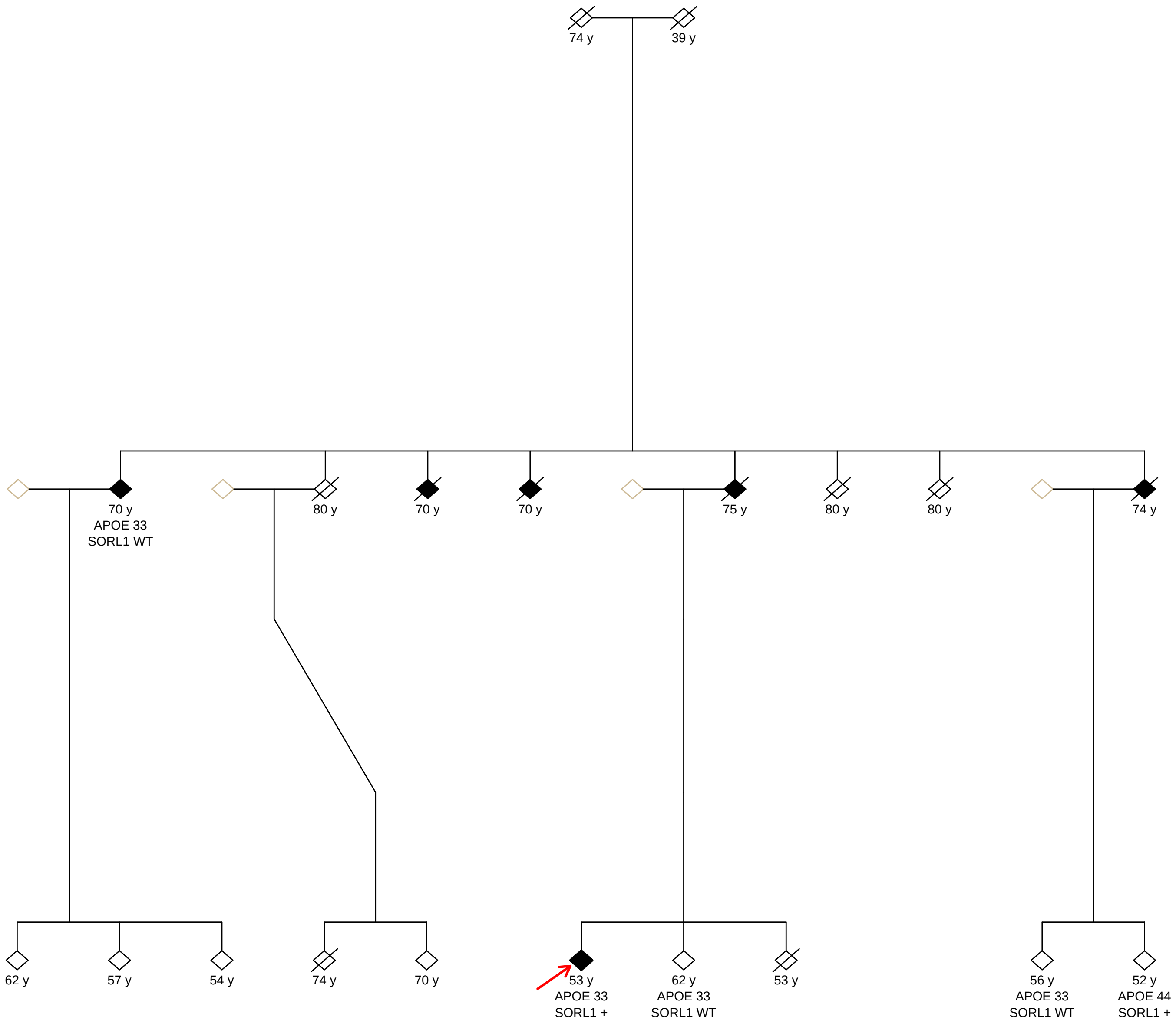
74 y
39 y
70 y
APOE 33
SORL1 WT
80 y
70 y
70 y
75 y
80 y
80 y
74 y
62 y
57 y
54 y
74 y
70 y
53 y
APOE 33
SORL1 +
62 y
APOE 33
SORL1 WT
53 y
56 y
APOE 33
SORL1 WT
52 y
APOE 44
SORL1 +

EXT−1756

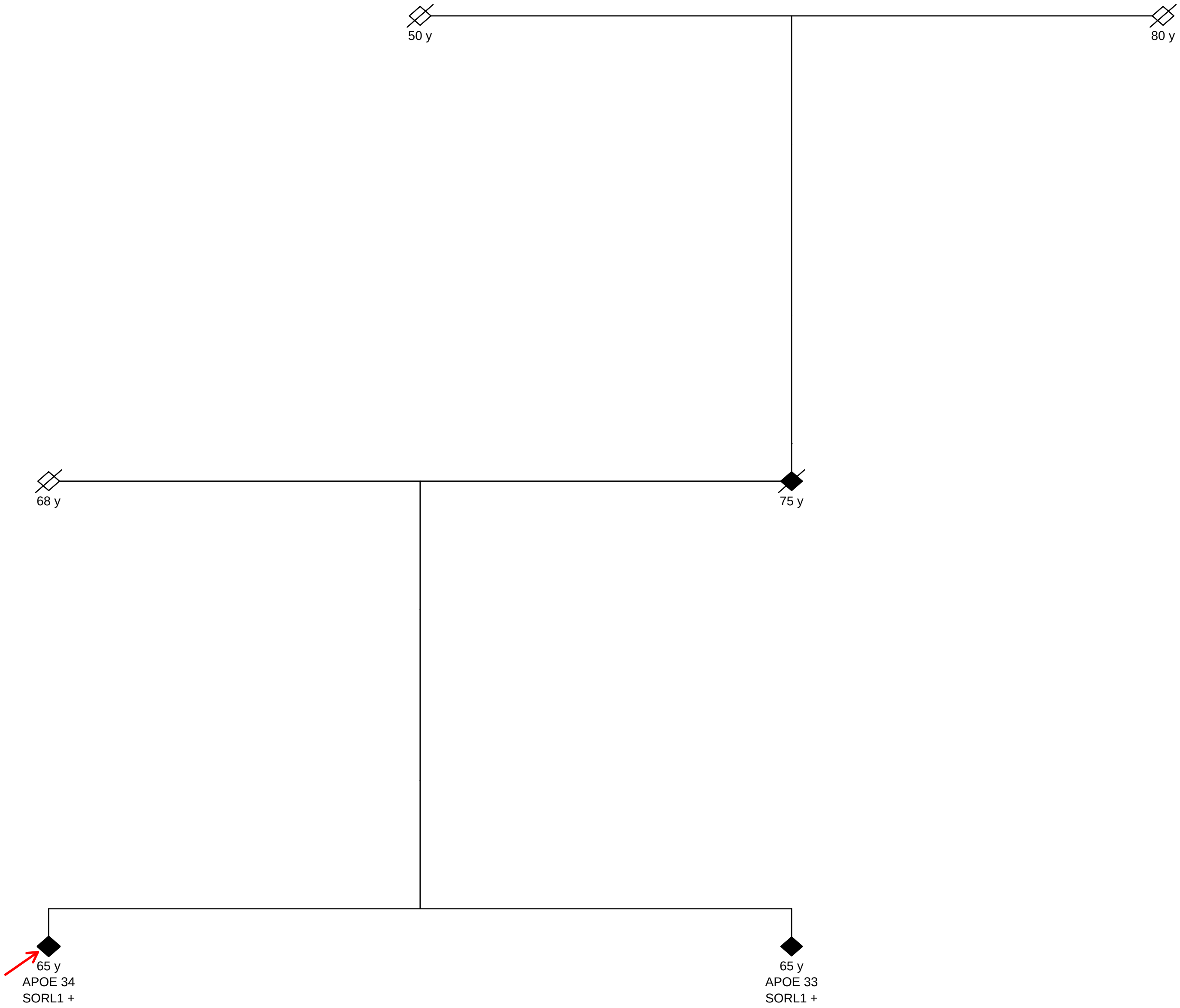

EXT−1023

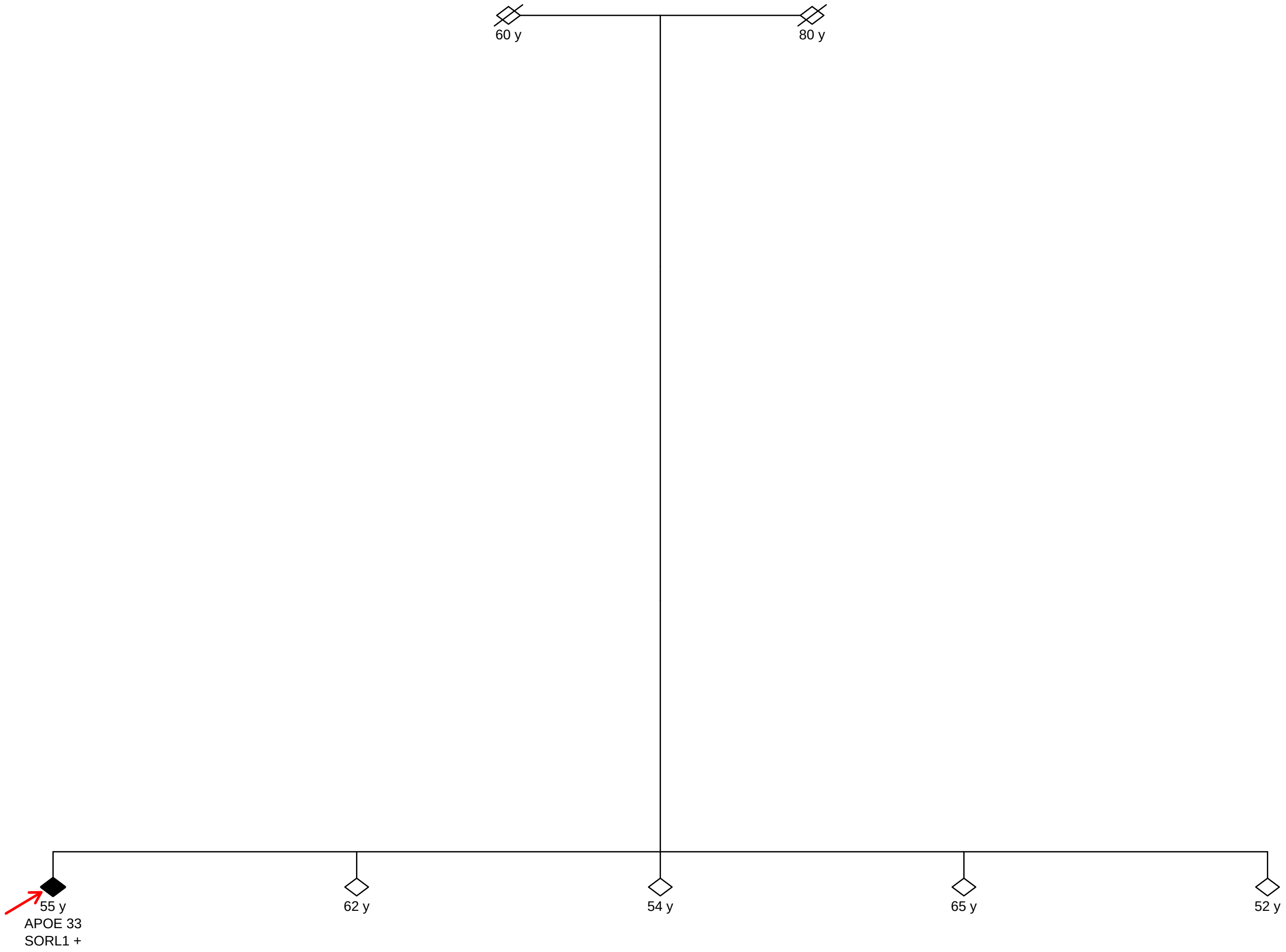

EXT−0050

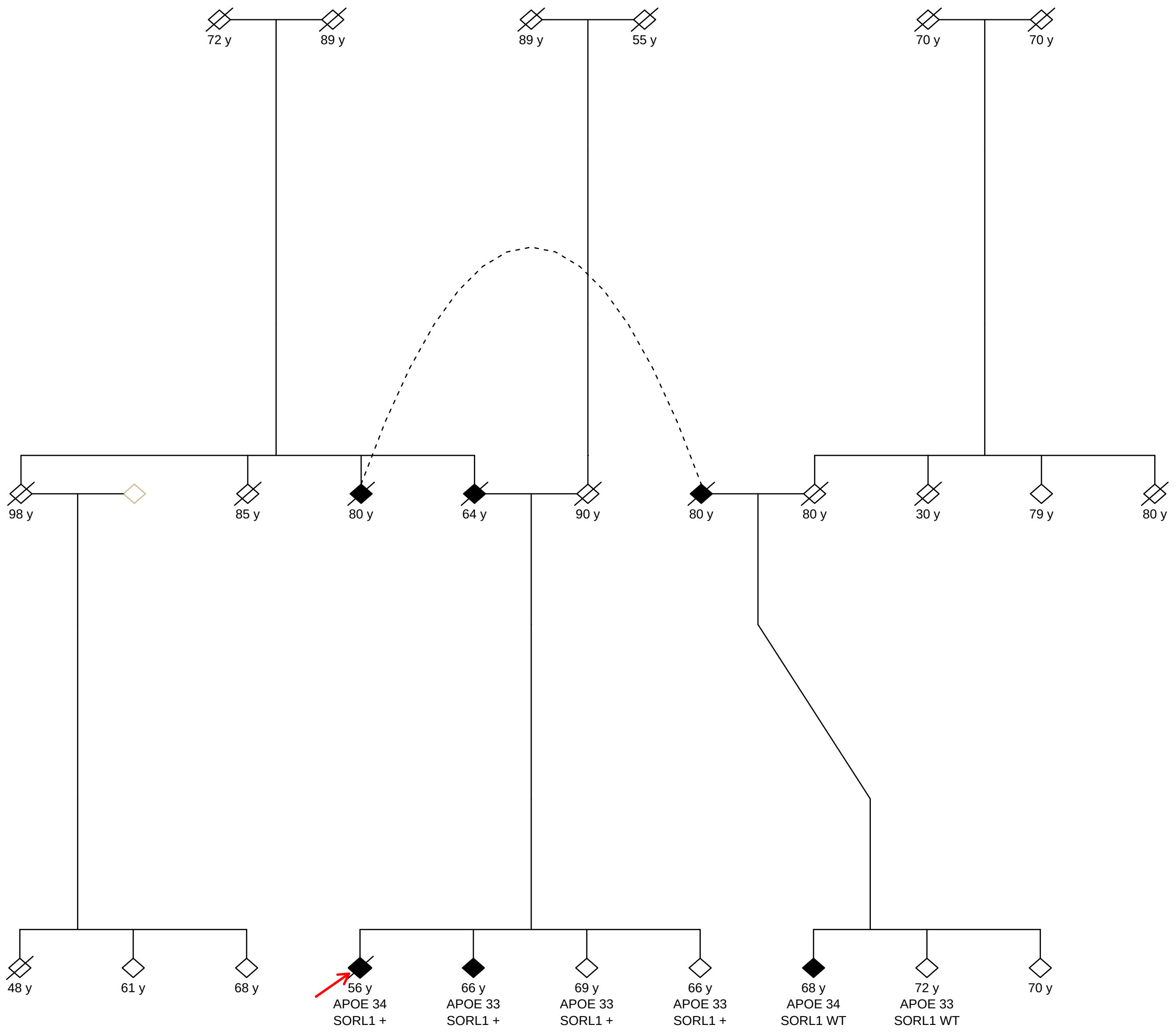
72 y
89 y
89 y
55 y
70 y
70 y
98 y
85 y
80 y
64 y
90 y
80 y
80 y
30 y
79 y
80 y
48 y
61 y
68 y
56 y
APOE 34
SORL1 +
66 y
APOE 33
SORL1 +
69 y
APOE 33
SORL1 +
66 y
APOE 33
SORL1 +
68 y
APOE 34
SORL1 WT
72 y
APOE 33
SORL1 WT
70 y

RFA−0033

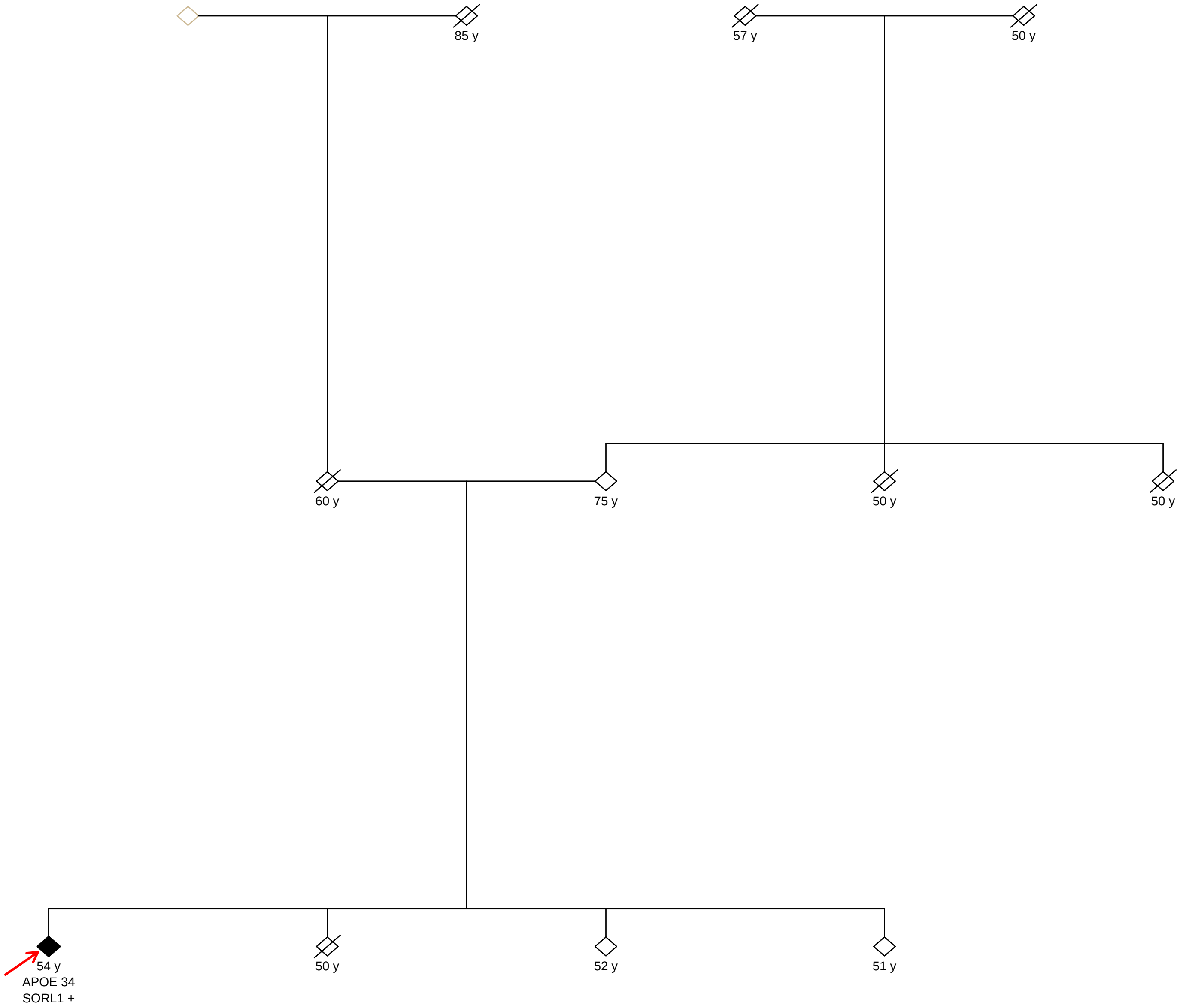

ROU−0309

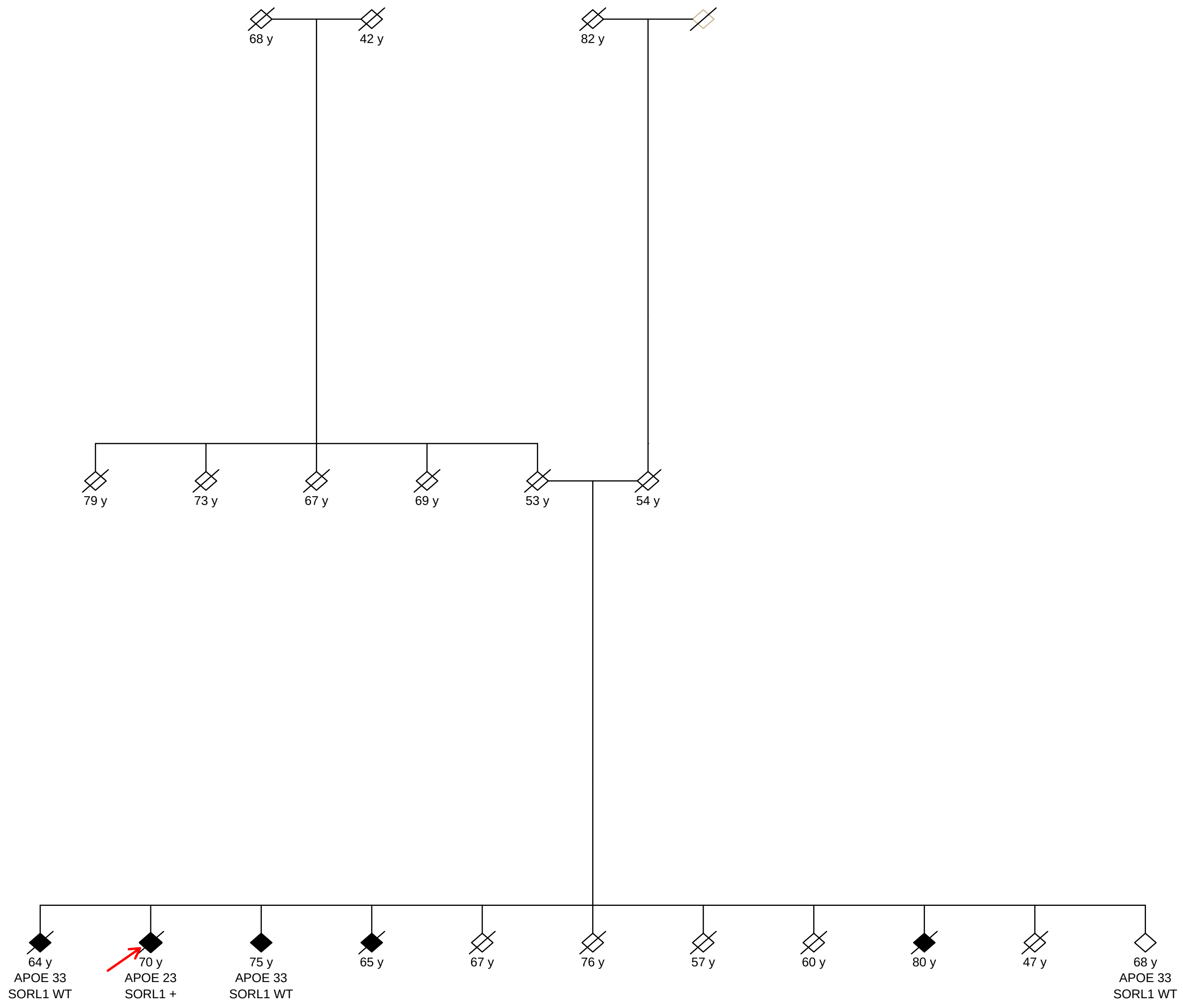
68 y
42 y
82 y
79 y
73 y
67 y
69 y
53 y
54 y
64 y
APOE 33
SORL1 WT
70 y
APOE 23
SORL1 +
75 y
APOE 33
SORL1 WT
65 y
67 y
76 y
57 y
60 y
80 y
47 y
68 y
APOE 33
SORL1 WT

ROU−0055

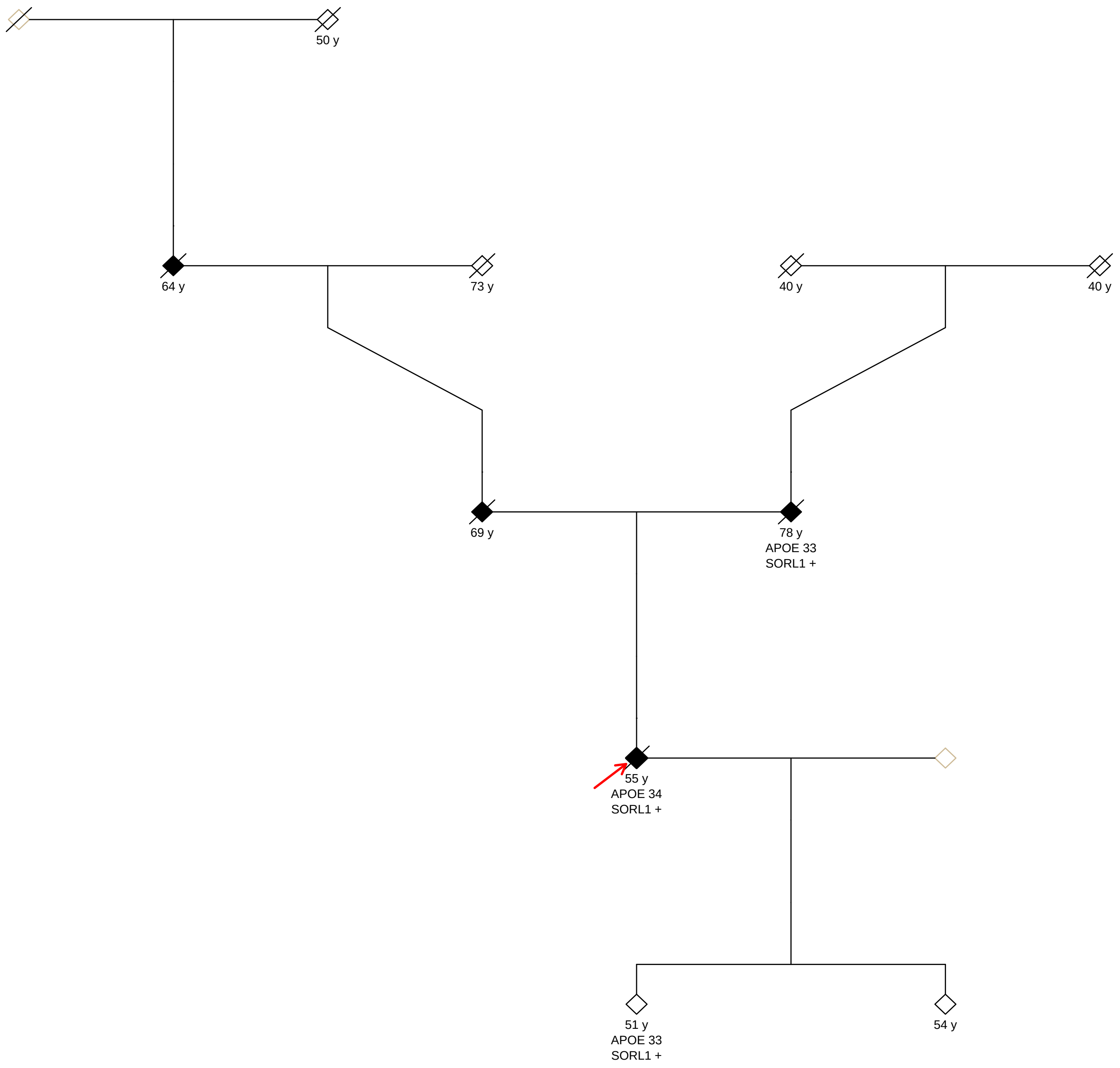

50 y
64 y
73 y
40 y
40 y
69 y
78 y
APOE 33
SORL1 +
55 y
APOE 34
SORL1 +
51 y
APOE 33
SORL1 +
54 y

ROU−1766

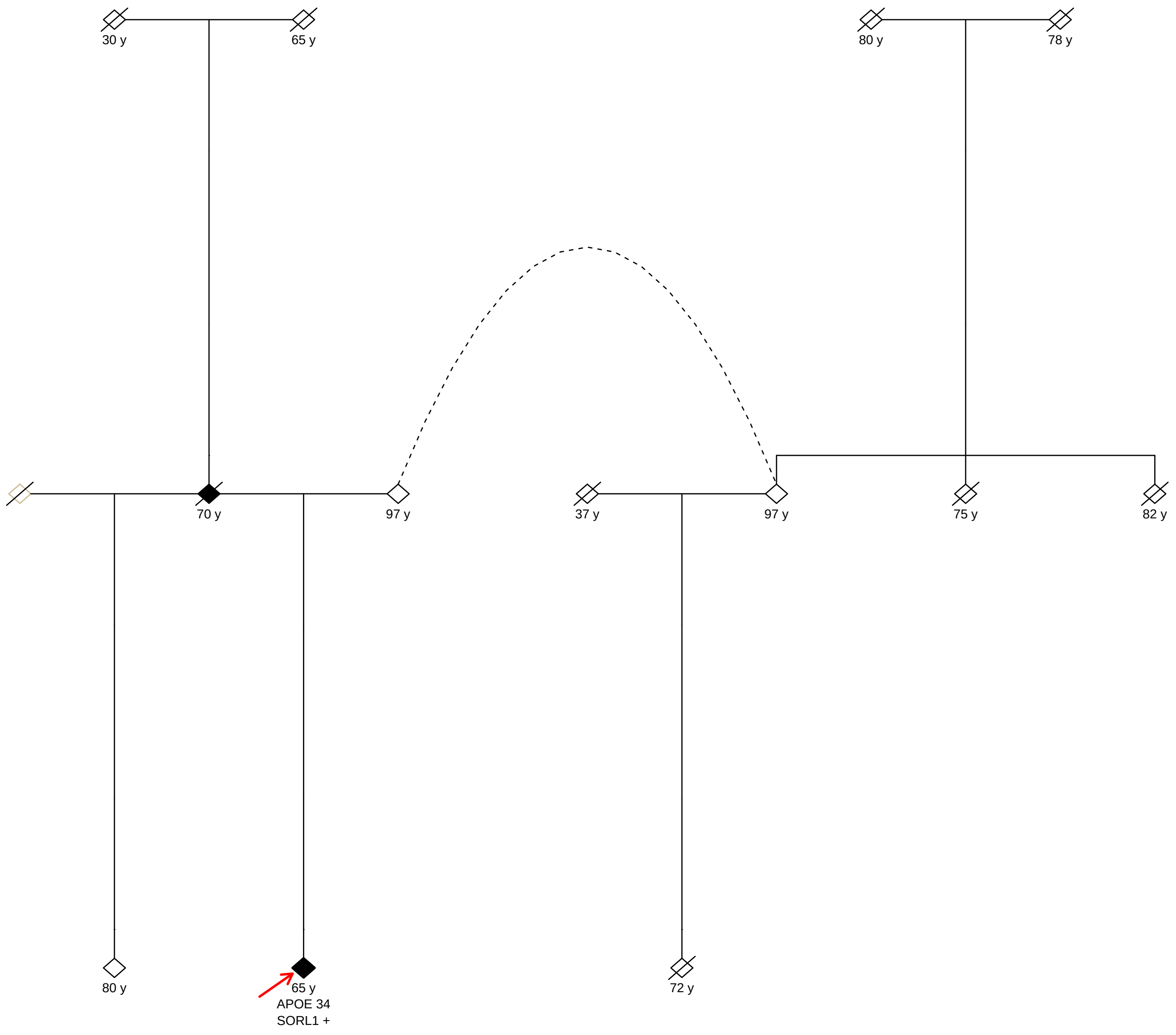
30 y
65 y
80 y
78 y
70 y
97 y
37 y
97 y
75 y
82 y
80 y
65 y
APOE 34
SORL1 +
72 y

ROU−1622

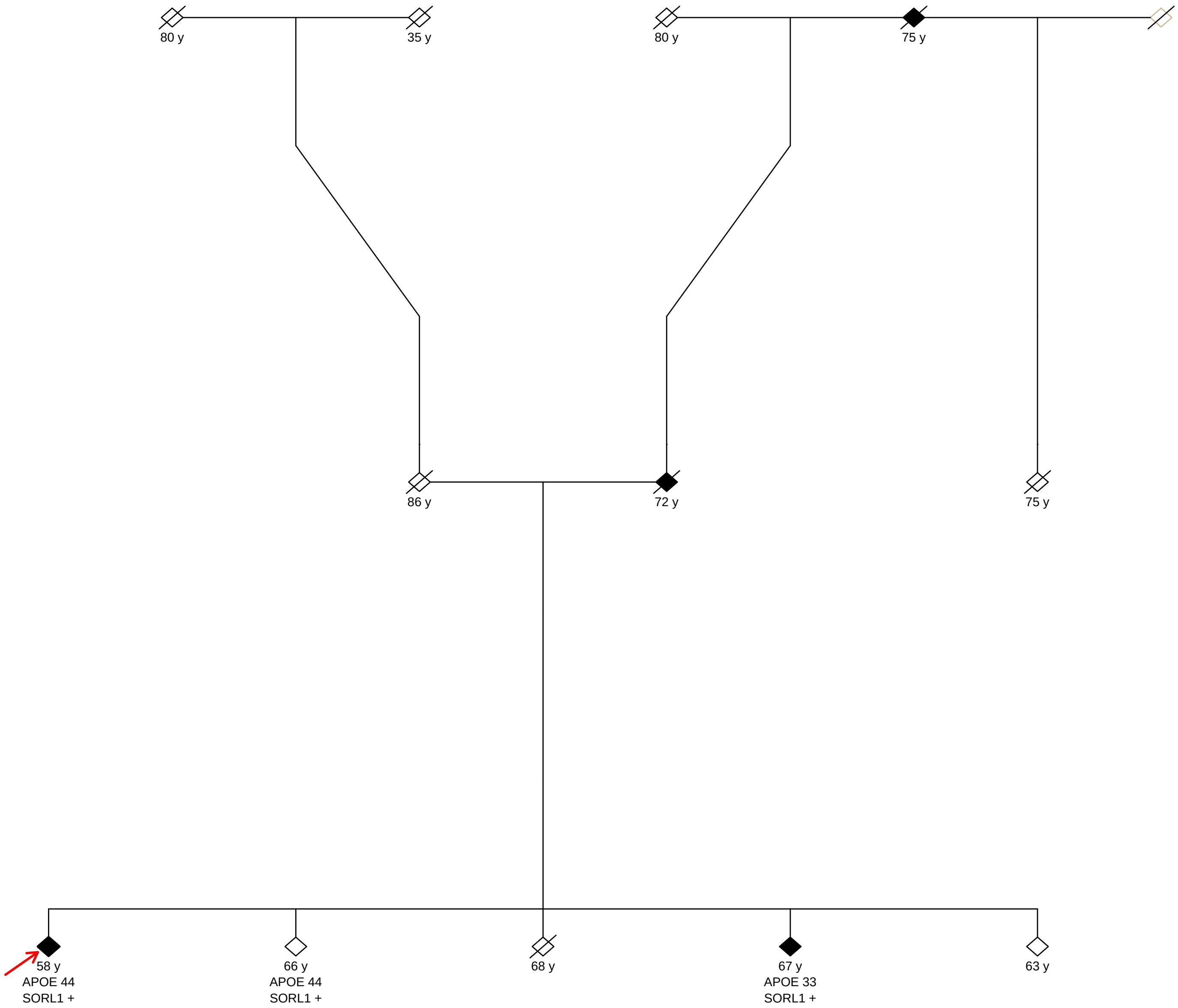
80 y
35 y
80 y
75 y
86 y
72 y
75 y
58 y
APOE 44
SORL1 +
66 y
APOE 44
SORL1 +
68 y
67 y
APOE 33
SORL1 +
63 y

ROU−1376

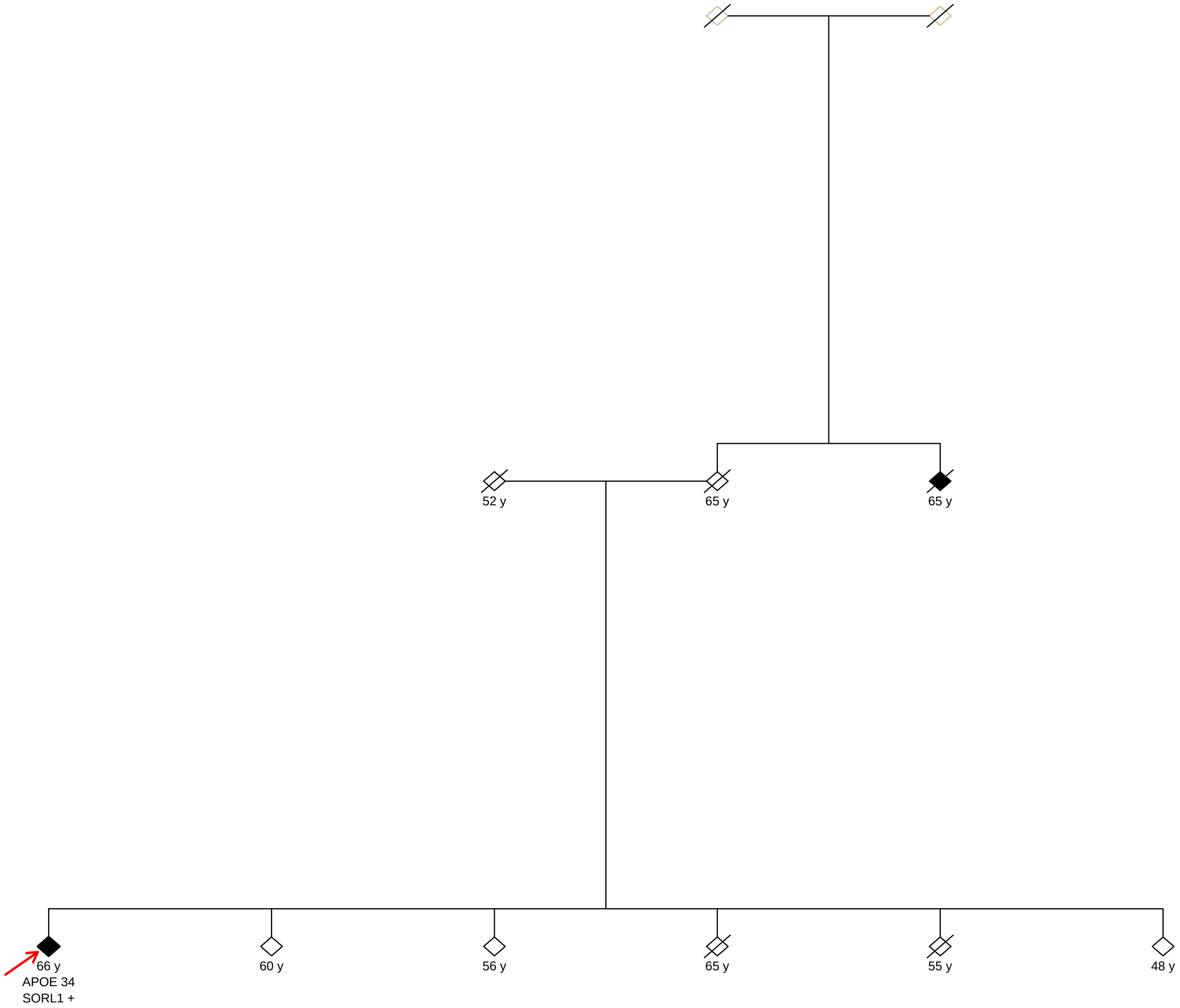

ROU−1409

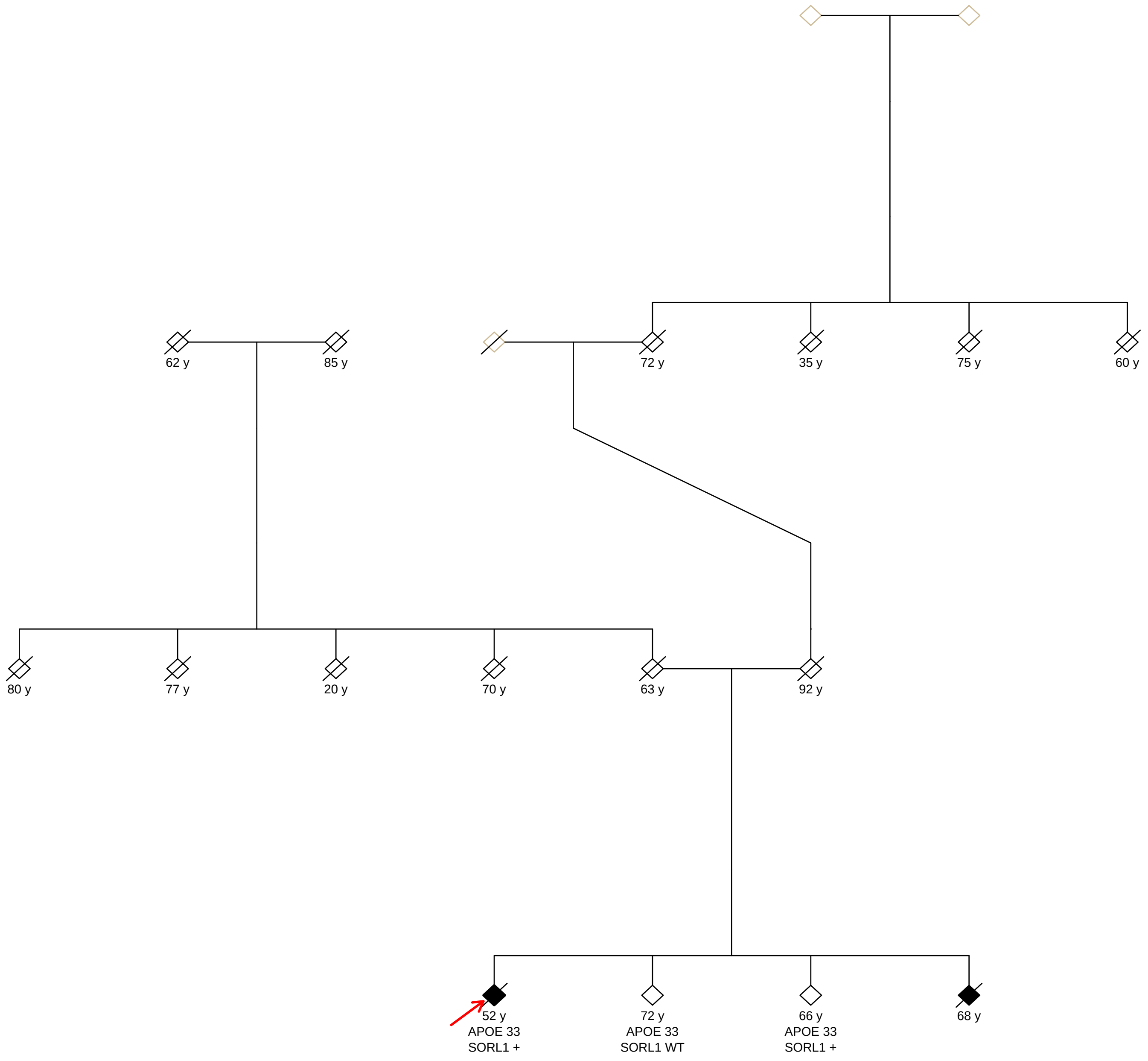
62 y
85 y
72 y
35 y
75 y
60 y
80 y
77 y
20 y
70 y
63 y
92 y
52 y
APOE 33
SORL1 +
72 y
APOE 33
SORL1 WT
66 y
APOE 33
SORL1 +
68 y

ROU−0699

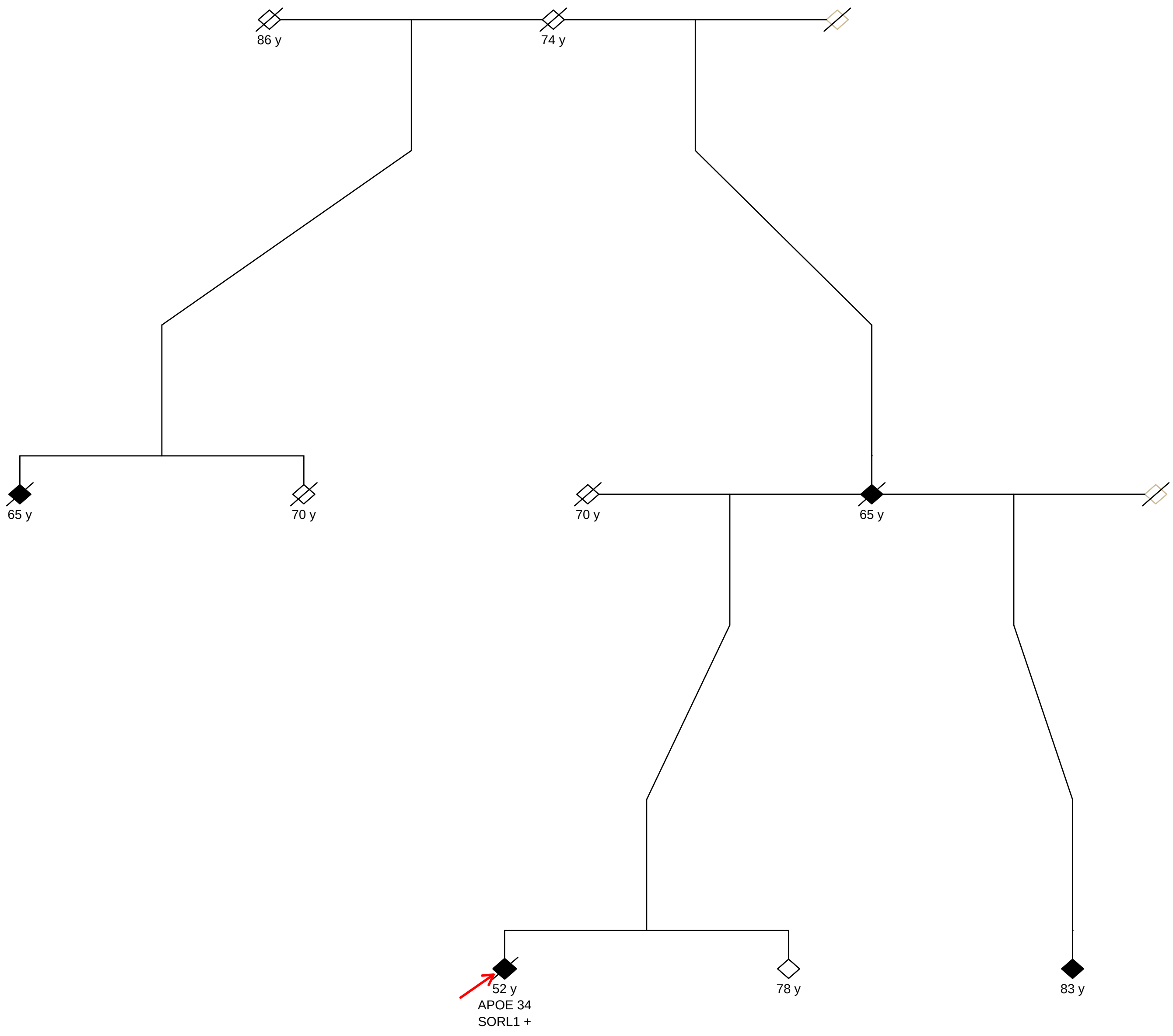

SAL−0621

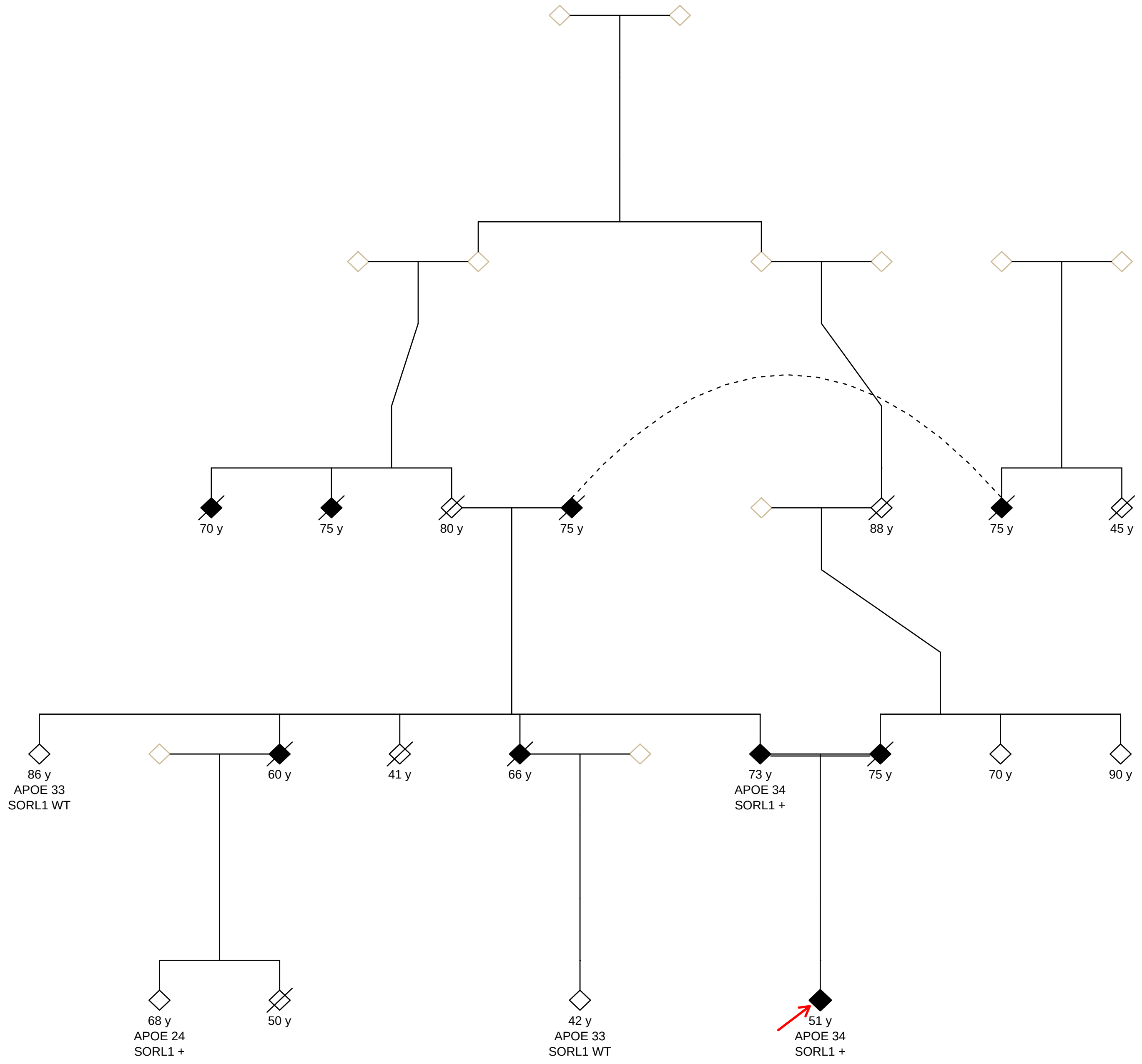
70 y
75 y
80 y
75 y
88 y
75 y
45 y
86 y
APOE 33
SORL1 WT
60 y
41 y
66 y
73 y
APOE 34
SORL1 +
75 y
70 y
90 y
68 y
APOE 24
SORL1 +
50 y
42 y
APOE 33
SORL1 WT
51 y
APOE 34
SORL1 +

Supplementary discussion and perspective about the model

Our model might be further developed to better take into account the following points and thus help refining estimations and apply to other diseases: controlling for ascertainment biases in other ways than removing probands phenotypes information, applying other strategies for cut-off piecewise constant functions, adjusting for family clusters or other genetic factors and taking death as a competing risk.

The control for ascertainment bias. As probands were selected based on age at onset, we proposed to exclude their phenotypes from model parameters computation (M-step) in order to correct for resulting putative ascertainment bias. Our simulation study showed that this method standardly used in family analyses efficiently helps to correct for this bias, but might lead to an underestimation of parameters at the beginning of the curve. Another approach, based on raking estimators, and more often used in survey analyses [3], could be developed to assess if such a strategy could better handle this ascertainment bias by incorporating auxiliary information in the procedure and thus adjust for family recruitment probabilities.

The choice of optimal cut-offs in piecewise constant function. The effect associated with the *SORL1* LoF variants was modeled using a simple but interpretable piecewise constant function. Because we did not know how the instantaneous risk associated with variants changes over time, we compared several models including different cut-offs and selected the best model based on BIC. A possible extension of this method is the repetition of this procedure through bootstrap iterations and a higher quantity of candidate cut-offs resulting in smoother curves [4]. However, non parametric and semi-parametric models like for example the Cox's model [5] may be also good candidate models conditionally to their ease of interpretation.

Adjustment for family cluster. For the sake of simplicity, we considered that individuals were independent conditionally to their *APOE* $\times$ *SORL1* genotypes. However, as shown by our simulation study, inter-family heterogeneity may lead to misestimation of model parameters. Stratified models and then frailty survival models were developed to handle this kind of data [6]. The latter proposes to incorporate random term(s) in the model mimicking an intra-familial correlation and thus reduce the part of variability associated with *SORL1* variants effect.

The death as competing risk. Here, we have chosen to consider death as censoring, meaning that death and disease onset are independent. Indeed, there is no demonstrated increased risk of death linked to *SORL1* LoF variants. In contrast, *APOE*-ε4 is related to a higher risk of mortality independently of AD [7]. Since this was already taken into account in published data we used for baseline risk according to *APOE*, we did not need to adjust a second time for it [1]. However, in future applications of this methodology to the case of other diseases, the M-step may easily incorporate models developed in a competing risk framework [8].

Supplementary figures

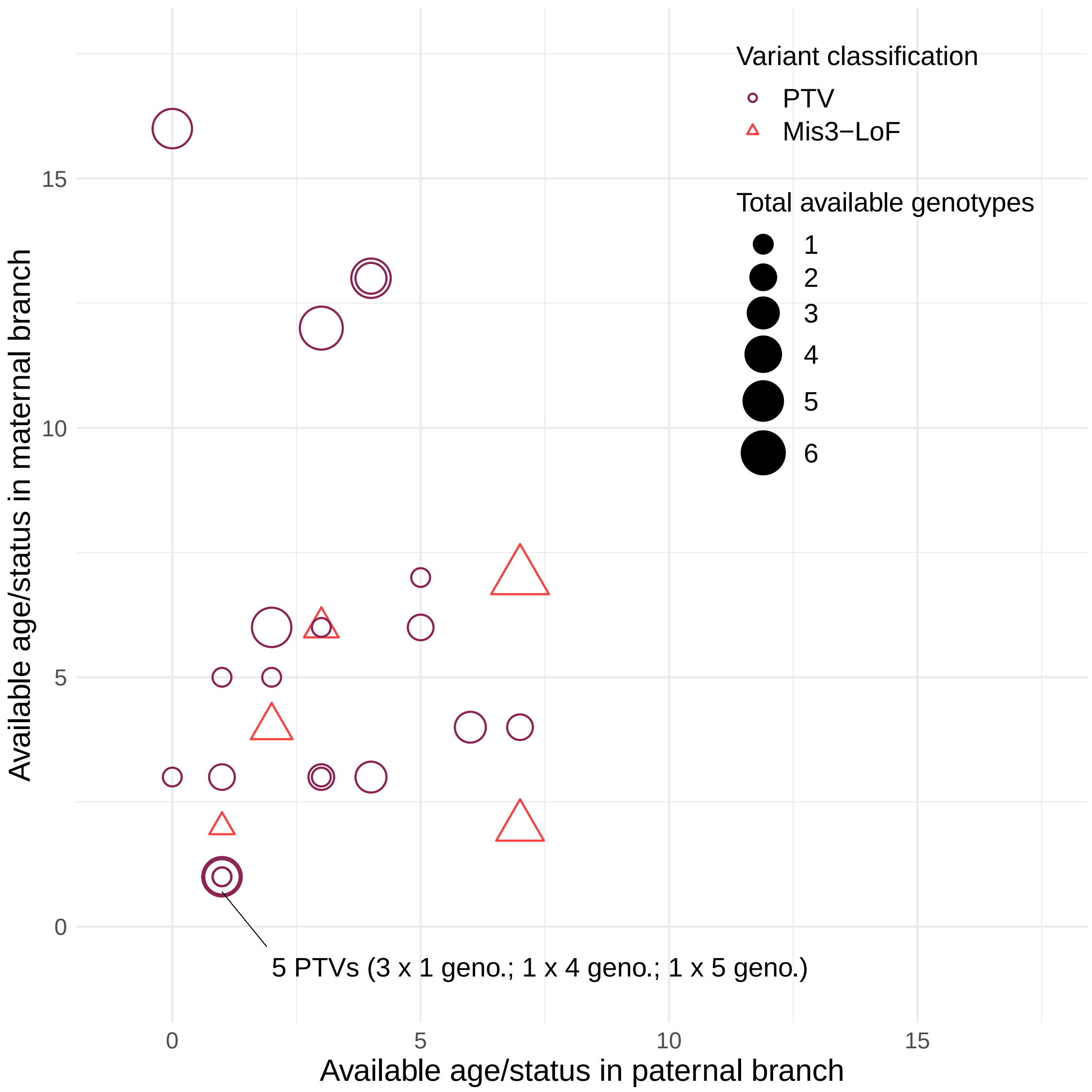

Fig. S1: Number of informative phenotypes available in maternal versus paternal branch over the 27 LoF families.

Each point represents a family. Point shape/color corresponds to the variant classification and point size corresponds to the total number of genotypes (*APOE* and *SORL1*) available in the family, including the proband genotype. The X-axis (respectively the Y-axis) represents the number of individuals in the paternal (respectively maternal) branch with informative phenotype (i.e. status and age $\geq$ 40 years old). Of notes, X- and Y- axes do not count probands, siblings, nieces/nephews nor those that are not related by blood.

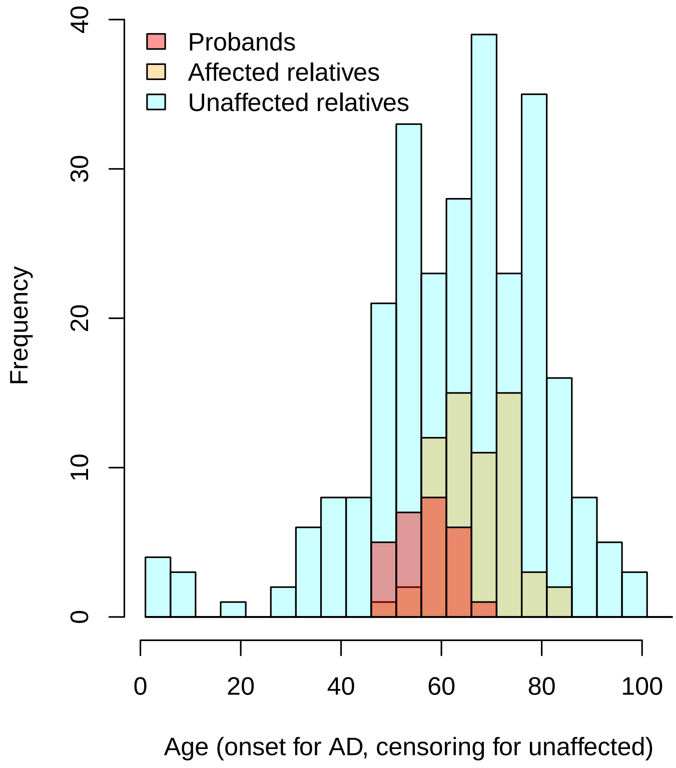

Fig. S2: Age distribution in LoF families.

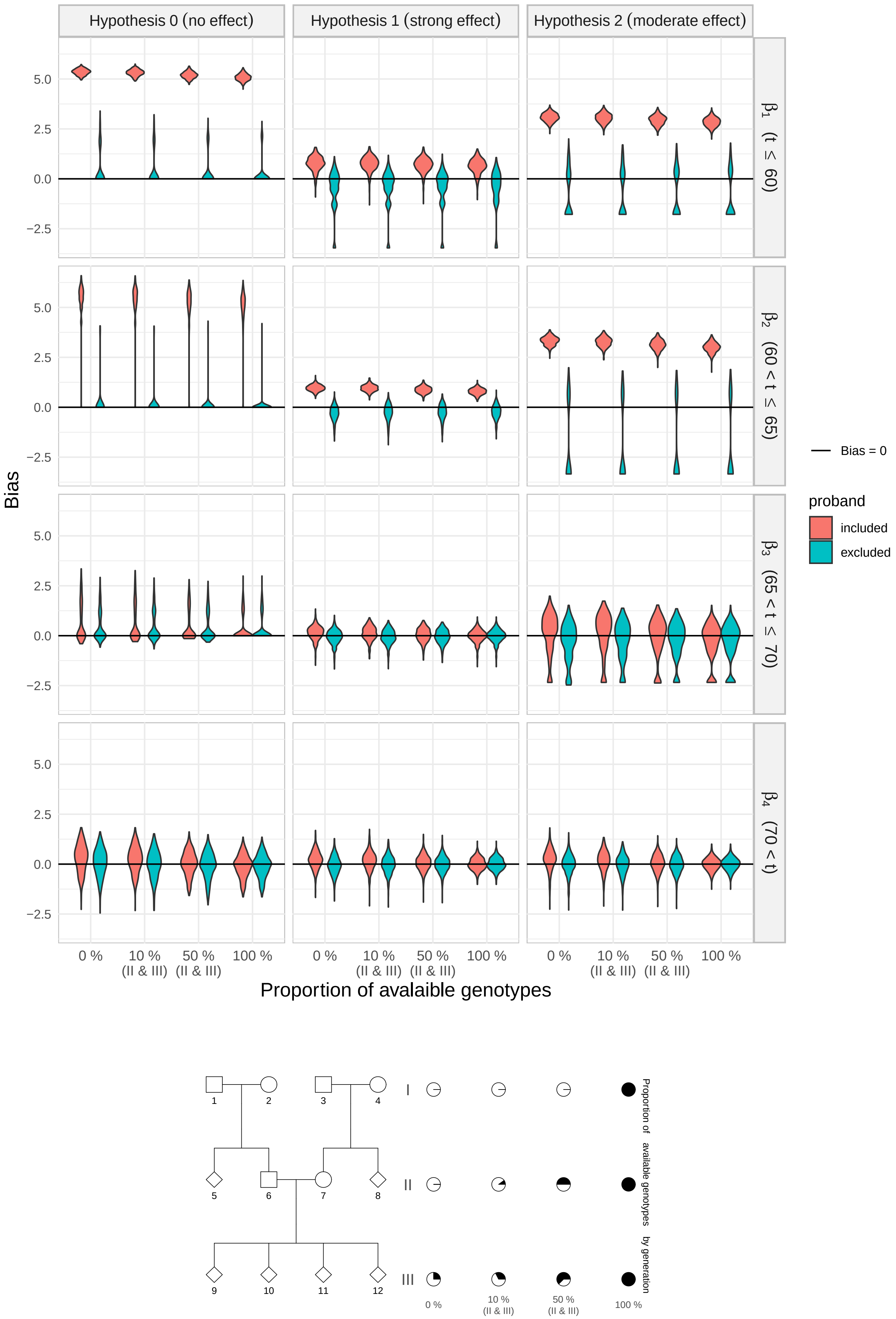

Fig. S3: Results of simulations in scenario A when missingness does not depend on age

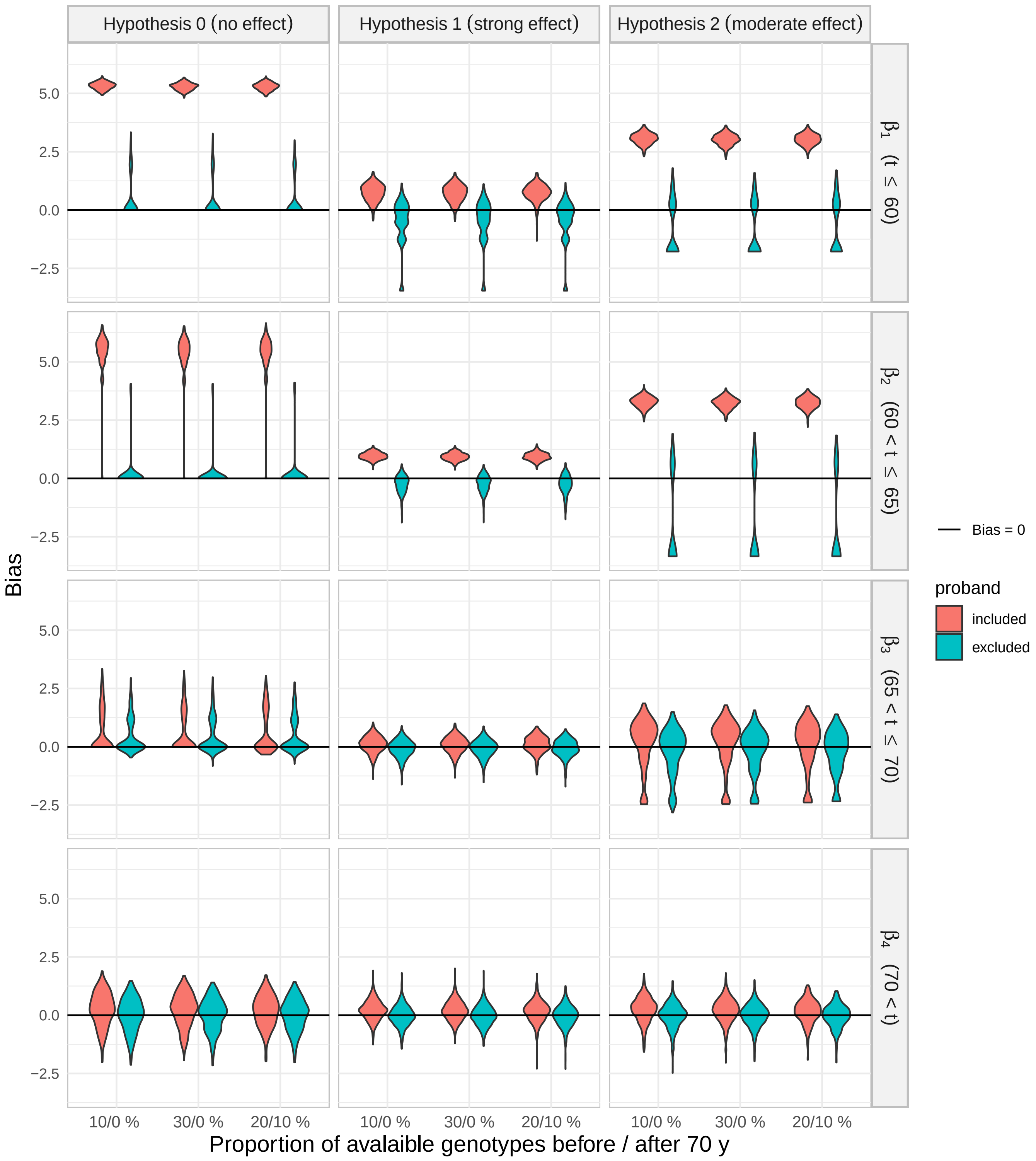

Fig. S4: Results of simulation in scenario A when missingness depends on age

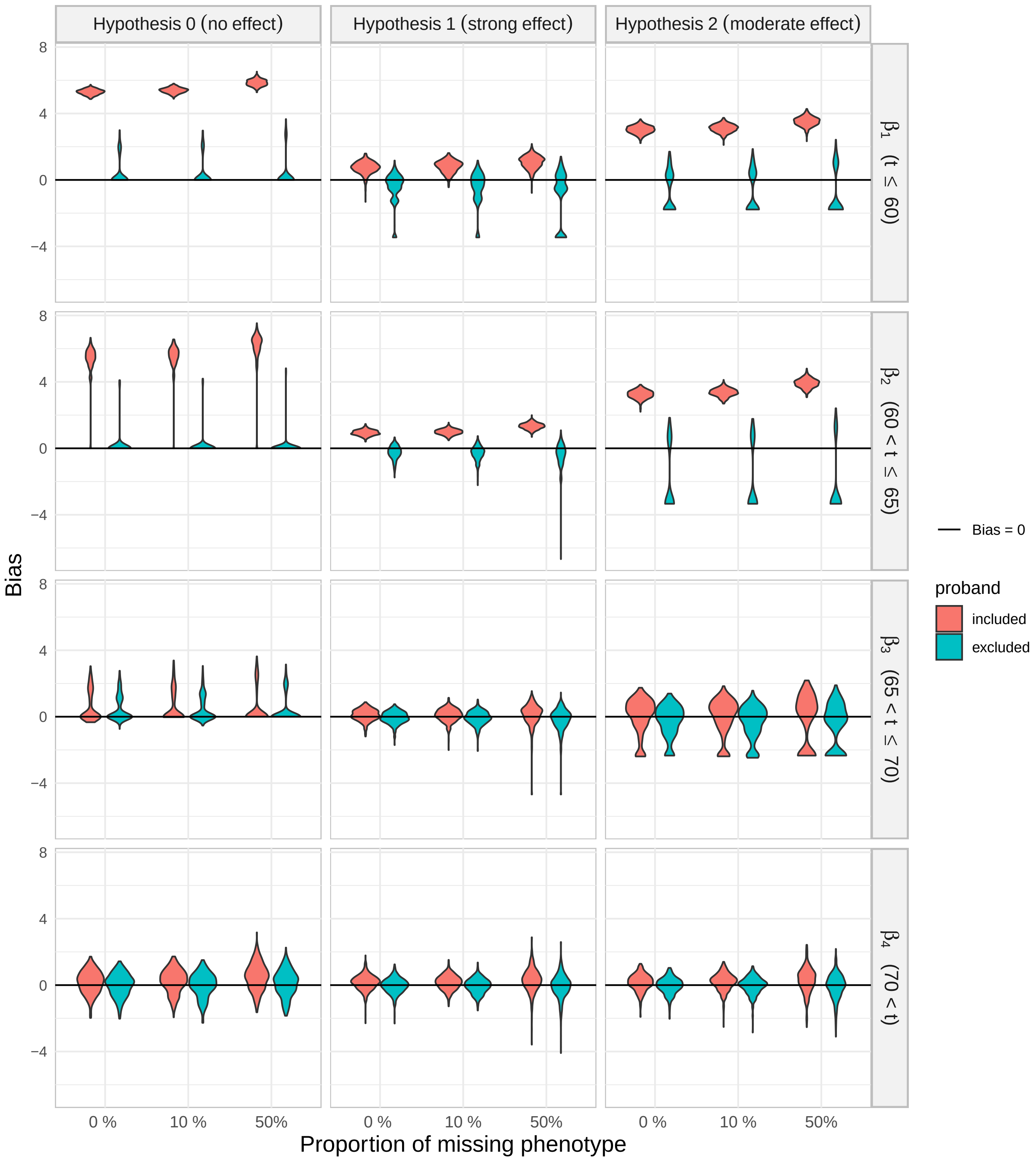

Fig. S5: Results of simulation in scenario B

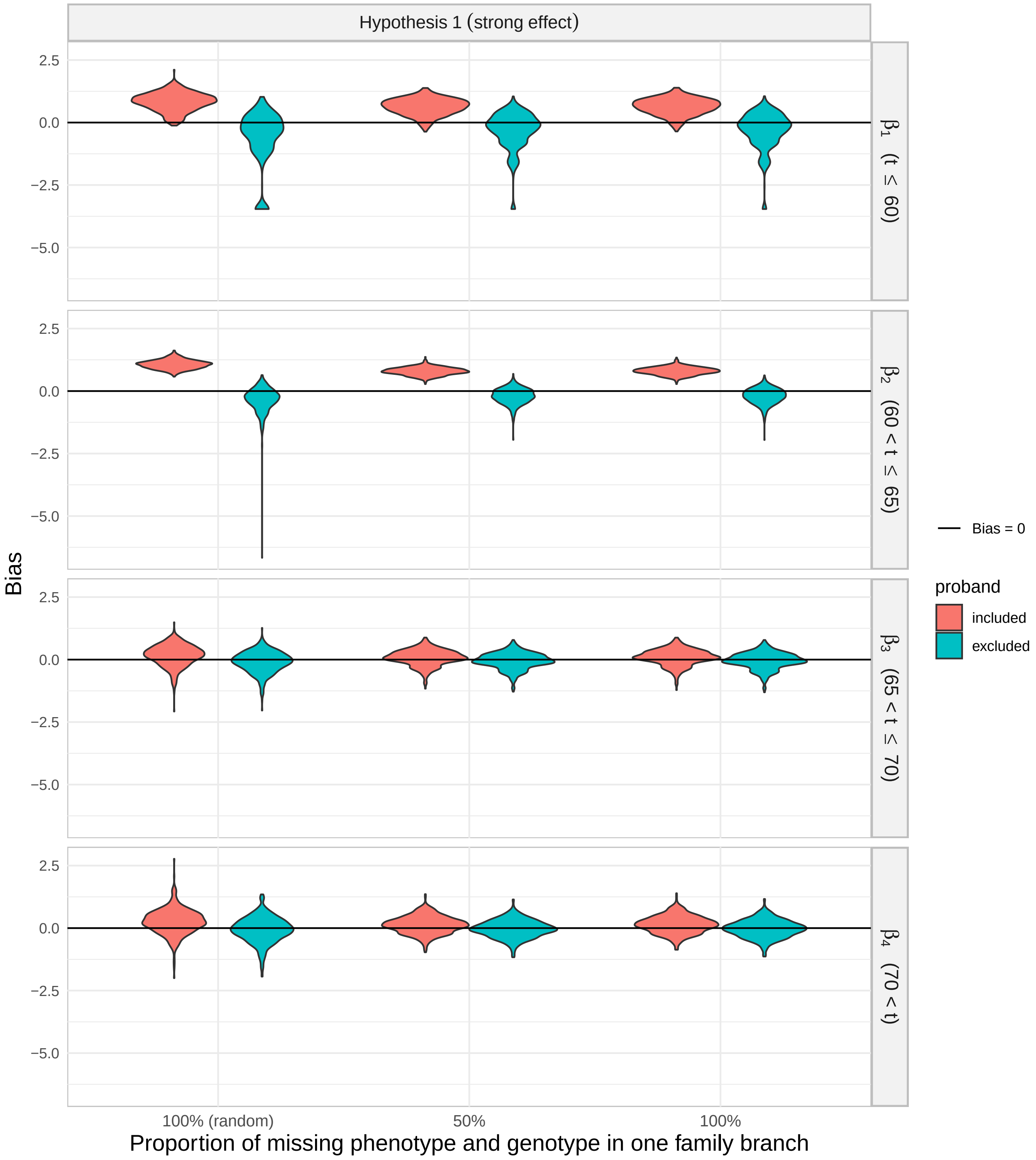

Fig. S6: Results of simulation in scenario B when missingness is related to one family branch only (the one with the lower number of cases)

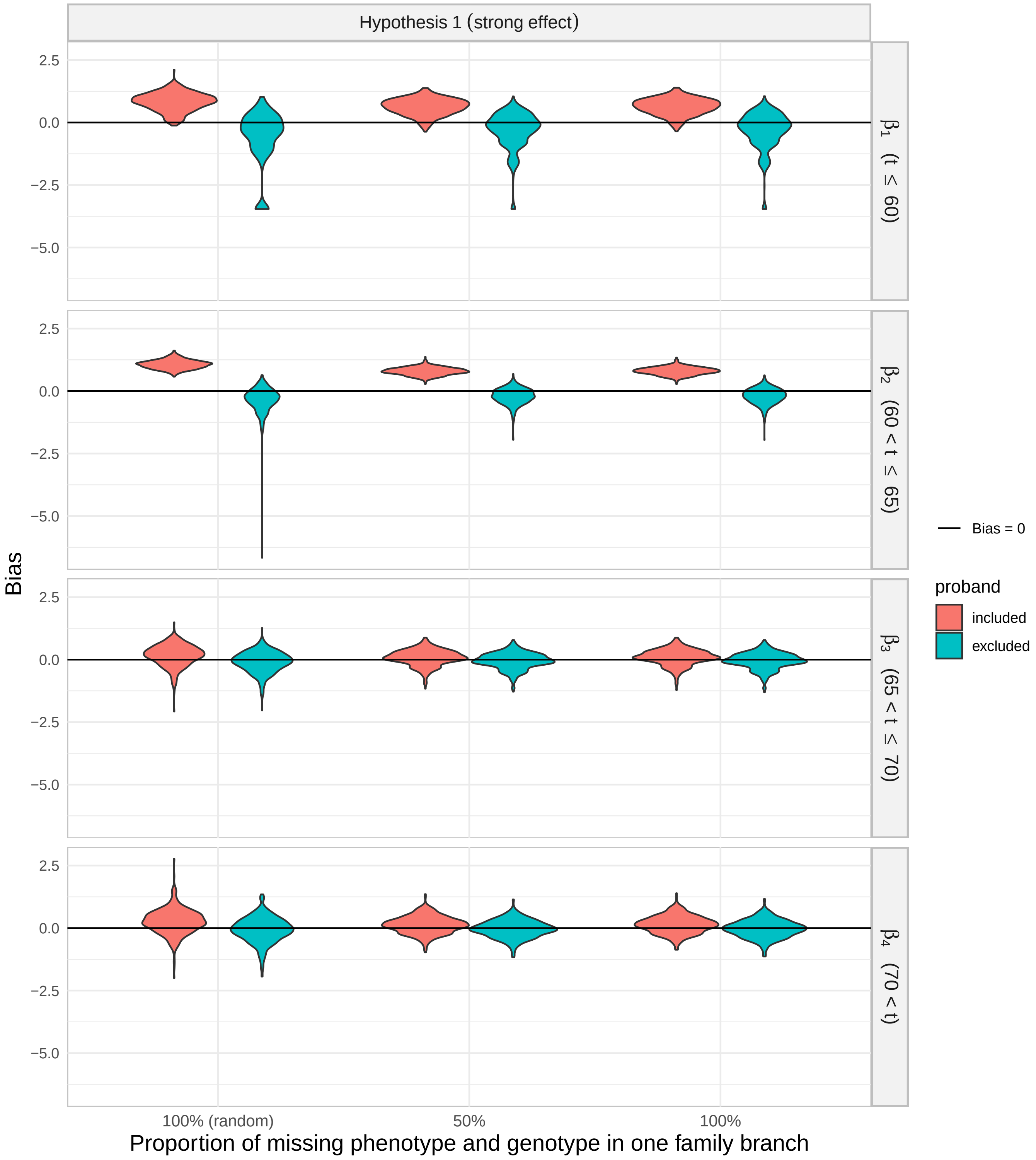

Fig. S7: Results of simulation in scenario B when missingness is related to one family branch only (the one with parent not being a case)

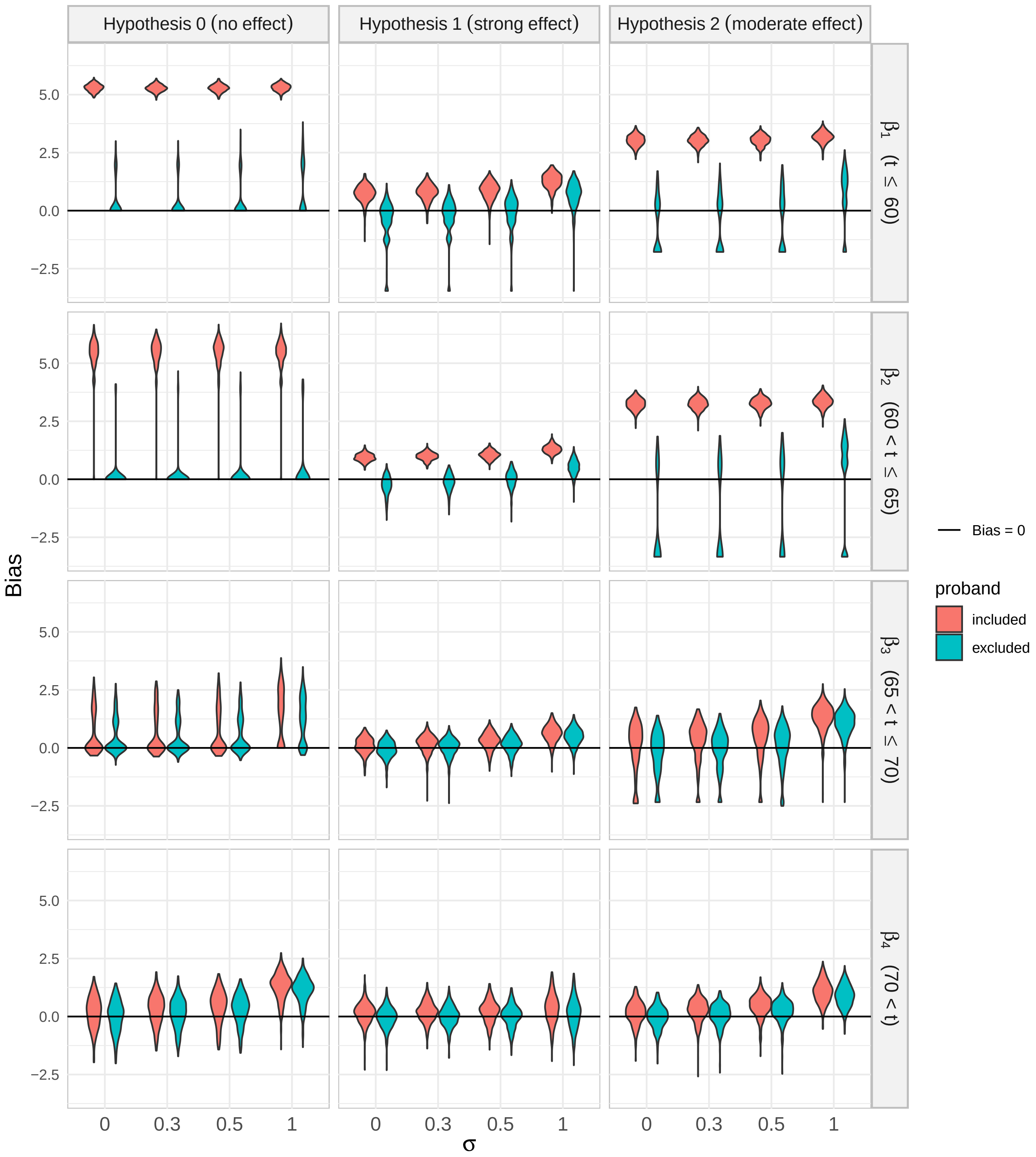

Fig. S8: Results of simulation in scenario C with normal random effect

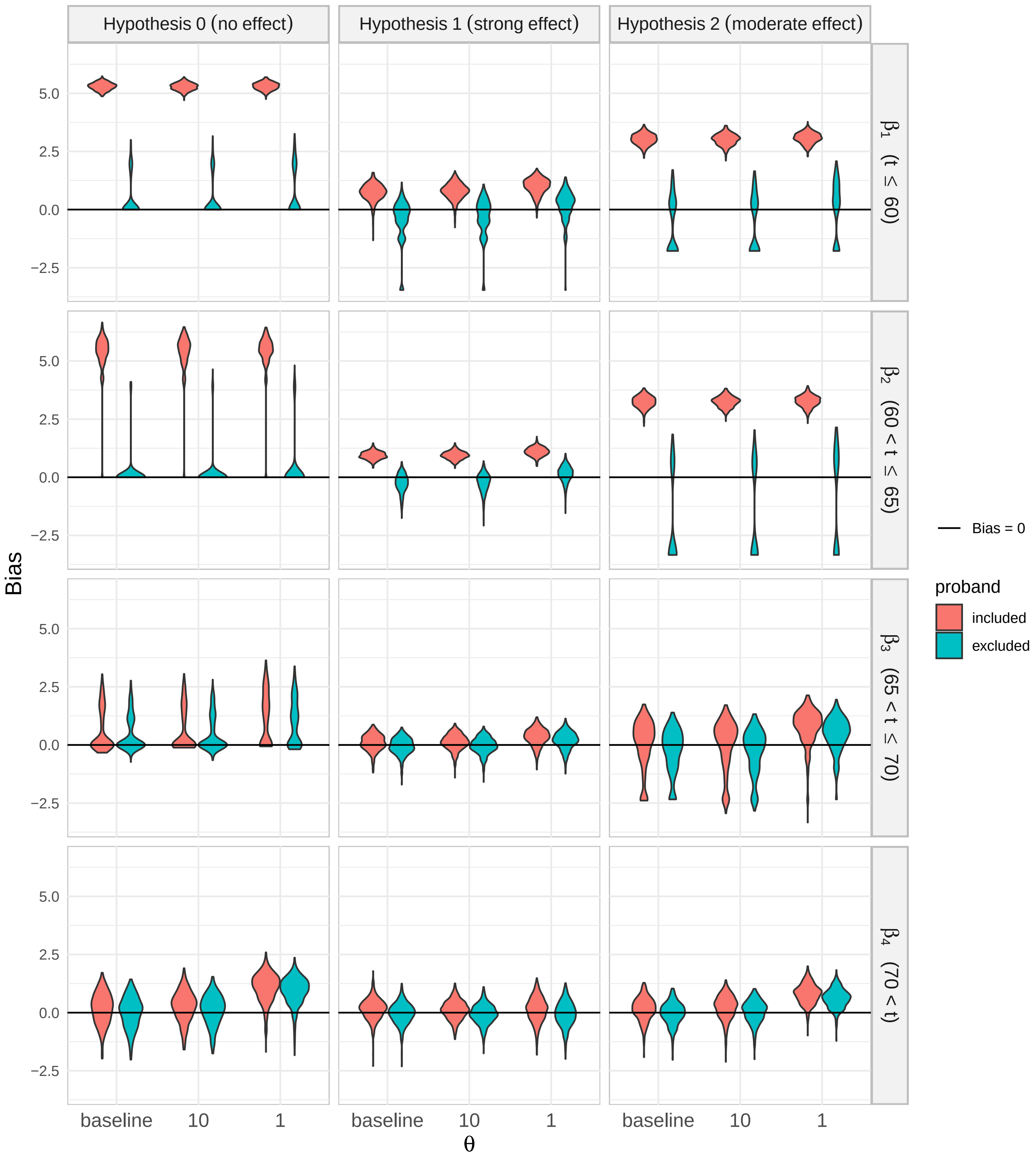

Fig. S9: Results of simulation in scenario C with gamma random effect

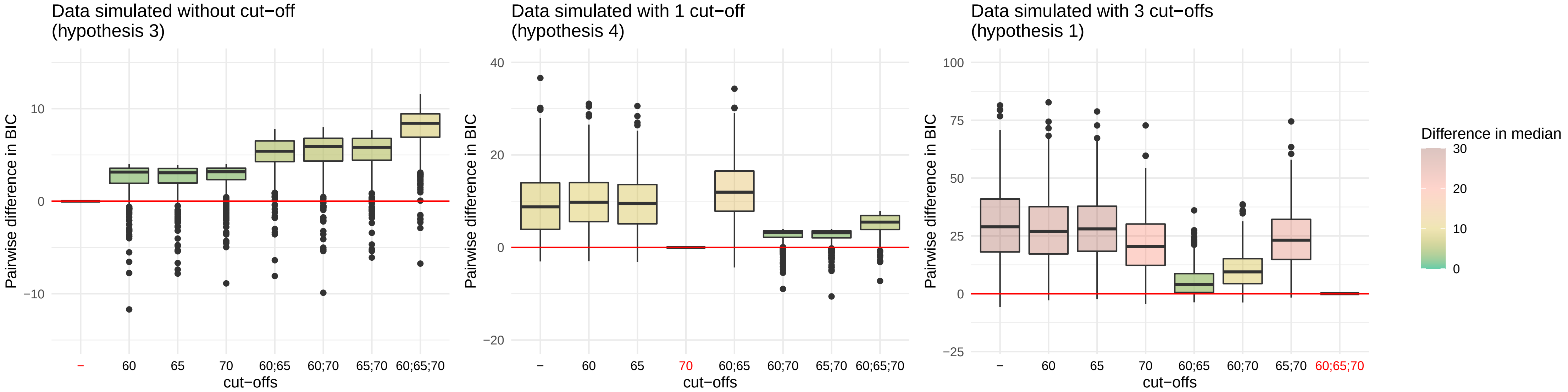

Fig. S10: Results of simulation in scenario D
Y-axis represents the pairwise difference in BIC between the one obtained from the model using cut-offs displayed on the X-axix and the one obtained from the true cut-off (in red). To better compare boxplot within each graphic, Y-axis were not displayed on the same scales.

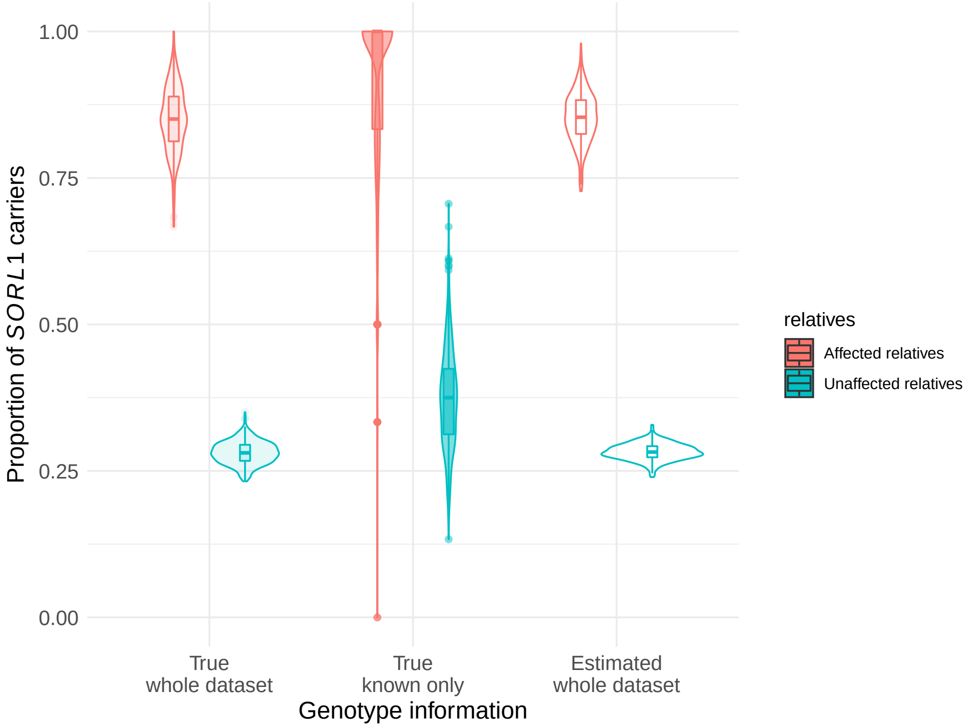

Fig. S11: Posterior probability of carrying a variant in the baseline *scenario* over 500 simulated datasets. This graphic represents the proportion of carriers of *SORL1* variant of interest according to genotype information. "True" refers to simulated genotypes and "estimated" refers to the sum of posterior weights obtained in the E-step of our algorithm.

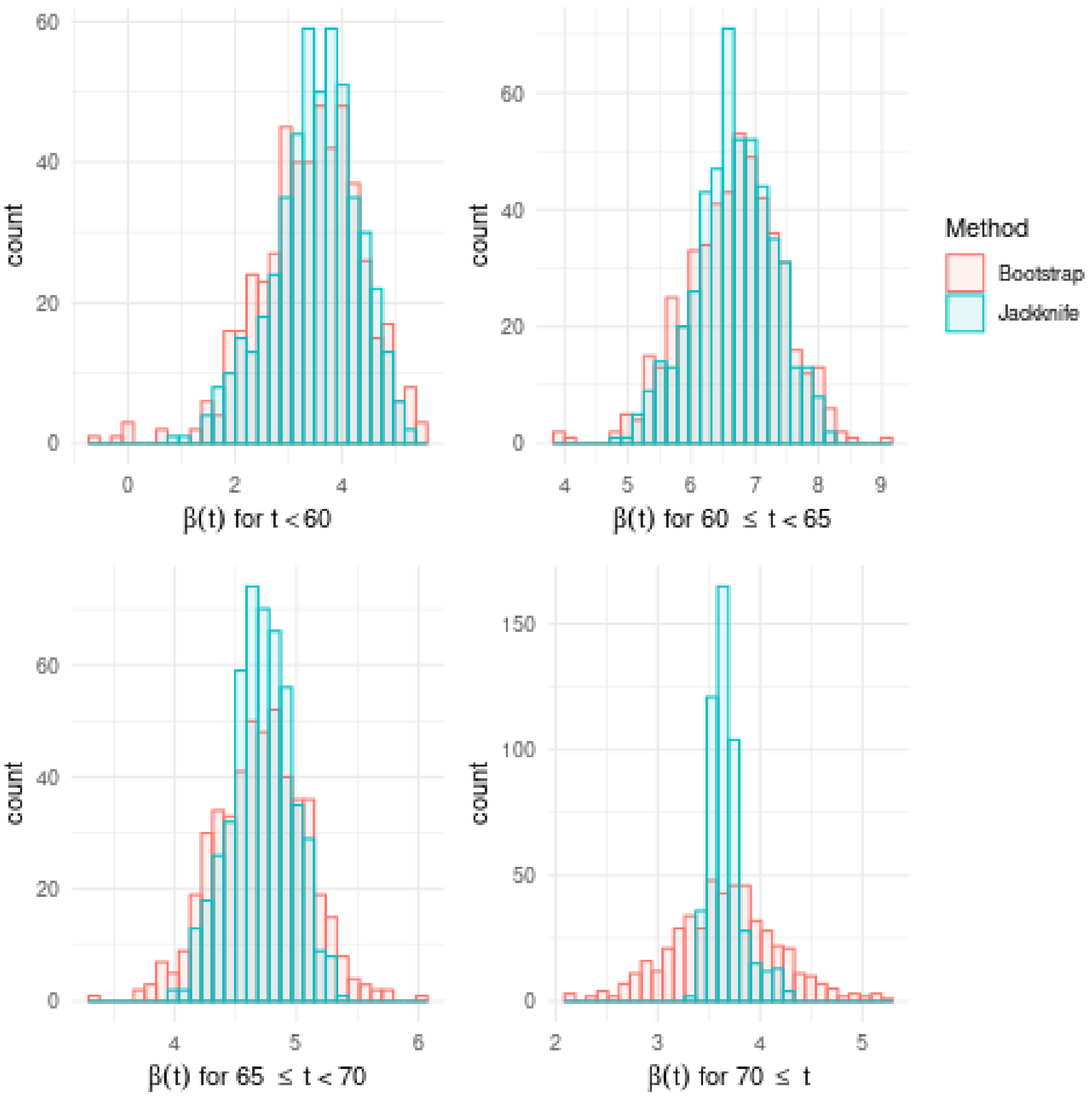

Fig. S12: Distribution of parameters obtained over 500 iterations of bootstrap and Jackknife methods.

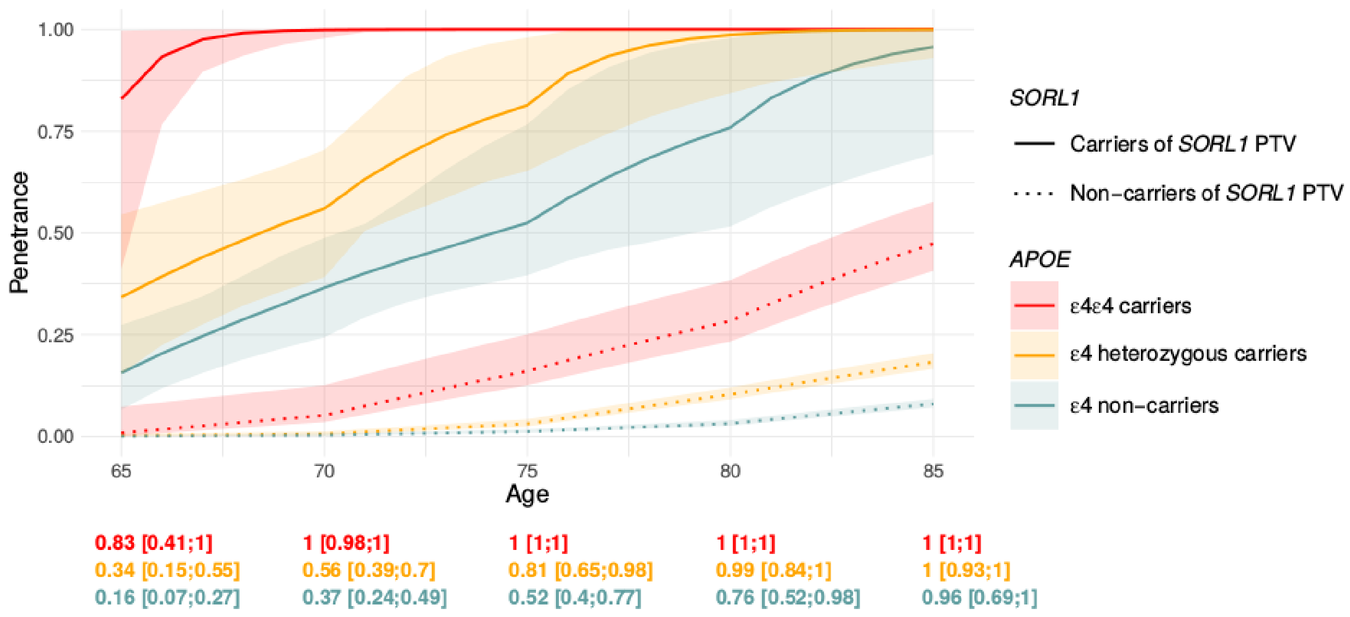

Fig. S13: Age dependent penetrance for carriers and non-carriers of a *SORL1* PTV.
The penetrance is displayed with 95% confidence interval according to the number of *APOE*-$\varepsilon 4$ allele from 65 to 85 years of age, for *SORL1* PTV carriers (after exclusion of Mis3-LoF variants). Curves for non-carriers and their confidence intervals were obtained from our estimation of $\lambda_{nc}(t \mid a)$ based on the Rotterdam study (Van der Lee *et al.*, *Lancet Neurol* 2018). Data from pedigrees were censored at 85 years. Confidence intervals for *SORL1* PTV carriers were obtained from 2.5^{th} and 97.5^{th} quantiles of 500 bootstrap iterations. Penetrance values at 65, 70, 75, 80, and 85 years of age, for carriers of *SORL1* PTV are displayed below the figure. Analysis for PTVs included 22 families carrying a *SORL1* PTV ($N = 241$ relatives > 40 years).

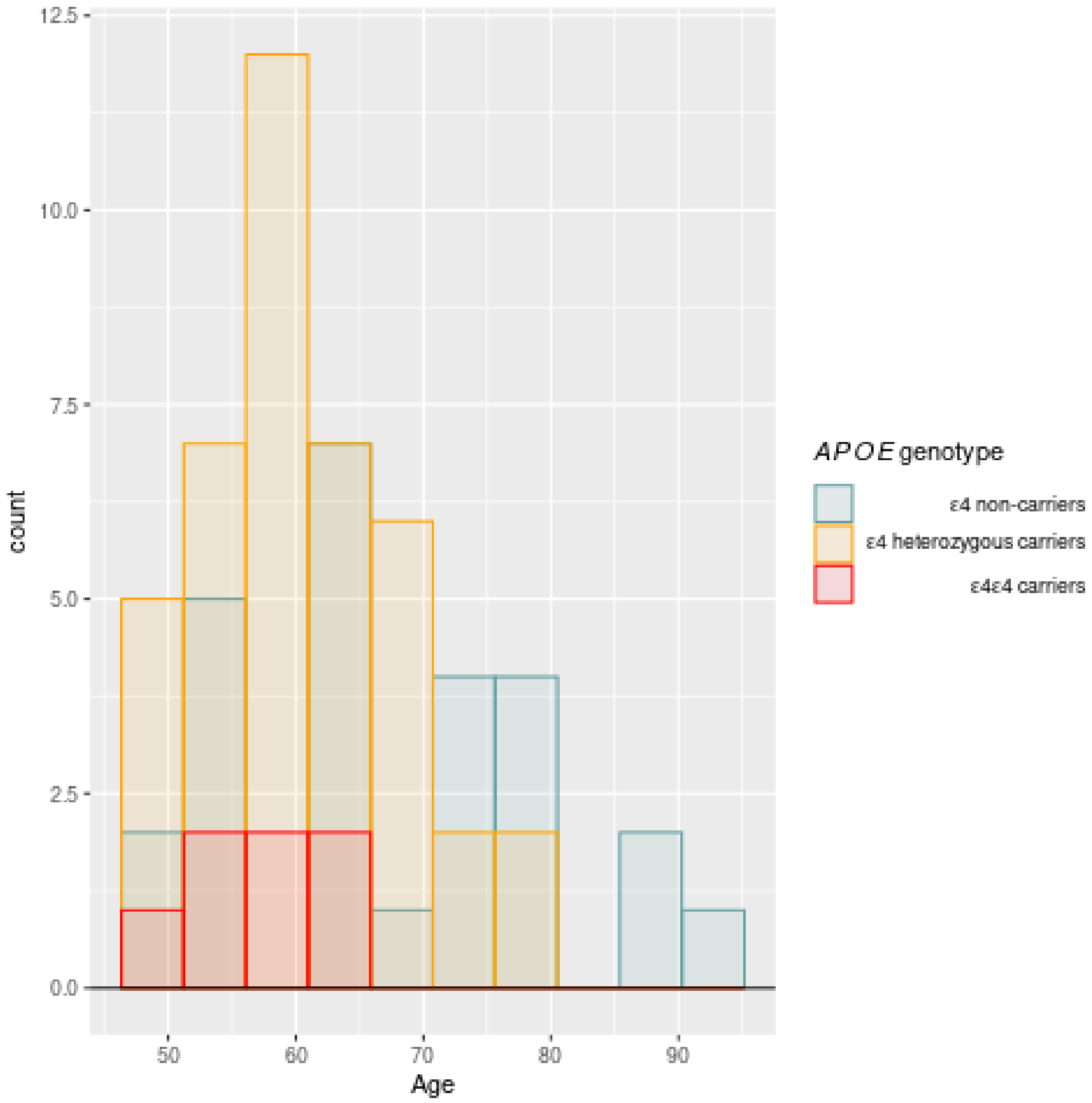

Fig. S14: Distribution of AAO for *SORL1*-LoF carriers being AD cases in the case-control dataset according to their *APOE* genotype.
Of notes, this distribution may not be considered as representative of the entire population of *SORL1*-LoF AD patients because of the selection bias towards larger proportion of cases and even early-onset patients in the case-control study.

Supplementary tables

Table S1: *SORL1* variants included in our family cohort

Type of variant		Coding sequence	Protein sequence	Families
PTV	nonsense	c.802C>T	p.R268*	ROU-0699
		c.2412G>A	p.W804*	EFA-0067
				EFA-0280
		c.2473C>T	p.Q823*	EXT-1893
		c.2596C>T	p.R866*	EXT-1906
		c.2795G>A	p.W932*	ROU-1766
		c.3619C>T	p.R1207*	EXT-1231
		c.3647G>A	p.W1216*	ROU-1409
		c.3805C>T	p.Q1269*	SAL-0621
		c.4434C>A	p.C1478*	EXT-0050
		c.4963C>T	p.R1655*	EXT-1106
		c.5395C>T	p.R1799*	EXT-1756
		c.5463G>A	p.W1821*	EXT-0017
		c.6163G>T	p.E2055*	EXT-0087
		c.6279C>G	p.Y2093*	EFA-0198
	frameshift	c.164delC	p.P55fs	EXT-0400
		c.1938delA	p.H647fs	ALZ-0167
		c.2603delC	p.T868fs	EXT-0438
		c.2882dupC	p.H962fs	EFA-0085
	splice	c.1211+2T>G		ROU-0055[a]
		c.3947-3insG		ROU-0055[a]
		c.4213+1G>A		RFA-0033
		c.4519+1G>A		EXT-1023
Mis3-LoF		c.994C>T	p.R332W	EXT-0290
		c.1531G>C	p.G511R	EXT-0049
		c.1960C>T	p.R654W	ROU-0309
				ROU-1622
				ROU-1376

PTV: protein-truncating variant; Mis3: Misense variant predicted as damaging by 3/3 software; LoF: loss-of-function; Mis3-LoF: Mis3 variant with *in vitro* LoF effect; a) Proband of ROU-0055 family is compound heterozygous for two distinct LoF variants (see Le Guennec et al.[9])

Table S2: Comparison of models based on the Bayesian Information Criterion (BIC).

Cut-offs				BIC	
				No censoring	Censoring
60				1173.41	1147.49
	65			1165.66	1148.52
		70		1155.37	1142.71
			75	1158.82	1149.04
60	65			1130.78	1113.25
60		70		1142.85	1129.81
60			75	1155.43	1145.47
	65	70		1159.26	1146.64
	65		75	1159.32	1149.70
		70	75	1155.54	1145.98
60	65	70		1124.24	1111.41
60	65		75	1124.30	1114.55
60		70	75	1143.23	1133.40
	65	70	75	1159.38	1149.85
60	65	70	75	1124.39	1114.71

Each row represents a model. Models differ in cut-offs for time interval definition in the piecewise constant function $\beta(t)$. No additional censoring was envisaged for our dataset. However, since only few individuals' observation time were greater that 85 years of age, we also compared BIC when data were censored at 85 years-old. Lower BIC means better fit. The same cut-offs were selected whether the data were censored or not censored (highlighted row).

Table S3: Expected total number of individuals by genotype.

	**Affected**		**Unaffected**	
	**N = 61**		**N = 246**	
SORL1 carriers, $\mathbb{E}(N)$(%)	48.46 (79.44%)		59.95 (24.37%)	
APOE × *SORL1* (+ vs WT)	+	WT	+	WT
ε4 non-carriers, $\mathbb{E}(N)$ (%)	21.03 (34.48%)	5.52 (9.04%)	38.57 (15.68%)	115.44 (46.93%)
ε4 heterozygous carriers, $\mathbb{E}(N)$ (%)	22.34 (36.63%)	5.88 (9.64%)	18.35 (7.46%)	63.89 (25.97%)
ε4ε4 carriers, $\mathbb{E}(N)$ (%)	5.08 (8.33%)	1.14 (1.88%)	3.02 (1.23%)	6.73 (2.73%)

The number of expected carriers $\mathbb{E}(N)$ of each genotype was estimated by summing posterior weights obtained in the E-step of our algorithm. Results are given for individuals with informative phenotype (i.e. status and age $\geq$ 40 years-old).

Supplementary references

References

1 van der Lee, S. J, Wolters, F. J, Ikram, M. K, Hofman, A, Ikram, M. A, Amin, N, et al. The effect of APOE and other common genetic vnariants on the onset of Alzheimer's disease and dementia: a community-based cohort study *The Lancet Neurology* 2018; *17*, 434–444.

2 Alarcon, F, Planté-Bordeneuve, V, Olsson, M, and Nuel, G Non-parametric estimation of survival in age-dependent genetic disease and application to the transthyretin-related hereditary amyloidosis *PloS one* 2018; *13*, e0203860.

3 Deville, J.-C, Särndal, C.-E, and Sautory, O Generalized raking procedures in survey sampling *Journal of the American statistical Association* 1993; *88*, 1013–1020.

4 Bouaziz, O and Nuel, G L0 regularisation for the estimation of piecewise constant hazard rates in survival analysis *arXiv preprint arXiv:1609.04595* 2016;.

5 Cox, D. R Regression models and life-tables *Journal of the Royal Statistical Society: Series B (Methodological)* 1972; *34*, 187–202.

6 Govindarajulu, U. S and D'Agostino Sr, R. B Review of current advances in survival analysis and frailty models *Wiley Interdisciplinary Reviews: Computational Statistics* 2020; *12*, e1504.

7 Ewbank, D. C Mortality differences by APOE genotype estimated from demographic synthesis *Genetic Epidemiology: The Official Publication of the International Genetic Epidemiology Society* 2002; *22*, 146–155.

8 Fine, J. P and Gray, R. J A proportional hazards model for the subdistribution of a competing risk *Journal of the American statistical association* 1999; *94*, 496–509.

9 Le Guennec, K, Tubeuf, H, Hannequin, D, Wallon, D, Quenez, O, Rousseau, S, et al. Biallelic loss of function of SORL1 in an early onset Alzheimer's disease patient *Journal of Alzheimer's Disease* 2018; *62*, 821–831.

Collaborator details

List of CNRMAJ (National Reference Center for Young Alzheimer Patients, *Centre National de Référence Malades Alzheimer Jeunes*) collaborators

Collaborators are displayed in alphabetic order with the following components: firstname (midle initials), lastname, institution, city, country

Daniela, Andriuta, Amiens university Hospital, Amiens, France;

Pierre, Anthony, Colmar Hospital, Colmar, France;

Sophie, Auriacombe, Bordeaux university Hospital, Bordeaux, France;

Anna-Chloé, Balageas, Tours university Hospital, Tours, France;

Guillaume, Ballan, Bayonne Hospital, Bayonne, France;

Mélanie, Barbay, Amiens university Hospital, Amiens, France;

Emilie, Beaufils, Tours university Hospital, Tours, France;

Yannick, Bejot, Dijon university Hospital, Dijon, France;

Serge, Belliard, Rennes university Hospital, Rennes, France;

Marie, Benaiteau, Toulouse university Hospital, Toulouse, France;

Karim, Bennys, Montpellier university Hospital, Montpellier, France;

Frédéric, Blanc, Strasbourg university Hospital, Strasbourg, France;

Stéphanie, Bombois, Pitié, Salpêtrière university Hospital, Paris, France;

Claire, Boutoleau Bretonnière, Nantes university Hospital, Nantes, France;

Pierre, Branger, Caen university Hospital, Caen, France;

Jasmine, Carlier, Toulouse university Hospital, Toulouse, France;

Leslie, Cartz-Piver, Limoges university Hospital, Limoges, France;

Pascaline, Cassagnaud, Lille university Hospital, Lille, France;

Giovanni, Castelnovo, Nimes university Hospital, Nimes, France;

Christine, Champion, Villeurbanne university Hospital, Villeurbanne, France;

Annabelle, Chaussenot, Nice university Hospital, Nice, France;

Mathieu, Ceccaldi, Marseille university Hospital, Marseille, France;

Valérie, Chauvire, Angers university Hospital, Angers, France;

Yaohua, Chen, Lille university Hospital, Lille, France;

Julien, Cogez, Caen university Hospital, Caen, France;

Emmanuel, Cognat, Lariboisière university Hospital, Paris, France;

Fabienne, Contegal-Callier, Beaune Hospital, Beaune, France;

Lea, Corneille, Marseille university Hospital, Marseille, France;

Philippe, Couratier, Limoges university Hospital, Limoges, France;

Hélène, Courtemanche, Nantes university Hospital, Nantes, France;

Benjamin, Cretin, Strasbourg university Hospital, Strasbourg, France;

Charlotte, Crinquette, Lille university Hospital, Lille, France;

Bernard, Croisille, Lyon university Hospital, Lyon, France;

Benjamin, Dauriat, Limoges university Hospital, Limoges, France;

Sophie, Dautricourt, Caen university Hospital, Caen, France;

Vincent, de la Sayette, Caen university Hospital, Caen, France;

Astrid, De liège, Pitié, Salpêtrière university Hospital, Paris, France;

Marie, De Verdal, Arles Hospital, Arles, France;

Didier, Deffond, Clermont-Ferrand university Hospital, Clermont-Ferrand, France;

Benoit, Delpont, Dijon university Hospital, Dijon, France;

Florence, Demurger, Rennes university Hospital, Rennes, France;

Vincent, Deramecourt, Lille university Hospital, Lille, France;

Céline, Derollez, Lille university Hospital, Lille, France;

Mira, Didic, Marseille university Hospital, Marseille, France;

Giulia, Diemert, Montpellier university Hospital, Montpellier, France;

Elsa, Dionet, Clermont-Ferrand university Hospital, Clermont-Ferrand, France;

Philippe, Diraison, Quimper Hospital, Quimper, France;

Aude, Doan, Rouen university Hospital, Rouen, France;

Martine, Doco Fenzy, Reims university Hospital, Reims, France;

Boris, Dufournet, Marseille university Hospital, Marseille, France;

Julien, Dumurgier, Lariboisière university Hospital, Paris, France;

Hélène, Durand, Strasbourg university Hospital, Strasbourg, France;

Anaïs, Dutray, Perpignan Hospital, Perpignan, France;

Frédérique, Etcharry-Bouyx, Angers university Hospital, Angers, France;

Maïté, Formaglio, Lyon university Hospital, Lyon, France;

Audrey, Gabelle, Montpellier university Hospital, Montpellier, France;

Anne, Gainche-Salmon, Rennes university Hospital, Rennes, France;

Jean-Claude, Getenet, Saint-Etienne university Hospital, Saint-Etienne, France;

Emmanuelle, Ginglinger, Mulhouse university Hospital, Mulhouse, France;

Olivier, Godefroy, Amiens university Hospital, Amiens, France;

Mathilde, Graber, Dijon university Hospital, Dijon, France;

Chloé, Gregoire, Bordeaux university Hospital, Bordeaux, France;

Stephan, Grimaldi, Marseille university Hospital, Marseille, France;

Julien, Gueniat, Dijon university Hospital, Dijon, France;

Claude, Gueriot, Marseille university Hospital, Marseille, France;

Sophie, Haffen, Besançon university Hospital, Besançon, France;

Lorraine, Hamelin, Sainte-Anne university Hospital, Paris, France;

Didier, Hannequin, Rouen university Hospital, Rouen, France;

Cezara, Hanta, Rennes university Hospital, Rennes, France;

Clémence, Hardy, Rouen university Hospital, Rouen, France;

Geoffroy, Hautecloque, Colmar Hospital, Colmar, France;

Camille, Heitz, Nimes university Hospital, Nimes, France;

Claire, Hourregue, Lariboisière university Hospital, Paris, France;

Thérèse, Jonveaux, Nancy university Hospital, Nancy, France;

Snejana, Jurici, Perpignan Hospital, Perpignan, France;

Catia, Khoumri, Dijon university Hospital, Dijon, France;

Lejla, Koric, Marseille university Hospital, Marseille, France;

Pierre, Krolak-Salmon, Villeurbanne Hospital, Villeurbanne, France;

Pierre, Labauge, Montpellier university Hospital, Montpellier, France;

Morgane, Lacour, Rouen university Hospital, Rouen, France;

Julien, Lagarde, Sainte-Anne university Hospital, Paris, France;

Hélène-Marie, Lanoiselée, Orléans Hospital, Orléans, France;

Brice, Laurens, Bordeaux university Hospital, Bordeaux, France;

Isabelle, Le Ber, Pitié, Salpêtrière university Hospital, Paris, France;

Gwenaël, Le Guyader, Poitiers university Hospital, Poitiers, France;

Amélie, Leblanc, Brest university Hospital, Brest, France;

Thibaud, Lebouvier, Lille university Hospital, Lille, France;

Anaïs, Lippi, Montpellier university Hospital, Montpellier, France;

Marie-Anne, Mackowiak, Lille university Hospital, Lille, France;

Eloi, Magnin, Besançon university Hospital, Besançon, France;

Cecilia, Marelli, Montpellier university Hospital, Montpellier, France;

Olivier, Martinaud, Caen university Hospital, Caen, France;

Aurélien, Maureille, Lille university Hospital, Lille, France;

Emilie, Milongo, Rigal, Toulouse university Hospital, Toulouse, France;

Sophie, Mohr, Dijon university Hospital, Dijon, France;

Hélène, Mollion, Lyon university Hospital, Lyon, France;

Olivier, Moreaud, Grenoble university Hospital, Grenoble, France;

Alexandre, Morin, Rouen university Hospital, Rouen, France;

Gaël, Nicolas, Rouen university Hospital, Rouen, FRANCE;

Julia, Nivelle, Ales Hospital, Ales, FRANCE;

Camille, Noiray, Sainte-Anne university Hospital, Paris, FRANCE;

Elisabeth, Ollagnon-Roman, Lyon university Hospital, Lyon, FRANCE;

Claire, Paquet, Lariboisière university Hospital, Paris, FRANCE;

Jérémie, Pariente, Toulouse university Hospital, Toulouse, FRANCE;

Florence, Pasquier, Lille university Hospital, Lille, FRANCE;

Alexandre, Perron, Amiens university Hospital, Amiens, FRANCE; ,

Nathalie, Philippi, Strasbourg university Hospital, Strasbourg, FRANCE;

Virginie, Pichon, Angers university Hospital, Angers, FRANCE;

Vincent, Planche, Bordeaux university Hospital, Bordeaux, FRANCE;

Céline, Poirsier, Reims university Hospital, Reims, FRANCE;

Marie, Rafiq, Toulouse university Hospital, Toulouse, FRANCE;

Pauline, Rod-Olivieri, Sainte-Anne university Hospital, Paris, FRANCE;

Adeline, Rollin-Sillaire, Lille university Hospital, Lille, FRANCE;

Carole, Roué-Jagot, Sainte-Anne university Hospital, Paris, FRANCE;

Dario, Saracino, Pitié-Salpêtrière university Hospital, Paris, FRANCE;

Marie, Sarazin, Sainte-Anne university Hospital, Paris, FRANCE;

Mathilde, Sauvée, Grenoble university Hospital, Grenoble, FRANCE;

François, Sellal, Colmar Hospital, Colmar, FRANCE;

Lila, Sirven Villaros, Nimes university Hospital, Nimes, FRANCE;

Christel, Thauvin, Dijon university Hospital, Dijon, FRANCE;

Camille, Tisserand, Toulouse university Hospital, Toulouse, FRANCE;

Christophe, Tomasino, Orléans Hospital, Orléans, FRANCE;

Cédric, Turpinat, Montpellier university Hospital, Montpellier, FRANCE;

Laurène, Van Damme, Perpignan university Hospital, Perpignan, FRANCE;

Olivier, Vercruysse, Saint-Brieuc Hospital, Saint-Brieuc, FRANCE;

Alice, Voilly, Besançon university Hospital, Besançon, FRANCE;

Nathalie, Wagemann, Nantes university Hospital, Nantes, FRANCE;

David, Wallon, Rouen university Hospital, Rouen, FRANCE;

Aline, Zarea, Rouen university Hospital, Rouen, FRANCE.

List of ADES (Alzheimer Disease European Sequencing project) collaborators

Collaborators are displayed in alphabetic order with the following components: firstname (midle initials), lastname, institution, city, country

Shahzad, Ahmad, Erasmus Medical Centre, Rotterdam, The Netherlands;

Najaf, Amin, Erasmus Medical Centre, Rotterdam, The Netherlands;

Philippe, Amouyel, Lille university Hospital, Lille, France;

Céline, Bellenguez, Pasteur Institute, Lille, France;

Claudine, Berr, Montpellier university, Montpellier, France;

Anne, Boland, Centre National de Recherche en Génomique Humaine, Evry, France;

Paola, Bossù, IRCCS Santa Lucia Foundation, Rome, Italy;

Femke, Bouwman, Vrije University, Amsterdam, The Netherlands;

Jose, Bras, Van Andel Institute, Grand Rapids, MI USA;

Dominique, Campion, Rouen university Hospital, Rouen, France;

Camille, Charbonnier, Rouen university Hospital, Rouen, France;

Jordi, Clarimon, Universitat Autònoma de Barcelona, Barcelona, Spain;

Antonio, Daniele, Catholic university of Sacred Heart, Rome, Italy; ,

Jean-François, Dartigues, Bordeaux Population Health Research Center, Bordeaux, France;

Stéphanie, Debette, Bordeaux Population Health Research Center, Bordeaux, France;

Jean-François, Deleuze, Centre National de Recherche en Génomique Humaine, Evry, France;

Nicola, Denning, Cardiff university, Cardiff, UK;

Oriol, Dols-Icardo, Universitat Autònoma de Barcelona, Barcelona, Spain;

Nick C., Fox, UCL Queen Square Institute of Neurology, London, UK;

Daniela, Galimberti, University of Milan, Milan, Italy; ,

Emmanuelle, Genin, Brest University, Brest, France;

Hans, Gille, Vrije University, Amsterdam, The Netherlands;

Benjamin, Grenier-Boley, Pasteur Institute, Lille, France;

Detelina, Grozeva, Cardiff University, Cardiff, UK;

Rita, Guerreiro, Van Andel Institute, Grand Rapids, MI USA;

John J., Hardy, UCL Institute of Neurology, London, UK; ,

Clive, Holmes, University of Southampton, Southampton, UK;

Henne, Holstege, Amsterdam university Hospital, Amsterdam, The Netherlands;

Marc, Hulsman, Amsterdam university Hospital, Amsterdam, The Netherlands;

Holger, Hummerich, UCL Institute of Prion Diseases, London, UK;

M. Arfan, Ikram, Erasmus Medical Centre, Rotterdam, The Netherlands; ,

M. Kamran, Ikram, Erasmus Medical Centre, Rotterdam, The Netherlands; ,

Iris, Jansen, Vrije University, Amsterdam, The Netherlands;

Amit, Kawalia, University of Cologne, Cologne, Germany;

Robert, Kraaij, Erasmus Medical Centre, Rotterdam, The Netherlands;

Jean-Charles, Lambert, Pasteur Institute, Lille, France;

Marc, Lathrop, McGill University, Montreal, QC Canada;

Afina W., Lemstra, Vrije University, Amsterdam, The Netherlands;

Alberto, Lleó, Universitat Autònoma de Barcelona, Barcelona, Spain;

Lauren, Luckcuck, Cardiff University, Cardiff, UK;

Marcel M.A.M., Mannens, University of Amsterdam, The Netherlands;

Rachel, Marshall, Cardiff University, Cardiff, UK;

Carlo, Masullo, Catholic University of the Sacred Heart, Rome, Italy;

Simon, Mead, UCL Institute of Prion Diseases, London, UK;

Patrizia, Mecocci, University of Perugia, Perugia, Italy;

Alun, Meggy, Cardiff university, Cardiff, UK;

Merel O., Mol, Erasmus Medical Centre, Rotterdam, The Netherlands;

Kevin, Morgan, University of Nottingham, Nottingham, UK;

Benedetta, Nacmias, University of Florence, Florence, Italy;

Gaël, Nicolas, Rouen university Hospital, Rouen, France;

Penny J., Norsworthy, UCL Institute of Prion Diseases, London, UK;

Florence, Pasquier, Lille university Hospital, Lille, France;

Pau, Pastor, Barcelona university Hospital, Barcelona, Spain;

Olivier, Quenez, Rouen university Hospital, Rouen, France;

Alfredo, Ramirez, University of Cologne, Cologne, Germany,

Rachel, Raybould, Cardiff university, Cardiff, UK;

Richard, Redon, Nantes university Hospital, Nantes, France;

Marcel, J.T., Reinders, Delft university of Technology, Delft, The Netherlands;

Anne-Claire, Richard, Rouen university Hospital, Rouen, France;

Steffi G., Riedel-Heller, University of Leipzig, Leipzig, Germany;

Fernando, Rivadeneira, Erasmus Medical Centre, Rotterdam, The Netherlands;

Stéphane, Rousseau, Rouen university Hospital, Rouen, France;

Natalie S., Ryan, UCL Queen Square Institute of Neurology, London, UK;

Salha, Saad, Cardiff University, Cardiff, UK;

Pascual, Sanchez-Juan, National Institute of Health Carlos III, Madrid, Spain;

Philip, Scheltens, Vrije University, Amsterdam, The Netherlands;

Jonathan M., Schott, UCL Queen Square Institute of Neurology, London, UK;

Davide, Seripa, Laboratory for Advanced Hematological Diagnostics, Lecce, Italy;

Daoud, Sie, Vrije University, Amsterdam, The Netherlands;

Rebecca, Sims, Cardiff University, Cardiff, UK;

Erik, Sistermans, Vrije University, Amsterdam, The Netherlands;

Sandro, Sorbi, University of Florence, Florence, Italy;

Resie, van Spaendonk, Vrije University, Amsterdam, The Netherlands;

Gianfranco, Spalleta, IRCCS Santa Lucia Foundation, Rome, Italy;

Niccólo, Tesi, Delft University of Technology, Delft, The Netherlands;

Betty, Tijms, Vrije University, Amsterdam, The Netherlands;

André G., Uitterlinden, Erasmus Medical Centre, Rotterdam, The Netherlands;

Wiesje M., van der Flier, Vrije University, Amsterdam, The Netherlands;

Sven J., van der Lee, Delft, University of Technology, Delft, The Netherlands;

Cornelia M., van Duijn, Erasmus Medical Centre, Rotterdam, The Netherlands;

Jeroen G.J., van Rooij, Erasmus Medical Centre, Rotterdam, The Netherlands;

John C., van Swieten, Erasmus Medical Centre, Rotterdam, The Netherlands;

Pieter J., de Visser, Vrije University, Amsterdam, The Netherlands;

Michael, Wagner, University of Bonn, Bonn, Germany;

David, Wallon, Rouen university Hospital, Rouen, France;

Julie, Williams, Cardiff University, Cardiff, UK;

Aline, Zarea, Rouen university Hospital, Rouen, France;

Alzheimer's Disease Neuroimaging Initiative (ADNI) database;

Alzheimer Disease Sequencing Project (ADSP).
